# Identification of novel secreted fatty acids that regulate nitrogen catabolite repression in fission yeast

**DOI:** 10.1038/srep20856

**Published:** 2016-02-19

**Authors:** Xiaoying Sun, Go Hirai, Masashi Ueki, Hiroshi Hirota, Qianqian Wang, Yayoi Hongo, Takemichi Nakamura, Yuki Hitora, Hidekazu Takahashi, Mikiko Sodeoka, Hiroyuki Osada, Makiko Hamamoto, Minoru Yoshida, Yoko Yashiroda

**Affiliations:** 1 Chemical Genetics Laboratory, RIKEN, Saitama, Japan; 2 Department of Life Sciences, Graduate School of Agriculture, Meiji University, Kanagawa, Japan; 3 Synthetic Organic Chemistry Laboratory, RIKEN, Saitama, Japan; 4 Catalysis and Integrated Research Group, RIKEN CSRS, Saitama, Japan; 5 Chemical Biology Research Group, RIKEN CSRS, Saitama, Japan; 6 Chemical Genomics Research Group, RIKEN CSRS, Saitama, Japan; 7 Molecular Structure Characterization Unit, Technology Platform Division, RIKEN CSRS, Saitama, Japan; 8 Department of Public Health, Yamaguchi University Graduate School of Medicine, Yamaguchi, Japan

## Abstract

Uptake of poor nitrogen sources such as branched-chain amino acids is repressed in the presence of high-quality nitrogen sources such as NH_4_^+^ and glutamate (Glu), which is called nitrogen catabolite repression. Amino acid auxotrophic mutants of the fission yeast *Schizosaccharomyces pombe* were unable to grow on minimal medium containing NH_4_Cl or Glu even when adequate amounts of required amino acids were supplied. However, growth of these mutant cells was recovered in the vicinity of colonies of the prototrophic strain, suggesting that the prototrophic cells secrete some substances that can restore uptake of amino acids by an unknown mechanism. We identified the novel fatty acids, 10(*R*)-acetoxy-8(*Z*)-octadecenoic acid and 10(*R*)-hydroxy-8(*Z*)-octadecenoic acid, as secreted active substances, referred to as Nitrogen Signaling Factors (NSFs). Synthetic NSFs were also able to shift nitrogen source utilization from high-quality to poor nitrogen sources to allow adaptive growth of the fission yeast amino acid auxotrophic mutants in the presence of high-quality nitrogen sources. Finally, we demonstrated that the Agp3 amino acid transporter was involved in the adaptive growth. The data highlight a novel intra-species communication system for adaptation to environmental nutritional conditions in fission yeast.

Living organisms are continuously exposed to variations in environmental factors, including nutrient availability. Therefore, in order to survive and prosper, they must adapt to environmental conditions. In yeast, expression of nitrogen metabolism genes is tightly regulated by the quality of available nitrogen sources. In the presence of high-quality nitrogen sources such as NH_4_^+^ and glutamate (Glu), the transporters/permeases that regulate the uptake of poorer nitrogen sources are down-regulated transcriptionally or post-transcriptionally^1^. The mechanism by which yeast preferentially utilizes high-quality nitrogen is called nitrogen catabolite repression^1^,^2^.

Fission yeast (*Schizosaccharomyces pombe*) Eca39 is a branched-chain amino acid aminotransferase, conserved among eukaryotes, that catalyzes the final step of the synthesis of the branched-chain amino acids isoleucine (Ile), leucine (Leu), and valine (Val). The auxotrophic mutant *eca39*Δ was unable to grow on minimal medium containing glutamate (EMM [Glu]) even when supplemented with Ile, Leu, and Val, because the uptake of these amino acids was suppressed in the presence of the high-quality nitrogen source Glu^3^. In previous work, however, we discovered a peculiar phenotype of adaptation to poor nitrogen sources. The growth of the *eca39*Δ mutant was restored when prototrophic cells were plated adjacent to the mutant cells^3^ (Fig. 1a), suggesting that some molecule(s) secreted from the growing cells allowed the mutant to switch its nitrogen source preference. This adaptive growth was not observed near budding yeast (*Saccharomyces cerevisiae*) cells, suggesting a species-specific phenomenon^3^ (Fig. 1a). Additionally, we observed that the Leu auxotrophic mutant *leu1* grew adaptively near other strains on media containing Leu and excess NH_4_^+^, suggesting that the growth defects of *eca39*Δ and *leu1* mutants were due to inhibition of uptake of required amino acids in the presence of high-quality nitrogen sources^3^. These observations raise the possibility that fission yeast cells secrete one or more diffusible, species-specific, signaling molecules that trigger an adaptive metabolic shift to utilization of branched-chain amino acids as nitrogen sources, allowing the growth of amino acid auxotrophic mutants^3^.

## Results

### Isolation of the active substances that regulate adaptive growth of the amino acid auxotrophic mutants

**Figure 1:**
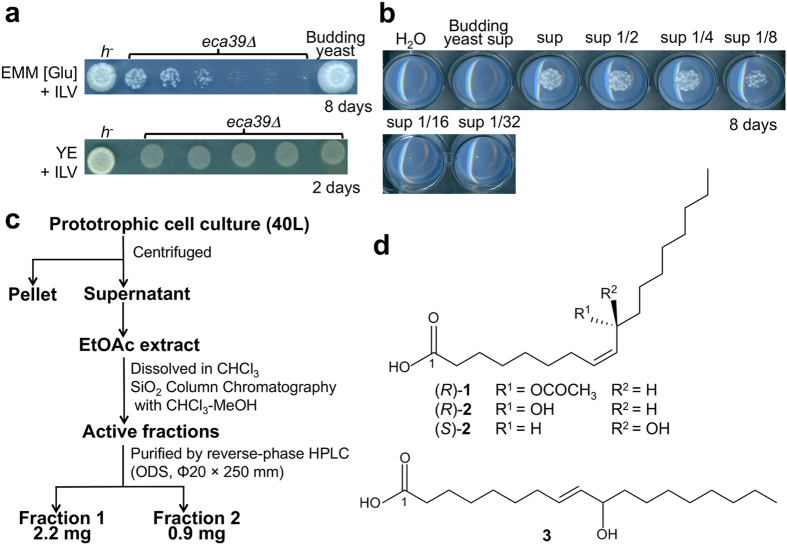
Fission yeast cells secrete active molecules to induce adaptive growth. (**a**) Adaptive growth of the *eca39*Δ mutant on minimal medium containing 15 mM Glu (EMM [Glu]). The *eca39*Δ mutant (SpHT257), prototrophic fission yeast (*h*^*−*^, SpHT219), and prototrophic budding yeast cells (YHT842) diluted at OD_600_ = 0.02 were spotted on EMM [Glu] supplemented with 2 mM each of Ile, Leu, and Val (EMM [Glu] + ILV) (top). *eca39*Δ mutant and prototrophic fission yeast cells diluted at OD_600_ = 0.4 were also spotted on rich yeast extract medium (YE) supplemented with 2 mM each of Ile, Leu, and Val (YE + ILV) (bottom). Plates were incubated at 30 °C for the indicated periods. (**b**) Activity of the supernatant of the prototrophic fission yeast strain that promotes adaptive growth of the *eca39*Δ cells. Prototrophic cells (SpHT219) were cultured in EMM [Glu] supplemented with 2 mM each of Ile, Leu, Val, Ade, and Ura (EMM [Glu] + ILV + AU), and 500 μL of the supernatant was filter-sterilized. The dried supernatant (sup) was dissolved in sterile water and a 2-fold dilution series was prepared for activity examination. The dilutions were layered onto solid EMM [Glu] + ILV + AU. Water (H_2_O) and supernatant of the budding yeast prototrophic cells (YHT842) were used as negative controls. *eca39*Δ cells (SpHT257) suspended in water at OD_600_ = 0.4 were spotted onto the media. Plates were incubated at 30 °C for 8 days. (**c**) Schematic isolation procedure for fractions 1 and 2. (**d**) Chemical structures of 10-acetoxy-8(*Z*)-octadecenoic acid (**1**), 10-hydroxy-8(*Z*)-octadecenoic acid (**2**), and 10-hydroxy-8(*E*)-octadecenoic acid (**3**).

To isolate the active substance from the cell culture, we developed a spot assay to assess the adaptive growth of the *eca39*Δ cells. This assay revealed that the supernatant of prototrophic cell cultures contained a potent activity capable of rescuing the growth of *eca39*Δ cells on EMM [Glu] (Fig. 1b). Small-scale fractionation revealed that active molecules were lipid-soluble and acidic (Fig. S1a,b). Next, we purified the active substance from the supernatant of prototrophic cells cultured in EMM [Glu] (40 L in total) by activity-guided fractionation, and obtained two active fractions: fraction 1 (2.2 mg) and fraction 2 (0.9 mg) (Fig. 1c). Serial dilution assays revealed that the minimum effective concentration (MEC) of the substance in fraction 1 was 24 ng/mL, whereas the MEC of the substance in fraction 2 was 98 ng/mL (Fig. S2).

### Structural elucidation of active substances

The chemical structures of the active compounds were determined by spectroscopic analyses by NMR, LC-MS, and GC-MS, revealing that they were two novel oxygenated fatty acids (oxylipins): fraction 1, 10(*R*)-acetoxy-8(*Z*)-octadecenoic acid ((*R*)-**1**), and fraction 2, 10(*R*)-hydroxy-8(*Z*)-octadecenoic acid ((*R*)-**2**) (Fig. 1d).

**Figure 2:**
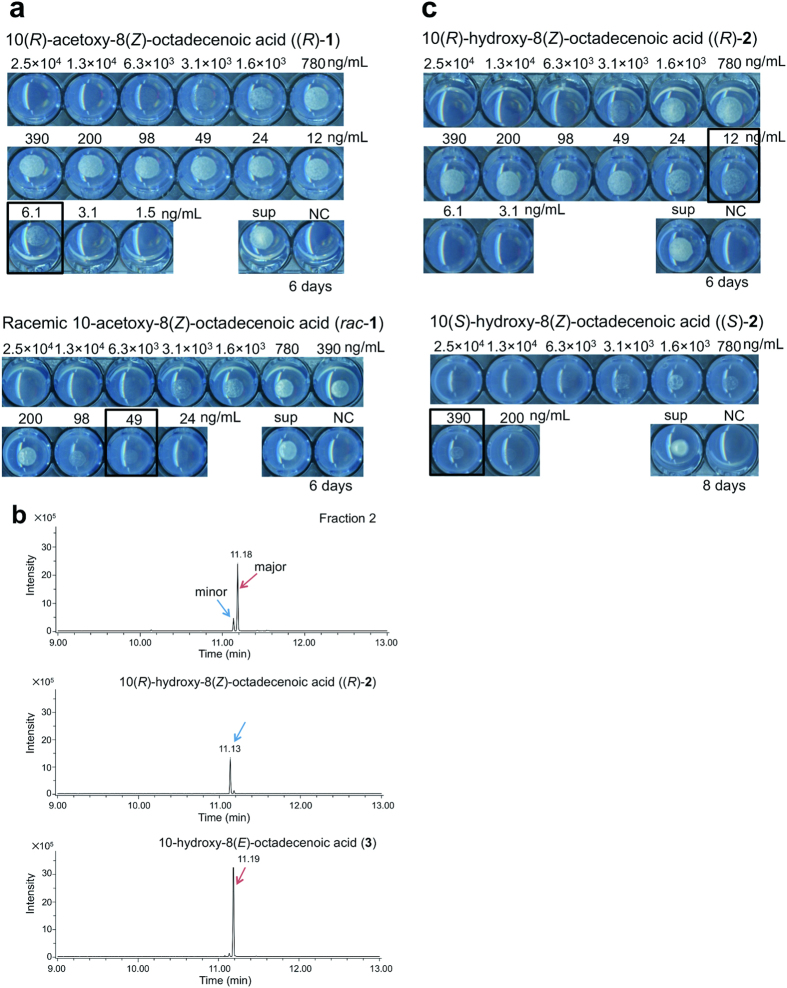
10(*R*)-acetoxy-8(*Z*)-octadecenoic acid and 10(*R*)-hydroxy-8(*Z*)-octadecenoic acid induce adaptive growth of the *eca39*Δ mutant. (**a**) Activity of synthetic 10(*R*)-acetoxy-8(*Z*)-octadecenoic acid ((*R*)-**1**) and synthetic racemic 10-acetoxy-8(*Z*)-octadecenoic acid (*rac*-**1**). Compounds were dissolved in 50% MeOH, and a 2-fold dilution series of the compounds was prepared with a starting concentration of 2.5 × 10^4^ ng mL^−1^ or 5.0 × 10^4^ ng mL^−1^ to examine activity. The dilutions were layered onto solid minimal media containing 15 mM Glu supplemented with 2 mM each of Ile, Leu, Val, Ade, and Ura (EMM [Glu] + ILV + AU). Supernatant of the prototrophic strain (SpHT219) (sup) and solvent (50% MeOH) were used as positive and negative controls (NC), respectively. *eca39*Δ cells (SpHT257) suspended in water at OD_600_ = 0.4 were spotted onto the solid media, and the plates were incubated at 30 °C for 6 days. Wells showing the minimum effective concentration (MEC) are marked with black rectangles. (**b**) Extracted ion chromatograms from GC-MS analyses of isolated fraction 2, 10(*R*)-hydroxy-8(*Z*)-octadecenoic acid ((*R*)-**2**), and 10-hydroxy-8(*E*)-octadecenoic acid (**3**). Major and minor peaks of isolated fraction 2 are marked with red and blue arrows, respectively (top). Peaks of (*R*)-**2** (middle) and **3** (bottom) are marked with blue and red arrows, respectively. All the samples were trimethylsilylated and subjected to GC-MS analysis. (**c**) Activity of synthetic 10(*R*)-hydroxy-8(*Z*)-octadecenoic acid ((*R*)-**2**) and 10(*S*)-hydroxy-8(*Z*)-octadecenoic acid ((*S*)-**2**). The activity of these compounds was monitored as described in (**a**). Plates were incubated at 30 °C for the indicated periods. Wells showing the MEC are marked with black rectangles.

The molecular formula of **1** was determined to be C_20_H_36_O_4_, according to the ESI-MS peak at *m/z* 339.2536 [M-H]^−^. The ^1^H and ^13^C NMR data of **1**, including 2D NMR spectra (Table S1) revealed a double bond (δ_H_ 5.53 and 5.29, δ_C_ 134.9 and 129.5), a carboxy group (δ_C_ 177.8), and an acetoxy group (δ_H_ 1.99, δ_C_ 21.2 and 172.2), suggesting that **1** is an acetoxyoctadecenoic acid. LC-MS/MS analysis of hydroxyoctadecenoic acid obtained from hydrolysis of **1** yielded a significant fragment ion peak at *m/z* 155.1 (Fig. S3a), indicating the position of the double bond at C-8. The double bond was deduced to be in the *Z*-form based on the coupling constant (*J*_8,9_) of 10.5 Hz in the ^1^H NMR spectrum (Table S1). The absolute configuration at C-10 was determined by a modified Mosher’s method^4^. Based on the distribution of Δδ (δ_*S*_ − δ_*R*_) values, C-10 was assigned the *R* configuration (Fig. S3b). Thus, **1** was determined to be 10(*R*)-acetoxy-8(*Z*)-octadecenoic acid ((*R*)-**1**) (Fig. 1d). The specific rotation value of this compound was [α]_D_^24.6^ −43° (0.063, MeOH). We synthesized (*R*)-**1** and racemic **1** (*rac*-**1**) (Supplementary Note) to confirm their abilities to induce the adaptive growth. The MEC of synthetic (*R*)-**1** was 6.1 ng/mL, whereas that of *rac*-**1** was 49 ng/mL (Fig. 2a), suggesting that the *R* configuration is essential for activity.

From fraction 2, we initially identified 10-hydroxy-8(*E*)-octadecenoic acid (**3**) (Fig. 1d) as the major compound by MS/MS (ESI), ^1^H and ^13^C NMR, and GC-MS (Fig. S4a, c and Table S2). However, synthetic racemic **3** (Supplementary Note) exhibited no activity in the spot assay (Fig. S4b), even when cells were incubated with **3** for 12 days, suggesting that other active compound(s) are present in fraction 2. By GC-MS analysis, we identified a minor peak near the major peak, whose MS spectrum did not differ significantly from that of the major peak (Fig. S4c). Therefore, we speculated that the minor peak might be 10-hydroxy-8(*Z*)-octadecenoic acid (**2**), and that this compound was the *bona fide* active substance in fraction 2. However, the quantity of material in the minor peak was too small to quantify and assess its activity. Therefore, we hydrolyzed compound (*R*)-**1** to obtain (*R*)-**2** (10(*R*)-hydroxy-8(*Z*)-octadecenoic acid). The resultant (*R*)-**2** had potent activity, with a MEC of 24 ng/mL (Fig. S4d). To prove the existence of **2** in fraction 2, we compared spectral data among (*R*)-**2**, synthetic racemic **3**, and fraction 2. No significant difference was observed in the MS/MS spectra between (*R*)-**2** and **3** (Fig. S5a). The HPLC retention time of the peak for (*R*)-**2** was identical to that of fraction 2 at T_R_ 14.5 (Fig. S5b). The GC-MS retention time of the minor peak of fraction 2 corresponded to that of (*R*)-**2,** whereas the retention time of the major peak of fraction 2 corresponded to that of synthetic **3** (Fig. 2b). No significant difference was observed in the MS spectra among the minor and major peaks of fraction 2, (*R*)-**2**, and **3** (Fig. S5c). These data suggest that the minor peak of fraction 2 is identical to 10-hydroxy-8(*Z*)-octadecenoic acid (**2**, Fig. 1d). We separately synthesized the *R*-isomer and the *S*-isomer of **2** (Supplementary Note), and the methylated version of these compounds were subjected to HPLC using a chiral column to identify the absolute configuration at C-10. The peak of methylated (*R*)-**2** corresponded to the peak at T_R_ 17 of fraction 2, whereas methylated (*S*)-**2** was not observed in fraction 2 by HPLC (Fig. S6). The ability of (*R*)-**2** to induce adaptive growth (MEC at 12 ng/mL) was much stronger than that of (*S*)-**2** (MEC at 390 ng/mL) (Fig. 2c). Taken together, these results indicate that the active substance in fraction 2 is 10(*R*)-hydroxy-8(*Z*)-octadecenoic acid ((*R*)-**2**).

To determine the structure–activity relationship, we examined the activities of six C18 fatty acids with structural similarity to the active compounds (*R*)-**1** and (*R*)-**2**: 10-hydroxy-8-octadecynoic acid (**4**), 10-hydroxy-octadecanoic acid (**5**), oleic acid (9(*Z*)-octadecenoic acid) (**6**), ricinoleic acid (12-hydroxy-9(*Z*)-octadecenoic acid) (**7**), 7,10-dihydroxy-8(*Z*)-octadecenoic acid (**8**), and 6-hydroxy-4(*E*)-octadecenoic acid (**9**) (Fig. S7). However, none of compounds **4**–**9** could induce adaptive growth of the *eca39*Δ mutant (Fig. S7). Therefore, the position of a *Z*-double bond at C-8 and an acetoxy or hydroxy group at C-10 in the *R* configuration are essential for activity.

### Identification of an amino acid transporter involved in adaptive growth of the amino acid auxotrophic mutants

**Figure 3:**
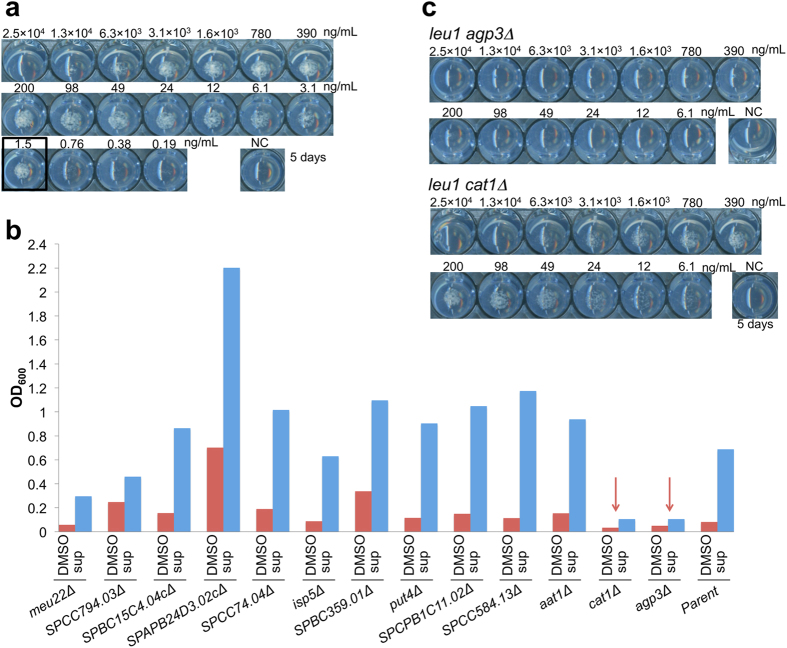
Adaptive growth of the Leu auxotrophic mutant is dependent on Agp3. (**a**) Activity of synthetic 10(*R*)-hydroxy-8(*Z*)-octadecenoic acid ((*R*)-**2**) on the *leu1* mutant. (*R*)-**2** was dissolved in 50% MeOH, and a 2-fold dilution series of the compound was prepared with a starting concentration of 2.5 × 10^4^ ng mL^−1^ to examine its activity. Solvent (50% MeOH) was used as a negative control (NC). Dilutions and solvent were layered onto solid minimal media containing 374 mM NH_4_Cl (EMM [374-N]) supplemented with 2 mM of Leu. *leu1* mutant cells (SpHT81) suspended in water at OD_600_ = 0.02 were spotted onto the solid media, and plates were incubated at 30 °C for 5 days. (**b**) Screening of amino acid transporter genes involved in adaptive growth. Amino acid transporter gene mutants (SpHT478-489, and 502) and their parental strain (SpHT227) were cultured in EMM containing 187 mM NH_4_Cl (EMM [187-N]) supplemented with 2 mM each of Ade, Ura, and Leu at 30 °C for 48 hours. Growth was monitored by measuring OD_600_ in the presence of DMSO or 0.1 mg mL^−1^ of the supernatant of the prototrophic cell culture (SpHT219) (sup). *cat1*Δ mutant and *agp3*Δ mutant are indicated by arrows. The experiment was performed twice, and a representative result is shown. (**c**) Activity of synthetic 10(*R*)-hydroxy-8(*Z*)-octadecenoic acid ((*R*)-**2**) on the *leu1 agp3*Δ mutant (XY-21) and the *leu1 cat1*Δ mutant (XY-23). The experiment was prepared as described in (**a**).

Adaptive growth has been observed not only in the *eca39*Δ mutant, but also in the Leu auxotrophic mutant *leu1*^3^. In EMM containing Leu and excess NH_4_Cl, growth of the *leu1* mutant was inhibited, but it was restored by the addition of supernatant of the prototrophic strain (Fig. S8a,b). By contrast, the growth of *ade6* and *ura4* single mutants was not promoted by supernatant of the prototrophic strain, suggesting that Leu uptake, but not Ade or Ura uptake, was specifically repressed by excess NH_4_Cl (Fig. S8b). We confirmed that both synthetic (*R*)-**2** and (*R*)-**1** isolated from fraction 1 induced adaptive growth of not only the *eca39*Δ mutant, but also the *leu1* mutant, at very low concentrations, 1.5 ng/mL and 3.1 ng/mL, respectively (Fig. 3a and Fig. S9a). Therefore, we refer to the compounds (*R*)-**1** and (*R*)-**2** as “Nitrogen Signaling Factors (NSFs)” that induce the adaptive uptake of branched-chain amino acids into fission yeast cells.

To identify the amino acid transporter involved in the adaptive growth of the *leu1* strain, we tested the effects of deletion mutations of 13 putative amino acid transporters in the *leu1 ade6 ura4* background on adaptive growth in EMM containing 187 mM NH_4_Cl (EMM [187-N]). The growth of all transporter mutants except *agp3*Δ and *cat1*Δ was remarkably increased by supplementation with the supernatant of the prototrophic strain (Fig. 3b and Fig. S8c). To rule out the possibility that Ade or Ura uptake had been affected, we constructed *agp3*Δ and *cat1*Δ mutants in the *leu1* background. The growth of these two mutants did not differ significantly from that of the parental *leu1* mutant in general EMM media (Fig. S8d). We confirmed that growth of the *agp3*Δ mutant was not rescued when synthetic (*R*)-**2** and (*R*)-**1** isolated from fraction 1 was added into EMM containing 374 mM NH_4_Cl (EMM [374-N]) supplemented with Leu, whereas growth of the *cat1*Δ mutant was rescued (Fig. 3c and Fig. S9b). These results demonstrate that the growth recovery of the *leu1* mutant was achieved by the Leu uptake via the Agp3 transporter. We previously reported that the transcriptional profile of adapted auxotrophic mutant cells is highly related to the profile of cells harboring deletions in the Spt-Ada-Gcn acetyltransferase (SAGA)^3^, a multiprotein chromatin modifying complex^5^. Indeed, Leu uptake activity is elevated in the *gcn5* mutant, and Agp3 is a downstream effector of this Leu uptake^3^. Our current results show that Agp3 is involved in the adaptive growth of the Leu auxotrophic mutant triggered by the NSFs. Further investigation is required to elucidate the link between the NSFs and the Gcn5 pathway.

## Discussion

In this study, we identified two structurally novel compounds, 10(*R*)-acetoxy-8(*Z*)-octadecenoic acid ((*R*)-**1**) and 10(*R*)-hydroxy-8(*Z*)-octadecenoic acid ((*R*)-**2**), as NSFs, secreted from fission yeast. Because (*R*)-**1** is an acetoxy derivative of (*R*)-**2**, it seems possible that (*R*)-**1** is a secreted form of (*R*)-**2** and is hydrolyzed to (*R*)-**2,** the *bona fide* signaling factor, after incorporation into the cell. Importantly, neither (*R*)-**1** nor (*R*)-**2** could induce adaptive growth at higher concentrations (Fig. 2a,c). This was not due to the toxicity, because even at quite high concentrations neither compound inhibited the growth of prototrophic cells (Fig. S10), suggesting that these compounds function only within a range of optimal concentrations. Because fission yeast cells contain a large amount of oleic acid, constituting around 75% of total fatty acids^6^, these auto-signaling molecules might be produced from oleic acid by a process that remains to be characterized.

We have provided ample evidence for the existence of a novel cell–cell communication system in fission yeast, mediated by the NSFs at very low effective concentrations (5–80 nM), that can liberate cells from nitrogen catabolite repression to allow uptake of poor nitrogen sources such as branched-chain amino acids instead of high-quality nitrogen. Like autoregulators in quorum-sensing, it seems likely that the NSFs mediate a quorum-sensing signal that makes fission yeast cells able to utilize poor nitrogen sources in late growth phases as the NSFs accumulate in culture, whereas they would utilize only high-quality nitrogen sources during early growth phase. Although the mechanism by which NSFs activate the pathway for the adaptive nitrogen metabolism remains elusive, we found that Agp3 is involved in Leu uptake in the adaptive growth of the amino acid auxotrophic mutant cells. It is of note that expression of the *agp3*^+^ gene was not significantly changed in the *eca39*Δ cells after adaptation, according to the microarray analysis in our previous study^3^. Thus, NSFs may induce Agp3 to be active as an amino acid transporter by a post-transcriptional mechanism.

Oxylipins play physiological roles in mammals and plants, but their importance in fungi, except the psi (precocious sexual inducer) factors for *Aspergillus nidulans*, remains ambiguous^7^,^8^,^9^,^10^. In *S. cerevisiae*, aromatic alcohols evoke quorum signaling for morphogenesis of an invasive filamentous form in response to cell density and the nutritional state in a species-specific manner^11^. Recently, it was reported that glucose repression in *S. cerevisiae* could be circumvented by signaling molecule(s) produced by bacteria, suggesting that chemical communication systems regulating nutrient metabolism are widespread in microbial communities including fungi^12^,^13^. Identification of the NSFs in this study opens the door for future work, including the mechanism by which the NSFs are sensed, and how the resultant signal is transduced to trigger the metabolic shift.

## Methods

### Strains and media

All strains used in this study are listed in Table S3. Haploid gene-deletion strains^14^ were purchased from Bioneer. Strains XY-21 and XY-23 were constructed using standard techniques, as described previously^15^. Rich yeast extract medium (YE) contained 0.5% yeast extract and 3% glucose. Edinburgh minimal medium (EMM) contained 93.5 mM (5 g/L) NH_4_Cl as a nitrogen source^16^. The pH of EMM media was adjusted to 5.5. Solid media contained 2% agar. EMM [Glu] refers to EMM supplemented with 15 mM Glu as a nitrogen source instead of NH_4_Cl, and is described in ref. 3. Media used were supplemented with 2 mM each of adenine (Ade), uracil (Ura), isoleucine (Ile), leucine (Leu), and valine (Val), as needed. Cells were regularly pre-grown in YE supplemented with Ade, Leu, and Ura. The *eca39*Δ mutant (SpHT257) was pre-grown in YE with Ile, Leu, and Val (ILV). For the Leu auxotrophic mutant *leu1* (SpHT81), a high concentration (187 mM or 374 mM) of NH_4_Cl was added to EMM medium (EMM [187-N] or EMM [374-N]) in addition to Leu.

### Sources of compounds described in this study

Compound (*R*)-**1** and (*R*)-**2** were isolated and purified from fractions 1 and 2, respectively. Compound **3** was isolated and purified from fraction 2. We synthesized compounds (*R*)-**1**, racemic **1** (*rac*-**1**), (*R*)-**2**, (*S*)-**2**, racemic **3**, **4**, and **5** according to established protocols as described in detail in the Supplementary Note. Compound **6** (≥98% purity) was purchased from Cayman Chemicals, **7** (≥99% purity) from Sigma, and **8** (≥95% purity) and **9** (≥85% purity) from AnalytiCon Discovery.

### Spot assay for assessment of adaptive growth

Solid EMM [Glu] medium or EMM [374-N] medium containing the required supplements was poured into 24- or 48-well plates. Prototrophic cell culture (SpHT219) supernatant extracted with ethyl acetate (EtOAc) or HPLC fractions were dried and redissolved in 50% methanol (MeOH), and a 2-fold serial dilution was prepared. Aliquots of the dilutions were poured onto solid medium in the appropriate wells, and then volatilized. Pre-grown *eca39*Δ mutant (SpHT257) or *leu1* mutant (SpHT81) cells were suspended in water at OD_600_ = 0.4 or 0.02, respectively, and 3 μl of suspension was spotted onto each well. The plates were incubated at 30 °C. Unless otherwise indicated, all spot assay experiments were performed twice, and representative results are shown in the figures.

### Isolation of fractions containing active molecules

To characterize chemical properties of active molecules, small-scale fractionation was carried out. Prototrophic cells (SpHT219) pre-cultured overnight on YE + ALU were inoculated into 10 mL of EMM [Glu] supplemented with ILV and AU (EMM [Glu] + ILV + AU) and grown at 30 °C for 2 days. Cultures were centrifuged and separated into supernatant and pellet. The supernatant was extracted with hexane, EtOAc, or butanol (BuOH), and then subjected to the spot assay described above. To isolate active molecules, pre-cultured prototrophic cells (SpHT219) were inoculated into EMM [Glu] + ILV + AU, and 40 L of cell culture was cultivated at 30 °C for 2 days. The culture was centrifuged at 5,000 × *g* for 10 minutes, and the supernatant was extracted with EtOAc. The EtOAc layer was dried using rotary evaporators at 35 °C. The resultant dried sample was subjected to silica gel (230–400 mesh, Merck) open-column chromatography followed by stepwise gradient elution with CHCl_3_–MeOH (v/v) (100:0, 99:1, 98:2, 97:3, 95:5, 93:7, 90:10, 80:20, 70:30, 50:50, and 0:100) as eluent. Each fraction was subjected to the spot assay to measure its activity. The most active fraction was further fractionated by HPLC (Waters 600) on a reverse-phase column (Pegasil ODS, Φ20 × 250 mm, Senshu Pak) using the following gradient program for elution (% MeOH in H_2_O): 80–100% (0–30 min) and 100% (30–40 min) at a flow rate of 8 mL/min. The resultant active fractions were pooled and fractionated using the same gradient program for elution (% CH_3_CN in H_2_O). Again, the resultant active fractions were pooled and fractionated using the following gradient program for elution (% MeOH in H_2_O): 85–100% (0–30 min) and 100% (30–40 min) at a flow rate of 9 mL/min. The active subfractions were collected to yield fraction 1 (2.2 mg), which was subjected to further analysis. Another active fraction was subjected to further fractionation by HPLC on a reverse-phase column using the following gradient program for elution (% MeOH in H_2_O): 50–100% (0–30 min) and 100% (30–40 min) at a flow rate of 8.5 mL/min. The active fraction was named fraction 2 (0.9 mg) and subjected to further analysis.

### Physicochemical measurements

NMR spectra were recorded on an ECA-500 FT-NMR spectrometer (JEOL) at 500 MHz for ^1^H-NMR and 125 MHz for ^13^C-NMR using CD_3_OD as the solvent. Chemical shifts were reported in ppm and referenced to the residual solvent signal. LC-MS (ESI) data was recorded on Synapt G2 (Waters). GC-MS (EI) data was recorded on JMS-T100GCV (JEOL). Specific rotations were recorded on a SEPA-300 polarimeter (HORIBA).

### Preparation of (*S*)- and (*R*)-MTPA esters of 10-acetoxy-8(*Z*)-octadecenoic acid

10-acetoxy-8(*Z*)-octadecenoic acid (**1**, 0.1 mg) was hydrolyzed and dissolved in a mixture of 100 μL of methanol and 200 μL of ether, and then 100 μL of 0.6 M (trimethylsilyl)diazomethane was added to the solution^17^. The reaction was monitored by TLC (silica gel 60F254, Merck; *n*-hexane-EtOAc, 4:1). The methylated compound was dried and redissolved in 50 μL of dehydrated pyridine, and 5 μL of (-)-Mosher’s acid chloride (Tokyo Kasei Kogyo) was added into the solution. The residue was subjected to silica gel column chromatography over *n*-hexane-EtOAc (4:1) to obtain the (*S*)-MTPA (2-methoxy-2-trifluoromethylphenylacetic acid) ester. The (*R*)-MTPA ester was obtained by the same procedure using ( + )-Mosher’s acid chloride (Tokyo Kasei Kogyo).

### Chiral separation of the compounds by HPLC

The synthetic *R*-isomer and *S*-isomer of 10-hydroxy-8(*Z*)-octadecenoic acid (**2**), synthetic 10-hydroxy-8(*E*)-octadecenoic acid (**3**), and fraction 2 were methylated as described above. The methylated compounds were separated by HPLC using a reverse chiral column (AD-RH, Φ0.46 × 15 cm, Daicel Chemical) with the following program for elution: 95% CH_3_CN-MeOH (5:1) in H_2_O (0–45 min) at a flow rate of 0.3 mL/min at ambient temperature.

### Examination of activity in liquid medium

Supernatant of prototrophic cells (SpHT219) was extracted with EtOAc, dried in a rotary evaporator at 35 °C, and then weighed. The resultant dried supernatant was dissolved in DMSO. Cells were inoculated at a starting concentration of OD_600_ = 0.01 in 2 mL of liquid medium with DMSO (1% v/v) or prototrophic cell culture supernatant (0.1, 0.25, or 0.5 mg/mL). Cells were grown at 30 °C for 2 or 3 days, and OD_600_ was measured every 12 hours.

## Additional Information

**How to cite this article**: Sun, X. *et al.* Identification of novel secreted fatty acids that regulate nitrogen catabolite repression in fission yeast. *Sci. Rep.* **6**, 20856; doi: 10.1038/srep20856 (2016).

## Supplementary Material

Supplementary Information
