# Supplementary material for: Identification of novel secreted fatty acids that regulate nitrogen catabolite repression in fission yeast
Source: Sci Rep. 2016 Feb 19;6:20856. doi: 10.1038/srep20856 (PMC4759587; doi:10.1038/srep20856)
Supplement: Supplementary Information [file srep20856-s1.pdf]

Supplementary Information

Identification of novel secreted fatty acids that regulate nitrogen catabolite repression in fission yeast

Xiaoying Sun[1,2], Go Hirai[3,4], Masashi Ueki[5,9], Hiroshi Hirota[6], Qianqian Wang[4], Yayoi Hongo[7], Takemichi Nakamura[7], Yuki Hitora[1], Hidekazu Takahashi[8], Mikiko Sodeoka[3,4], Hiroyuki Osada[5], Makiko Hamamoto[2], Minoru Yoshida[*,1,6] & Yoko Yashiroda[*,1,6]

[1]Chemical Genetics Laboratory, RIKEN, Saitama, Japan.
[2]Department of Life Sciences, Graduate School of Agriculture, Meiji University, Kanagawa, Japan.
[3]Synthetic Organic Chemistry Laboratory, RIKEN, Saitama, Japan.
[4]Catalysis and Integrated Research Group, RIKEN CSRS, Saitama, Japan.
[5]Chemical Biology Research Group, RIKEN CSRS, Saitama, Japan.
[6]Chemical Genomics Research Group, RIKEN CSRS, Saitama, Japan.
[7]Molecular Structure Characterization Unit, Technology Platform Division, RIKEN CSRS, Saitama, Japan.
[8]Department of Public Health, Yamaguchi University Graduate School of Medicine, Yamaguchi, Japan.
[9]Present address: Nano Medical Engineering Laboratory, RIKEN, Saitama, Japan.

*e-mail: yoshidam@riken.jp and ytyy@riken.jp

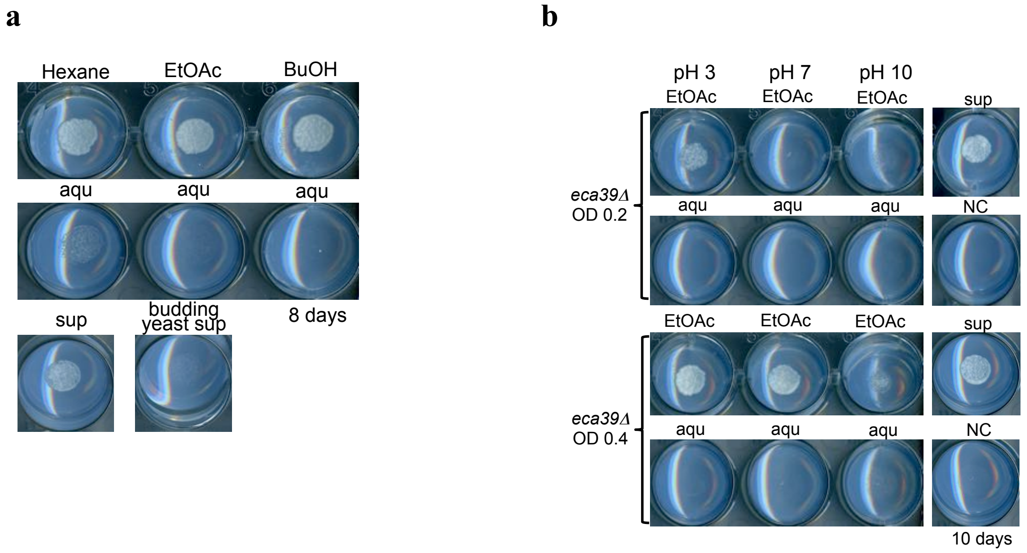

Figure S1. Characterization of active molecules in the culture supernatant of the prototrophic strain.

(**a**) Five hundred microliters of the supernatant of the prototrophic strain (SpHT219) cultured in EMM [Glu]+ILV+AU was extracted, separately with hexane, EtOAc, or BuOH. The organic layers and aqueous layers (aqu) were dried and redissolved separately in 50% MeOH to examine their activities. *eca39Δ* cells (SpHT257) suspended in water at OD_{600} = 0.2 were spotted onto EMM [Glu]+ILV+AU. Prototrophic fission yeast supernatant (sup) and budding yeast supernatant (budding yeast sup) were used as positive and negative controls, respectively. Plates were incubated at 30°C for 8 days. A representative result is shown; the experiment was performed three times. Based on the observation that the organic layers contained the activity that induced adaptive growth, we concluded that the active molecules were lipid-soluble. (**b**) Supernatant prepared as in (**a**) was adjusted to pH 3, 7, or 10, and then extracted with EtOAc. The organic layers and aqueous layers (aqu) were adjusted to a pH of 5.5. All of the layers were dried and redissolved separately in 50% MeOH to examine their activities. *eca39Δ* cells (SpHT257) suspended in water at OD_{600} = 0.2 or 0.4 were spotted onto EMM (Glu)+ILV+AU. Fission yeast prototrophic cell supernatant (sup) and solvent (50% MeOH) were used as positive and negative controls (NC), respectively. The plates were incubated at 30°C for 10 days. A representative result is shown; the experiment was performed three times. Based on the observation that the organic layers of supernatant adjusted to pH 3 had stronger activity, we concluded that the active molecules were acidic and lipid-soluble.

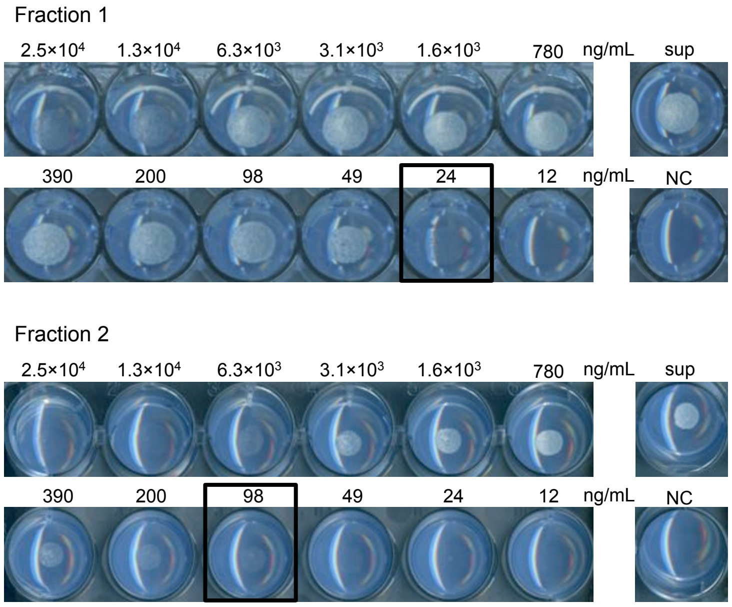

Figure S2. Adaptive growth–inducing activities of fractions 1 and 2.

Isolated fractions were dissolved in 50% MeOH, and a 2-fold dilution series of the compounds was prepared with a starting concentration of 2.5×10^4 ng/mL to examine the activities. Those dilutions were layered onto solid EMM [Glu]+ILV+AU. Supernatant of the prototrophic strain (SpHT219) (sup) and solvent (50% MeOH) were used as positive and negative controls (NC), respectively. *eca39Δ* cells (SpHT257) suspended in water at OD_{600} = 0.4 were spotted onto the solid media, and the plates were incubated at 30°C for 6 days. For fraction 1, the experiment was performed twice, and a representative result is shown. Due to the limited quantity of fraction 2, the experiment was performed only once.

a

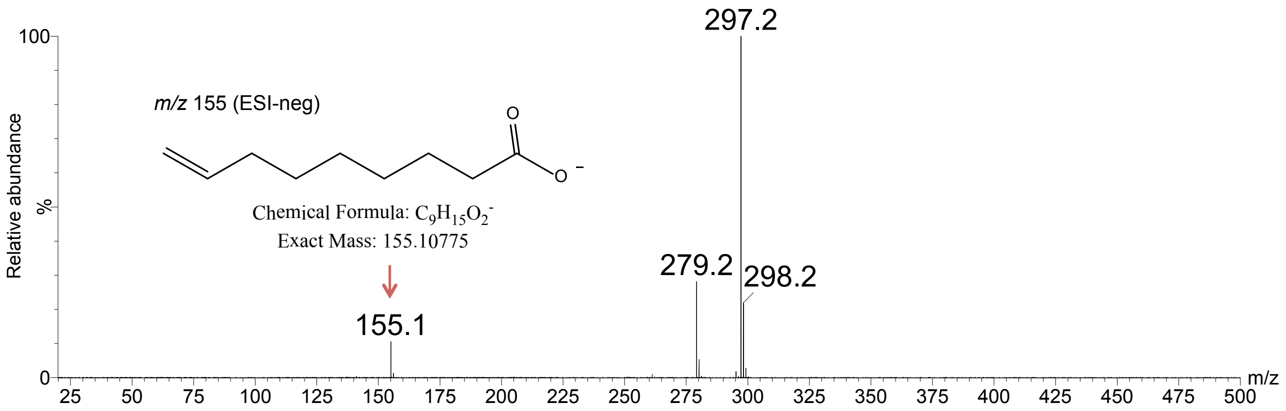

b

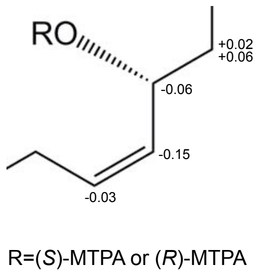

Figure S3. Identification of 10(*R*)-acetoxy-8(*Z*)-octadecenoic acid ((*R*)-**1**) in fraction 1.

(**a**) ESI-MS/MS spectrum (negative) of (*R*)-**1** in fraction 1. A significant fragmentation ion peak was observed at *m/z* 155.1 (arrow), indicating the double bond positioned at C-8. (**b**) ^{1}H NMR chemical shift difference of the Mosher esters ($\Delta\delta = \delta_S - \delta_R$).

a

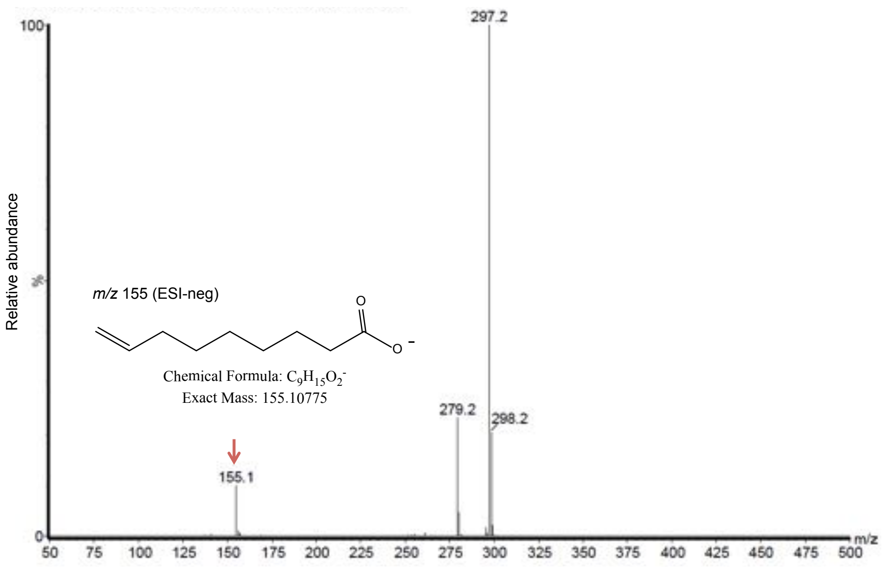

b

Racemic 10-hydroxy-8(*E*)-octadecenoic acid (**3**)

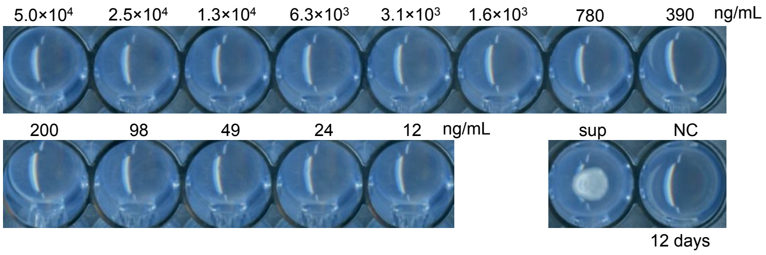

c

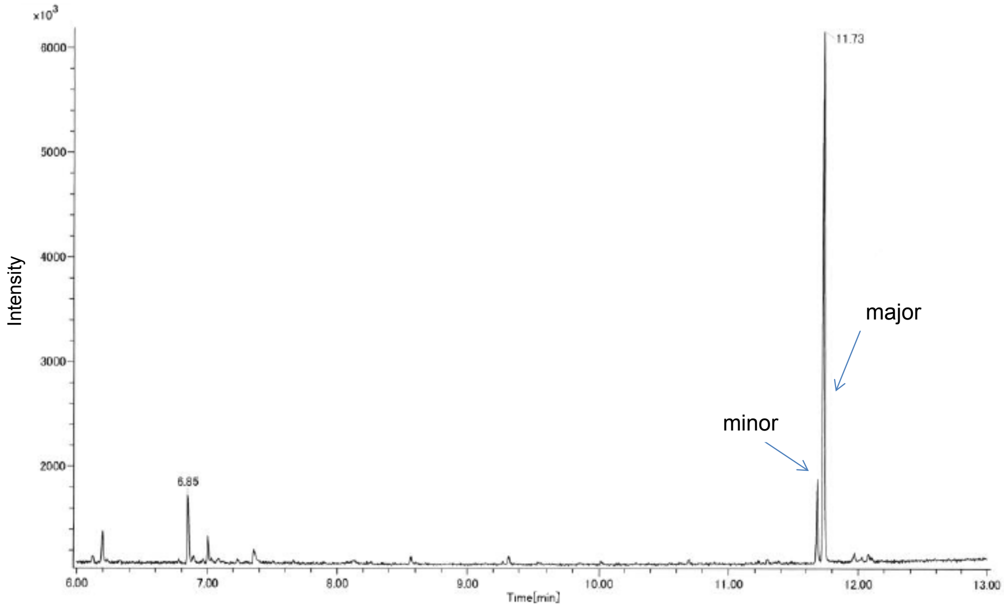

c (continuted)

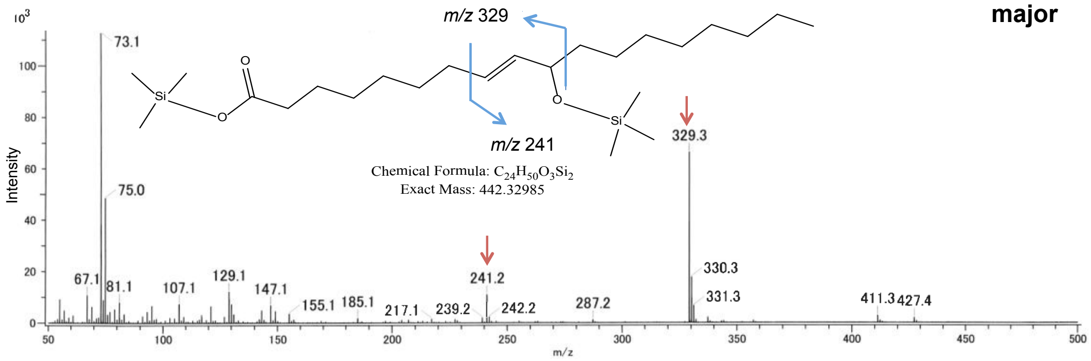

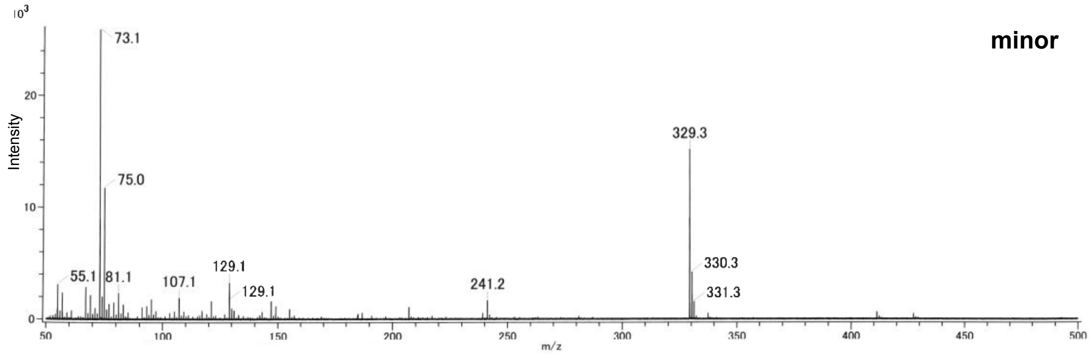

d

10(*R*)-hydroxy-8(*Z*)-octadecenoic acid ((*R*)-**2**)

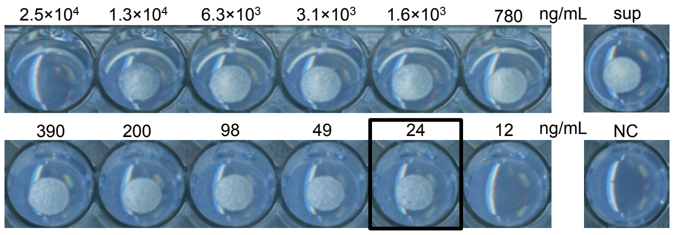

Figure S4. Identification of 10-hydroxy-8(*E*)-octadecenoic acid (**3**) in fraction 2.

(**a**) ESI-MS/MS spectrum (negative) of fraction 2. A significant fragmentation ion peak was observed at *m/z* 155.1 (arrow), indicating the double bond positioned at C-8. (**b**) Activity of synthetic racemic 10-hydroxy-8(*E*)-octadecenoic acid (**3**). The compound was dissolved in 50% MeOH, and a 2-fold dilution series was prepared with a starting concentration of 5.0×10^4 ng/mL to examine the activity by spot assay. The dilutions were layered onto solid EMM [Glu]+ILV+AU. The supernatant of the prototrophic strain (SpHT219) (sup) and solvent (50% MeOH) were used as positive control and negative controls (NC), respectively. Plates were incubated at 30°C for 12 days. The experiment was performed twice, and a representative result is shown. (**c**) GC-MS chromatogram of fraction 2 and mass spectra of the major peak and the minor peak. The fraction 2 sample was trimethylsilylated and subjected to GC-MS analysis. (**d**) Activity of 10(*R*)-hydroxy-8(*Z*)-octadecenoic acid ((*R*)-**2**) obtained by hydrolyzing (*R*)-**1**. The activity of the compounds was monitored as described in (**b**) and the plates were incubated at 30°C for 6 days. Well showing the MEC is indicated with a black rectangle.

a

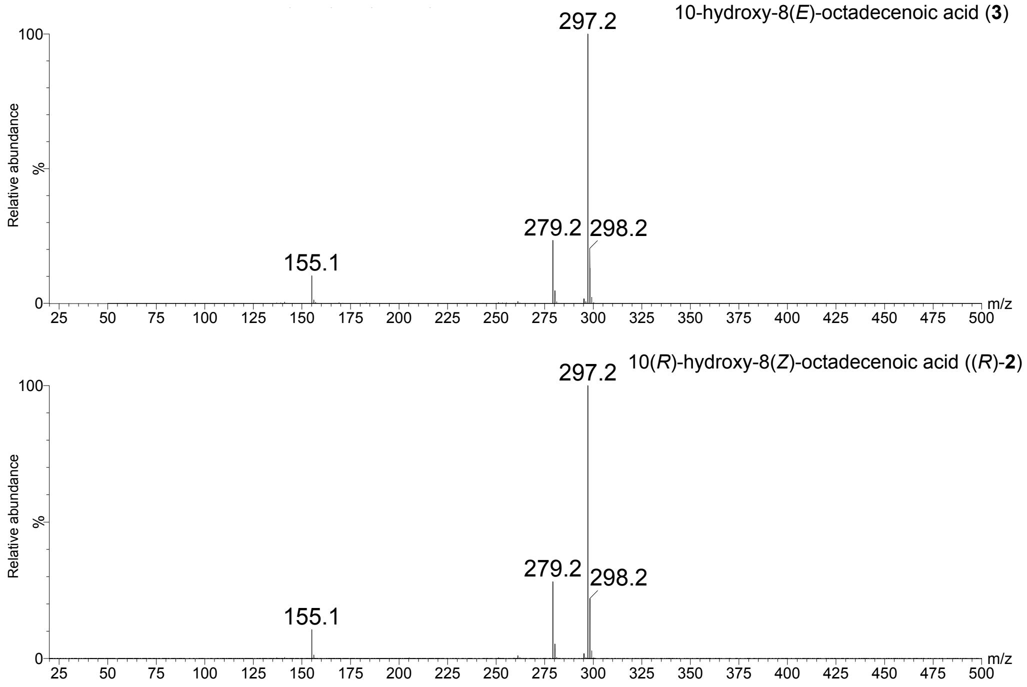
10-hydroxy-8(*E*)-octadecenoic acid (**3**)
297.2
279.2
298.2
155.1
Relative abundance
%
100
0
m/z
25 50 75 100 125 150 175 200 225 250 275 300 325 350 375 400 425 450 475 500
10(*R*)-hydroxy-8(*Z*)-octadecenoic acid ((*R*)-**2**)
297.2
279.2
298.2
155.1
Relative abundance
%
100
0
m/z
25 50 75 100 125 150 175 200 225 250 275 300 325 350 375 400 425 450 475 500

b

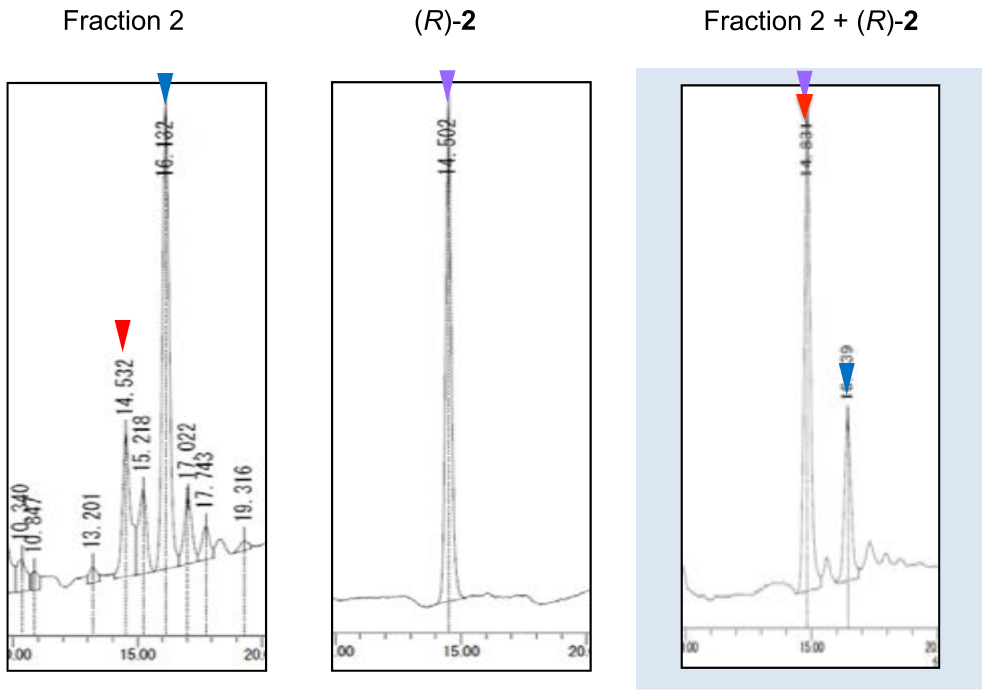
Fraction 2
(*R*)-**2**
Fraction 2 + (*R*)-**2**
16.132
14.532
15.218
17.022
17.743
19.316
13.201
10.340
10.847
14.502
15.00

c

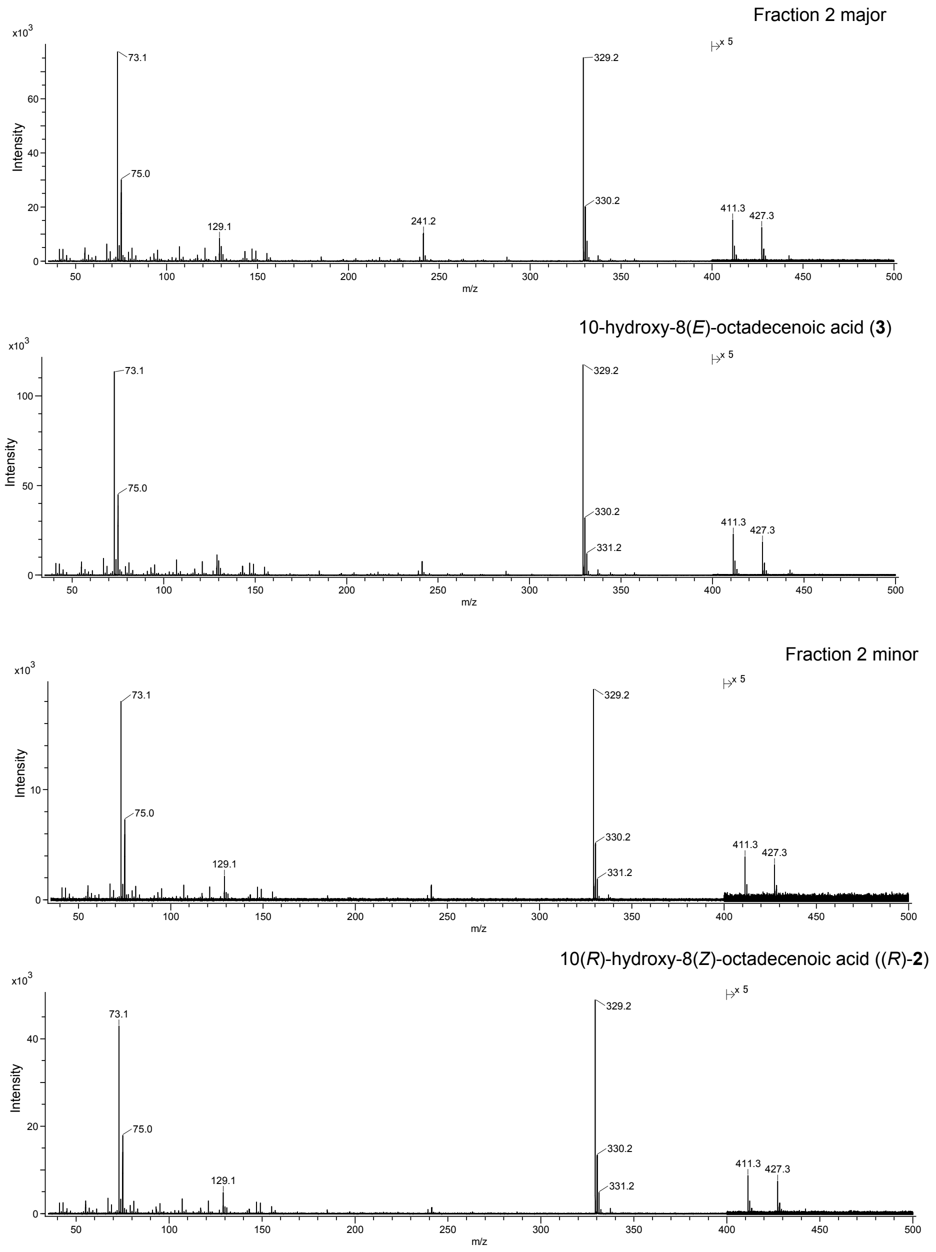

Figure S5. Confirmation of the existence of 10-hydroxy-8(*Z*)-octadecenoic acid (**2**) in fraction 2.

(**a**) MS/MS spectra of 10-hydroxy-8(*E*)-octadecenoic acid (**3**) and 10(*R*)-hydroxy-8(*Z*)-octadecenoic acid ((*R*)-**2**) obtained by hydrolysis of (*R*)-**1**. (**b**) Comparison of the HPLC chromatograms of fraction 2 and (*R*)-**2**. HPLC chromatogram of co-injection of fraction 2 and (*R*)-**2** was shown in the right panel. The peak of (*R*)-**2** (purple arrowhead) corresponds to the peak at T_R 14.5 of fraction 2 (red arrowhead). The peak indicated with a blue arrowhead (fraction 2) corresponds to 10-hydroxy-8(*E*)-octadecenoic acid (**3**). (**c**) MS spectra of the major peak of the isolated fraction 2, minor peak of the isolated fraction 2, peak at T_R 11.19 of **3**, and peak of T_R 11.19 of (*R*)-**2**. See **Figure 2c**.

a

Methyl 10(*R*)-hydroxy-8(*Z*)-octadecenoate (A)

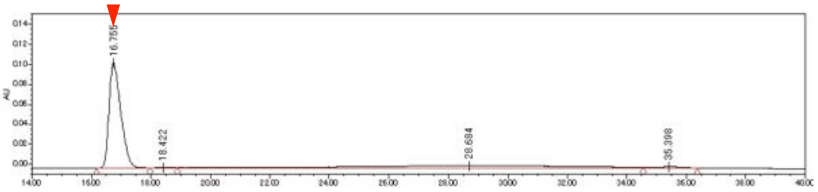

b

Methyl 10(*S*)-hydroxy-8(*Z*)-octadecenoate (B)

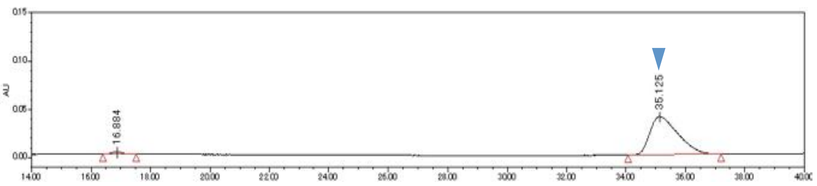

c

Racemic methyl 10-hydroxy-8(*E*)-octadecenoate (C)

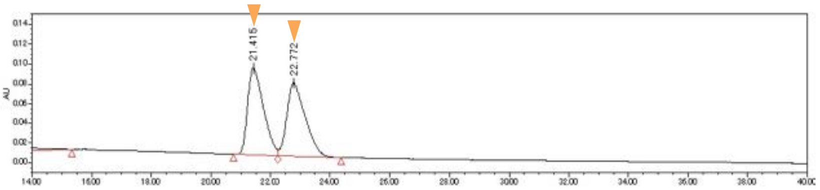

d

Methylated isolated fraction 2 (D)

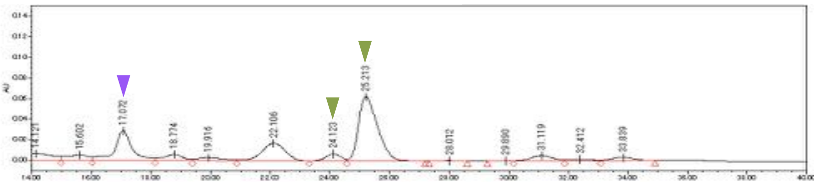

e

Co-injection of A and D

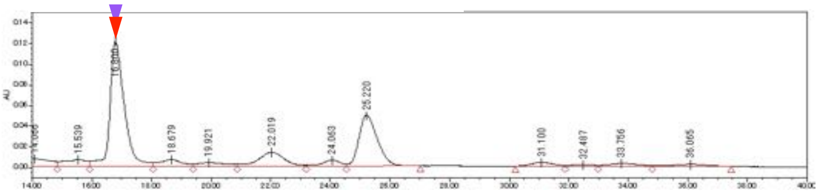

f

Co-injection of B and D

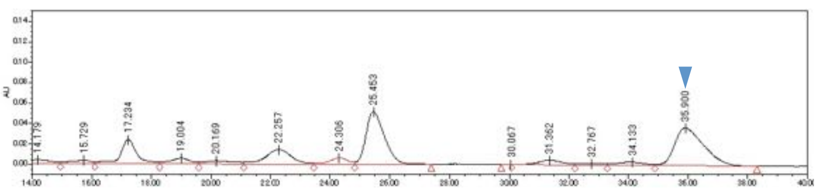

g

Co-injection of C and D

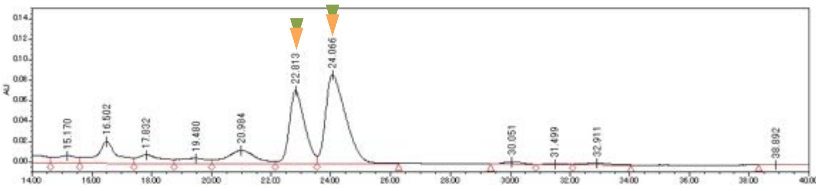

Figure S6. Identification of the absolute configuration at C-10 of 10-hydroxy-8(*Z*)-octadecenoic acid (**2**) in fraction 2.

(**a–d**) Synthetic 10(*R*)-hydroxy-8(*Z*)-octadecenoic acid ((*R*)-**2**) (A), 10(*S*)-hydroxy-8(*Z*)-octadecenoic acid ((*S*)-**2**) (B), racemic10-hydroxy-8(*E*)-octadecenoic acid (**3**) (C), and isolated fraction 2 (D) were methylated and subjected to HPLC using a chiral column. The red arrowhead indicates the methylated ester of 10(*R*)-hydroxy-8(*Z*)-octadecenoic acid ((*R*)-**2**), the blue arrowhead indicates the methylated ester of 10(*S*)-hydroxy-8(*Z*)-octadecenoic acid ((*S*)-**2**), and the yellow arrowheads indicate the *R* and *S* isomers of the methylated ester of 10-hydroxy-8(*E*)-octadecenoic acid (**3**). (**e–g**) HPLC chromatograms of co-injection of A and D (**e**), B and D (**f**), or C and D (**g**). The purple arrowhead in (**d**) corresponds to the red arrowhead in (a), indicating that fraction 2 contains (*R*)-**2** (**e**). The two green arrowheads in (**d**) correspond to the two yellow arrowheads in (**c**), indicating that fraction 2 also contains the *R* and *S* isomers of 10-hydroxy-8(*E*)-octadecenoic acid (**3**) (**g**).

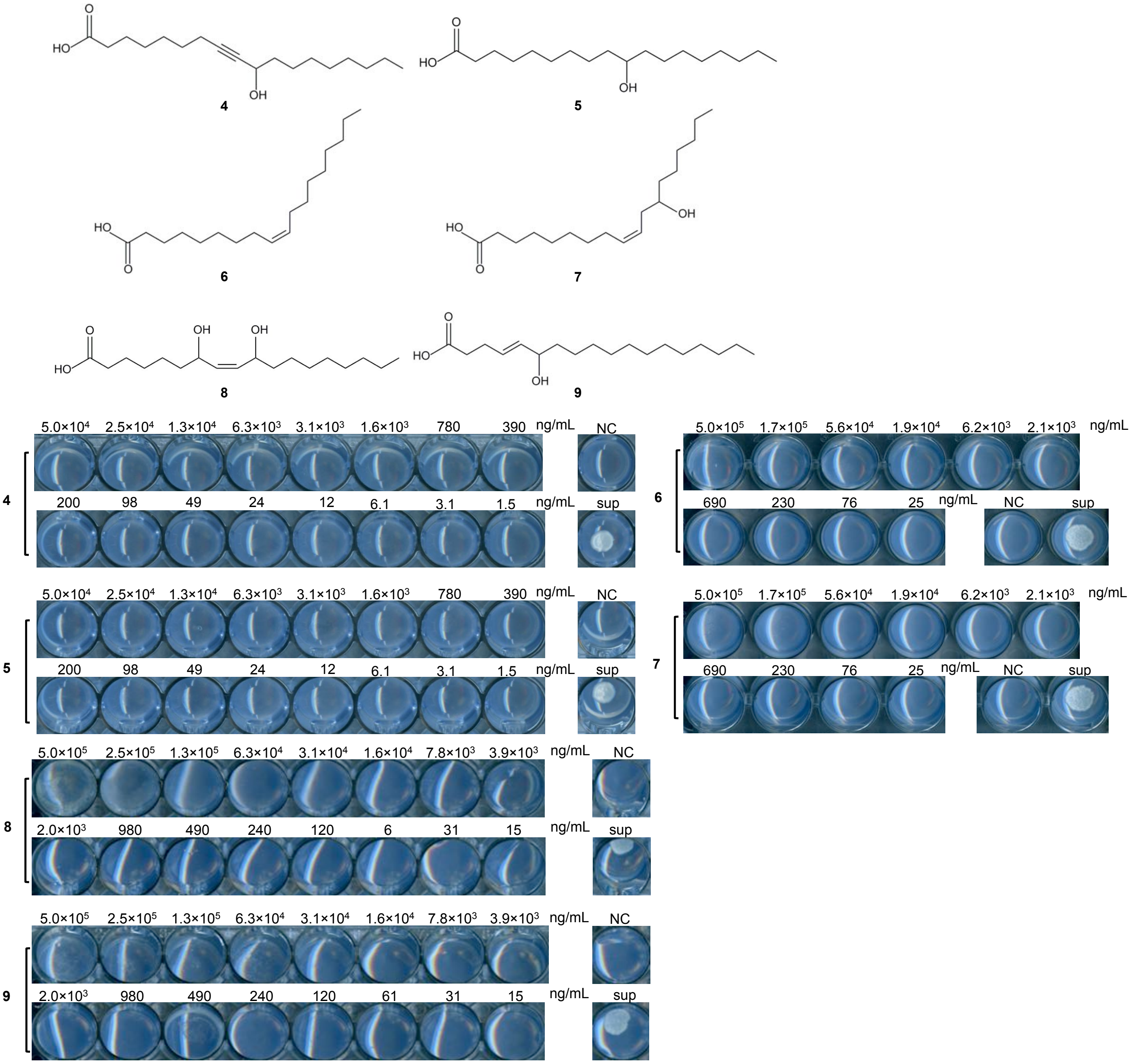

Figure S7. Examination of activities of structurally related compounds.

Six compounds (**4**–**9**) were dissolved in 50% MeOH. A 2-fold or 3-fold dilution series of each compound was prepared for examination of their activities by spot assay. *eca39Δ* cells (SpHT257) suspended in water at OD_{600} = 0.4 were spotted onto EMM [Glu]+ILV+AU layered with each diluted compound. Prototrophic cell supernatant (sup) and solvent (50% MeOH) were used as the positive and negative control (NC), respectively. Plates were incubated for 8 days or 10 days. A representative result is shown; the experiment was performed twice. **4**: 10-hydroxy-8-octadecynoic acid, **5**: 10-hydroxy-octadecanoic acid, **6**: oleic acid (9(*Z*)-octadecenoic acid), **7**: ricinoleic acid (12-hydroxy-9(*Z*)-octadecenoic acid), **8**: 7,10-dihydroxy-8(*Z*)octadecenoic acid, **9**: 6-hydroxy-4(*E*)-octadecenoic acid.

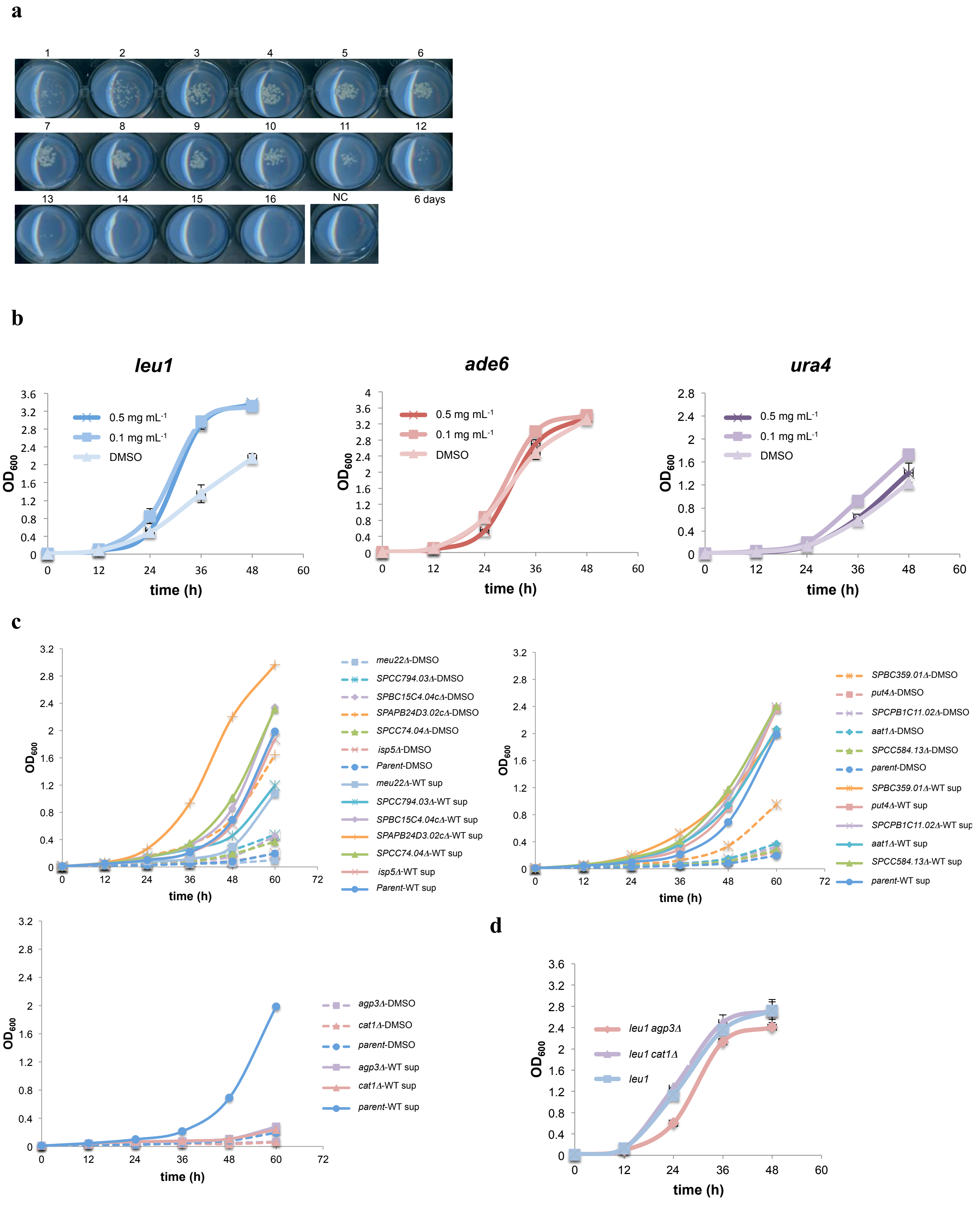

Figure S8. The Leu auxotrophic mutant (*leu1*) also exhibits adaptive growth.

(**a**) Supernatant of prototrophic cell culture (SpHT219) was extracted with EtOAc, and a 2-fold dilution series (wells 1–16) was prepared for examination of activity. Those dilutions were layered onto EMM

[374-N] supplemented with Ade, Ura, and Leu. *leu1* mutant cells (SpHT81) were suspended in water at OD_{600} = 0.02 and spotted onto the solid media. Solvent alone (50% MeOH) was used as a negative control (NC). Plates were incubated for 6 days. (**b**) Growth of the *leu1* mutant (SpHT81), *ade6* mutant (SpHT224), and *ura4* mutant (SpHT388) in EMM with excess NH_4Cl. Cells were cultured at 30°C for 48 hours in EMM containing 187 mM NH_4Cl (EMM [187-N]) supplemented with Ade, Ura, and Leu. Growth was monitored by measuring OD_{600} in the presence of DMSO or 0.1 or 0.5 mg/mL supernatant of prototrophic cell culture (SpHT219). The result is based on three cultures of each strain. (**c**) Screening of amino acid transporter genes involved in adaptive growth. Amino acid transporter gene mutants (SpHT478-489, 502) and their parental strain (SpHT227) were cultured in EMM containing 187 mM NH_4Cl (EMM [187-N]) supplemented with Ade, Ura, and Leu at 30°C for 60 hours. Growth was monitored by measuring OD_{600} every 12 hours in the presence of DMSO (dashed line) or 0.1 mg mL^{-1} of the supernatant of the prototrophic cell culture (SpHT219) (sup, solid line). The experiment was performed twice, and a representative result is shown. (**d**) Growth of the *leu1* mutant (SpHT81), the *leu1 agp3Δ* double mutant (XY-21), and the *leu1 cat1Δ* double mutant (XY-23) in general EMM. The experiment was performed three times.

a

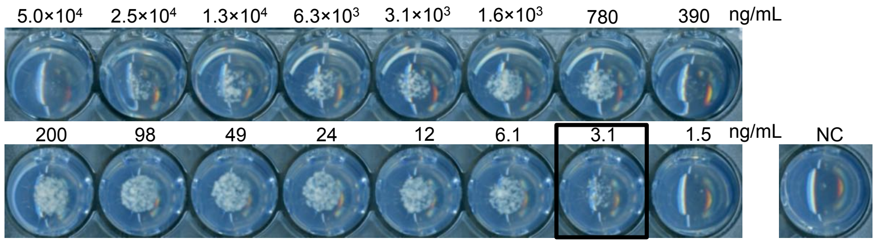

b

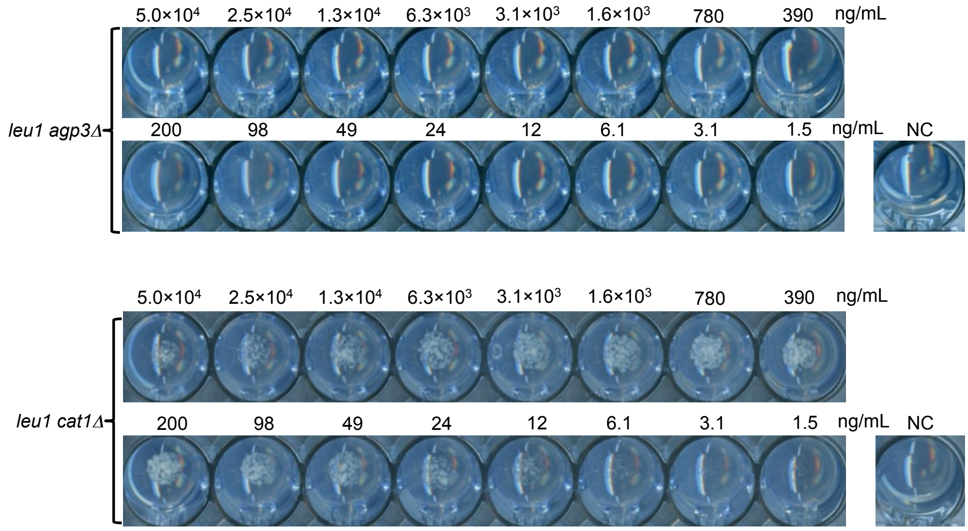

Figure S9. Adaptive growth of the Leu auxotrophic mutant is dependent on Agp3.

(**a**) Activity of isolated 10(*R*)-acetoxy-8(*Z*)-octadecenoic acid ((*R*)-**1**) on the *leu1* mutant. (*R*)-**1** was dissolved in 50% MeOH, and a 2-fold dilution series of the compound was prepared with a starting concentration of 5.0×10^4 ng/mL to examine its activity. Solvent (50% MeOH) was used as a negative control (NC). Dilutions and solvent were layered onto solid minimal media containing 374 mM NH_4Cl (EMM [374-N]) supplemented with Leu. *leu1* mutant cells (SpHT81) suspended in water at OD_{600} = 0.02 were spotted onto the solid media, and plates were incubated at 30°C for 5 days. (**b**) Activity of isolated 10(*R*)-acetoxy-8(*Z*)-octadecenoic acid ((*R*)-**1**) on the *leu1 agp3Δ* mutant (XY-21) and the *leu1 cat1Δ* mutant (XY-23). The experiment was prepared as described in (**a**).

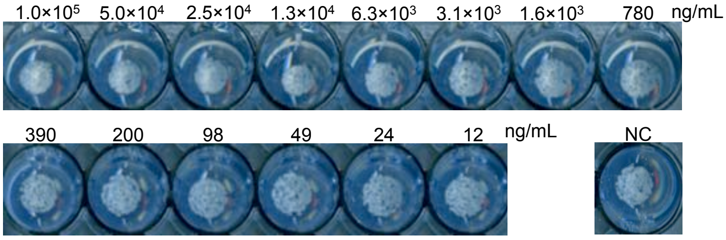

Figure S10. 10(*R*)-acetoxy-8(*Z*)-octadecenoic acid ((*R*)-**1**) does not inhibit growth of the prototrophic strain. Prototrophic cells (SpHT219) were suspended in water at OD_{600} = 0.01 and spotted onto general EMM media containing isolated (*R*)-**1** at a starting concentration of 1.0×10^5 ng/mL. The plate was incubated for 3 days.

Table S1. ^{1}H and ^{13}C NMR data for 10-acetoxy-8(*Z*)-octadecenoic acid (**1**).

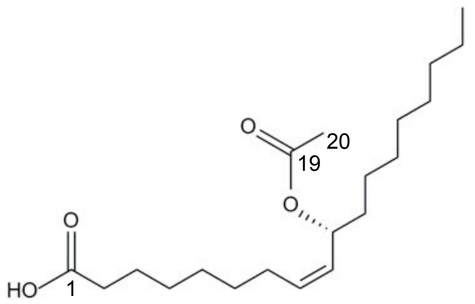

Position	δ_H, (*J* in Hz)	δ_C
1	-	177.8
2	2.27, t (7.4)	35.0
3	1.59, m	26.2
4, 5	1.32–1.34	30.0–30.6
6	1.35, m	30.3
7	2.15, m	28.8
8	5.53, m	134.9
9	5.29, brt (10.5)	129.5
10	5.53, m	71.6
11	1.49, m; 1.65, m	35.8
12–15	1.32–1.34	30.0–30.6
16	1.29, m	33.0
17	1.3, m	23.7
18	0.91, t (6.9)	14.4
19	-	172.2
20	1.99, s	21.2

Table S2. ^{1}H and ^{13}C NMR data for 10-hydroxy-8(*E*)-octadecenoic acid (**3**).

Position	δ_H, (*J* in Hz)	δ_C
1	-	180.5
2	2.21, t (7.4)	37.0
3	1.59, m	26.9
4, 5	1.30–1.35	30.3–30.7
6	1.35, m	30.3
7	2.04, m	33.2
8	5.59, dt (15.3, 6.8)	132.5
9	5.40, brdd (15.3, 7.1)	134.5
10	3.94, m	73.8
11	1.52, m; 1.43, m	38.5
12–15	1.30–1.35	30.3–30.7
16	1.30, m	33.0
17	1.31, m	23.7
18	0.91, t (6.9)	14.4

Table S3. Strains used in this study.

Name	Genotype	Source
Schizosaccharomyces pombe		
SpHT81	*h⁻ leu1-32*	Ref. 3
SpHT219	h^-	Ref. 3
SpHT224	h^- *ade6-M216*	Ref. 3
SpHT227[a]	h^+ *ade6-M216 ura4-D18 leu1-32*	Ref. 3
SpHT257[a]	h^- *eca39Δ::kanMX4*	Ref. 3
SpHT388	h^- *ura4-D18*	Ref. 3
XY-21[a]	h^- *leu1-32 agp3Δ::kanMX4*	This study
XY-23[a]	h^- *leu1-32 cat1Δ::kanMX4*	This study
SpHT478 [a]	h^+ *ade6-M21? ura4-D18 leu1-32 meu22Δ::kanMX4*	Ref. 3
SpHT479[a]	h^+ *ade6-M21? ura4-D18 leu1-32 spbc15c4.04cΔ::kanMX4*	Ref. 3
SpHT480[a]	h^+ *ade6-M21? ura4-D18 leu1-32 spapb24d3.02cΔ::kanMX4*	Ref. 3
SpHT481[a]	h^+ *ade6-M21? ura4-D18 leu1-32 spcc74.04Δ::kanMX4*	Ref. 3
SpHT482[a]	h^+ *ade6-M21? ura4-D18 leu1-32 isp5Δ::kanMX4*	Ref. 3
SpHT483[a]	h^+ *ade6-M21? ura4-D18 leu1-32 spbc359.01Δ::kanMX4*	Ref. 3
SpHT484[a]	h^+ *ade6-M21? ura4-D18 leu1-32 put4Δ::kanMX4*	Ref. 3
SpHT485[a]	h^+ *ade6-M21? ura4-D18 leu1-32 spcpb1c11.02Δ::kanMX4*	Ref. 3
SpHT486[a]	h^+ *ade6-M21? ura4-D18 leu1-32 agp3Δ::kanMX4*	Ref. 3
SpHT487[a]	h^+ *ade6-M21? ura4-D18 leu1-32 aat1Δ::kanMX4*	Ref. 3
SpHT488[a]	h^+ *ade6-M21? ura4-D18 leu1-32 cat1Δ::kanMX4*	Ref. 3
SpHT489[a]	h^+ *ade6-M21? ura4-D18 leu1-32 spcc584.13Δ::kanMX4*	Ref. 3
SpHT502[a]	h^+ *ade6-M21? ura4-D18 leu1-32 spcc794.03Δ::kanMX4*	Ref. 3
Saccharomyces cerevisiae		
YHT842	*MAT***a**	Ref. 3

[a]These mutants utilize the knock-out alleles described in Reference 14 and/or their derivatives.

Supplementary Note

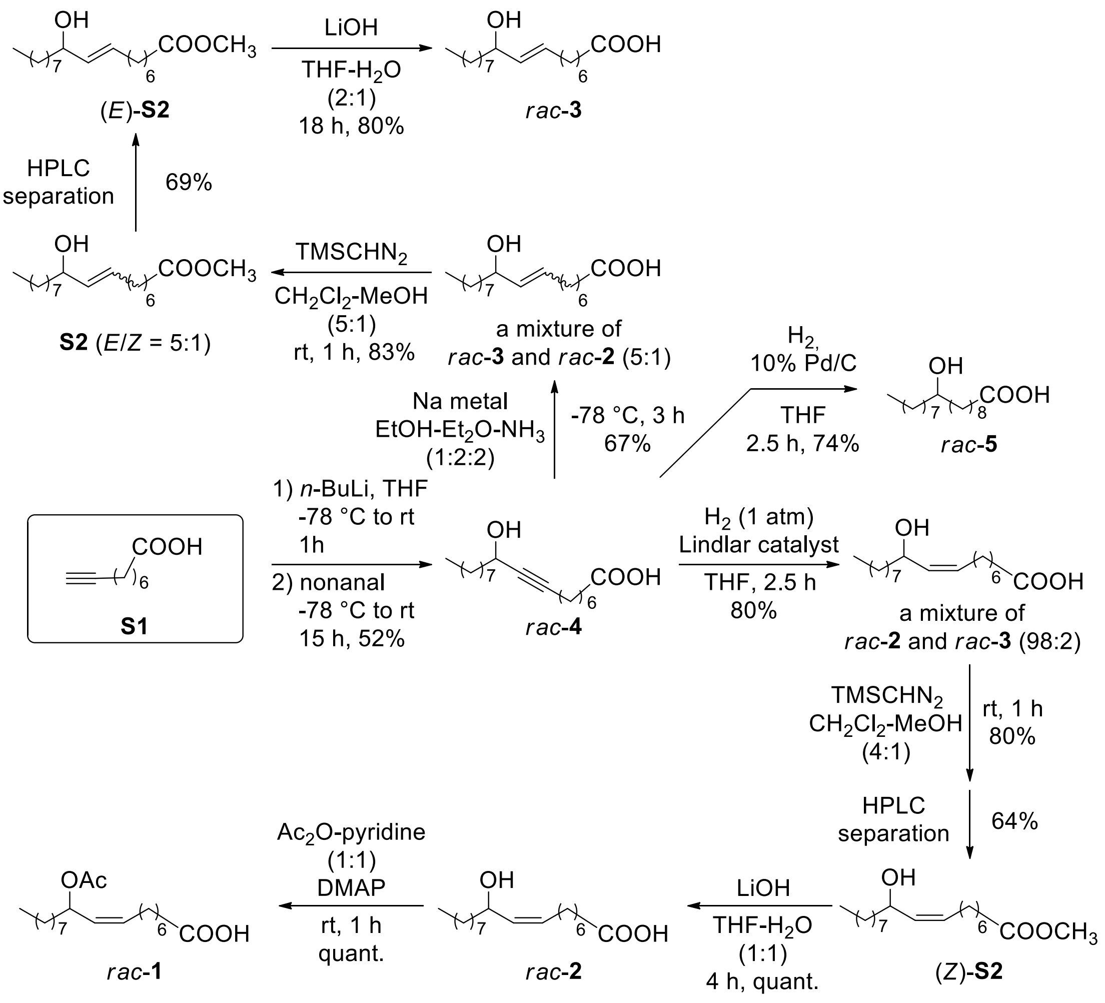

Scheme 1. Synthesis of racemic 10-hydroxyoctadec-8-enoic acid derivatives

10-hydroxyoctadec-8-ynoic acid (*rac*-**4**)

A solution of **S1** (163.5 mg, 1.06 mmol) in dry THF (11 mL) under Ar atmosphere was cooled to −78 °C. *n*-BuLi (1.5 mL of a 1.55 M hexane solution, 2.33 mmol) was added dropwise via syringe. The resulting mixture was stirred at −78 °C for 1 h, and then warmed to room temperature, and stirred for another 1 h. The mixture was cooled to −78 °C again, and nonanal (0.2 mL, 1.17 mmol) was added slowly via syringe. The mixture was stirred at −78 °C for 1 h before being allowed to warm to room temperature, and then stirred for 15 h. The reaction was quenched by addition of a saturated aqueous solution of $NaHCO_3$. The resulting mixture was extracted thrice with Et_2O. The combined organic layers were dried over Na_2SO_4 and concentrated *in vacuo*. The residue was purified by silica-gel flash column chromatography (Hexane/EtOAc 5:1) to give *rac*-**4** (164.7 mg, 52%) as a colorless oil.

^{1}H NMR ($CDCl_3$, 400 MHz): δ 4.34 (tt, *J* = 6.4 Hz, 1.8 Hz, 1H), 2.36 (t, *J* = 7.4 Hz, 2H), 2.21 (td, *J* = 6.9 Hz, 1.8 Hz, 2H), 1.73-1.59 (m, 4H), 1.55-1.23 (m, 18H), 0.88 (t, *J* = 6.9 Hz, 3H).
^{13}C NMR ($CDCl_3$, 100 MHz): δ 178.7, 85.4, 81.6, 62.9, 38.3, 33.9, 32.0, 29.7, 29.44, 29.39, 28.6, 28.4 (2C), 25.4, 24.6, 22.8, 18.7, 14.3.
HRMS (ESI) calcd for $C_{18}H_{31}O_3$ $[M-H]^-$ 295.2273; found: 295.2270.

10-hydroxyoctadec-8-enoic acid (a mixture of *rac*-**3** and *rac*-**2**)

To a solution of *rac*-**4** (10 mg, 0.034 mmol) in EtOH (0.5 mL), Et_2O (1.0 mL) and NH_3 (approx. 1.0 mL) was added Na metal (a few pieces) until the color remained dark blue. After 1.5 h, the mixture was warmed to room temperature, and NH_3 was allowed to evaporate. The residue was neutralized with a 2 M aqueous solution of HCl until pH was 1-2, and then extracted thrice with Et_2O. The combined organic layers were dried over Na_2SO_4 and concentrated *in vacuo*. The residue was purified by silica-gel flash column chromatography (Hexane/EtOAc 5:1). ^{1}H NMR revealed the existence of *rac*-**4** (about 10%) in the product; therefore, the same reaction was conducted again to provide a mixture of *rac*-**3** and *rac*-**2** (5:1, 6.7 mg, 67%).

methyl (8*E*)-10-hydroxyoctadec-8-enoate ((*E*)-**S2**)

The obtained mixture of *rac*-**3** and *rac*-**2** (5:1, 6.7 mg, 0.022 mmol) was dissolved in a mixture of CH_2Cl_2 and MeOH (5:1, 180 μL) and treated with (trimethylsilyl)diazomethane (0.6 M in hexane, 123 μL, 0.074 mmol) under Ar atmosphere at room temperature. The mixture was allowed to react for 1 h at room temperature. After the solvent was evaporated, the residue was dissolved in H_2O and extracted twice with EtOAc. The combined organic layers were dried over Na_2SO_4 and concentrated *in vacuo*. The residue was purified by silica-gel flash column chromatography (Hexane/EtOAc 10:1) to give a mixture of (*E*)-**S2** and (*Z*)-**S2** (5:1, 5.8 mg, 83%) as a colorless oil.

Pure (*E*)-**S2** was obtained by HPLC separation (PEGASIL silica SP100 (250 mm × 100 mm), Hexane/EtOAc 6:1, 3 ml/min, 190 nm UV, retention time: *E*-isomer, 17.7 min).
^{1}H NMR ($CDCl_3$, 400 MHz): δ 5.61 (dtd, *J* = 15.2 Hz, 6.4 Hz, 0.9 Hz, 1H), 5.44 (ddt, *J* = 15.2 Hz, 6.9 Hz, 1.4 Hz, 1H), 4.03 (br ddd, *J* = 6.9 Hz, 6.9 Hz, 3.7 Hz, 1H), 3.67 (s, 3H), 2.30 (t, *J* = 7.4 Hz, 2H), 2.02 (dt, *J* = 6.9 Hz, 6.4 Hz, 2H), 1.65-1.25 (m, 22H), 0.88 (t, *J* = 6.9 Hz, 3H).
^{13}C NMR ($CDCl_3$, 100 MHz): δ 174.5, 133.3, 132.1, 73.4, 51.6, 37.5, 34.2, 32.2, 32.0, 29.7 (2C), 29.4, 29.1 (2C), 28.8, 25.6, 25.0, 22.8, 14.3.

HRMS (ESI) calcd for $C_{19}H_{36}O_3Na$ $[M+Na]^+$ 335.2562; found: 335.2563.

(8*E*)-10-hydroxyoctadec-8-enoic acid (*rac*-**3**)

To a solution of (*E*)-**S2** (3 mg, 0.0096 mmol) in a mixture of THF and H_2O (2:1, 600 μL), LiOH·H_2O (2.4 mg, 0.058 mmol) was added as a powder. The resulting mixture was stirred at room temperature for 18 h. After that, H_2O (10 mL) was added at 0 °C, and the solution was acidified by addition of a 2 M aqueous solution of HCl until pH was 2-3. The resulting mixture was extracted thrice with EtOAc. The combined organic layers were dried over Na_2SO_4 and concentrated *in vacuo*. The residue was purified by silica-gel flash column chromatography (Hexane/EtOAc 5:1) to give pure *rac*-**3** (2.3 mg, 80%) as a white solid.

^{1}H NMR ($CDCl_3$, 400 MHz): δ 5.61 (dtd, *J* = 15.2 Hz, 6.4 Hz, 0.9 Hz, 1H), 5.44 (ddt, *J* = 15.2 Hz, 6.9 Hz, 1.4 Hz, 1H), 4.03 (dt, *J* = 6.9 Hz, 6.4 Hz, 1H), 2.34 (t, *J* = 7.4 Hz, 2H), 2.02 (td, *J* = 6.9 Hz, 6.4 Hz, 2H), 1.64 (m 2H), 1.57-1.22 (m, 20H), 0.88 (t, *J* = 6.4 Hz, 3H).

^{13}C NMR ($CDCl_3$, 100 MHz): δ 178.9, 133.3, 132.2, 73.4, 37.4, 34.0, 32.2, 32.0, 29.9, 29.7, 29.4, 29.0, 28.9, 28.7, 25.6, 24.7, 22.8, 14.3.

HRMS (ESI) calcd for $C_{18}H_{33}O_3$ $[M-H]^-$ 297.2430; found: 297.2430.

(8*Z*)-10-hydroxyoctadec-8-enoic acid (*rac*-**2**)

Compound *rac*-**4** (8.0 mg, 0.027 mmol) and Lindlar catalyst (Aldrich, no. 20573-7, 1 mg) were mixed in THF (1 mL). The resulting suspension was stirred under H_2 atmosphere at room temperature for 2.5 h. The catalyst was removed by filtration through a short Celite® pad, and the filtrate was concentrated *in vacuo* to give crude *rac*-**2** (6.4 mg, 80%) as a colorless oil contaminated with *rac*-**3** (approx. 2%). The crude material was converted to their methyl ester derivatives, which were then separated by HPLC to give pure compound (*Z*)-**S2**. The methyl group of (*Z*)-**S2** was removed in a similar manner as in the synthesis of *rac*-**3** to generate pure *rac*-**2**.

(*Z*)-**S2:** ^{1}H NMR ($CDCl_3$, 400 MHz): δ 5.46 (dt, *J* = 11.0 Hz, 7.4 Hz, 1H), 5.36 (ddt, *J* = 11.0 Hz, 8.7 Hz, 1.4 Hz, 1H), 4.41 (br dt, J = 8.7 Hz, 6.4 Hz, 1H), 3.66 (s, 3H), 2.30 (t, *J* = 7.4 Hz, 2H), 2.08 (m, 2H), 1.65-1.54 (m, 3H), 1.46-1.21 (m, 20H), 0.88 (t, *J* = 6.4 Hz, 3H).

^{13}C NMR ($CDCl_3$, 100 MHz): δ 174.5, 132.9, 132.2, 67.9, 51.6, 37.8, 34.2, 32.0, 29.8, 29.7, 29.6, 29.4, 29.1, 29.0, 27.7, 25.5, 25.0, 22.8, 14.3.

HRMS (ESI) calcd for $C_{19}H_{36}O_3Na$ $[M+Na]^+$ 335.2562; found: 335.2562.

rac-**2:** ^{1}H NMR ($CDCl_3$, 400 MHz): δ 5.46 (dt, *J* = 11.0 Hz, 7.4 Hz, 1H), 5.36 (ddt, *J* =

11.0 Hz, 8.7 Hz, 1.4 Hz, 1H), 4.42 (br dt, J = 8.7 Hz, 6.4 Hz, 1H), 2.34 (t, *J* = 7.4 Hz, 2H), 2.09 (m, 2H), 1.67-1.57 (m, 3H), 1.46-1.19 (m, 18H), 0.88 (t, *J* = 6.4 Hz, 3H).
^{13}C NMR ($CDCl_3$, 100 MHz): δ 178.7, 132.8, 132.3, 67.9, 37.6, 33.9, 32.0, 29.8, 29.7, 29.5, 29.4, 28.9, 28.8, 27.6, 25.5, 24.7, 22.8, 14.3.
HRMS (ESI) calcd for $C_{18}H_{33}O_3$ $[M-H]^-$ 297.2430; found: 297.2429.

(8*Z*)-10-acetoxyoctadec-8-enoic acid (*rac*-**1**)

To a solution of *rac*-**2** (1.0 mg, 3.35 μmol) in a mixture of Ac_2O and pyridine (1:1, 100 μL) was added 4-dimethylaminopyridine (DMAP, 0.2 mg, 0.00188 mmol) at 0 °C. The resulting mixture was stirred at room temperature for 1 h. H_2O (10 mL) was added at 0 °C, and the solution was acidified by addition of 2 M aqueous solution of HCl until the pH was 2-3. The resulting mixture was extracted thrice with EtOAc. The combined organic layers were dried over Na_2SO_4 and concentrated *in vacuo*. The residue was purified by silica-gel flash column chromatography (Hexane/EtOAc 5:1) to give *rac*-**1** (1.2 mg, quant.) as a colorless oil.
^1H NMR ($CDCl_3$, 400 MHz): δ 5.54-5.48 (m, 2H), 5.29 (ddt, *J* = 10.6 Hz, 9.7 Hz, 1.4 Hz, 1H), 2.34 (t, *J* = 7.4 Hz, 2H), 2.20-2.06 (m, 2H), 2.02 (s, 3H), 1.65-1.20 (m, 22H), 0.88 (t, *J* = 6.9 Hz, 3H).
^{13}C NMR ($CDCl_3$, 100 MHz): δ 177.9, 170.7, 134.0, 128.4, 70.7, 34.9, 33.8, 32.0, 29.64, 29.59, 29.4 (2C), 29.0, 28.9, 27.9, 25.2, 24.7, 22.8, 21.5, 14.3.
HRMS (ESI) calcd for $C_{20}H_{35}O_4$ $[M-H]^-$ 339.2535; found: 339.2530.

10-hydroxyoctadecanoic acid (*rac*-**5**)

Compound *rac*-**4** (8.6 mg, 0.029 mmol) and 10 wt% Pd on carbon (Wako, no. 163-15272, 0.9 mg) were mixed in THF (1 mL). The resulting suspension was stirred under H_2 atmosphere at room temperature for 2.5 h. The solid was removed by filtration through a short Celite® pad, and the filtrate was concentrated *in vacuo*. The residue was purified by silica-gel flash column chromatography (Hexane/EtOAc 5:1) to give *rac*-**5** (6.5 mg, 74%) as a colorless oil.
^1H NMR ($CDCl_3$, 400 MHz): δ 3.58 (m, 1H), 2.34 (t, *J* = 7.4 Hz, 2H), 1.63 (m, 2H), 1.46-1.28 (m, 26H), 0.88 (t, *J* = 6.4 Hz, 3H).
^{13}C NMR ($CDCl_3$, 100 MHz): δ 179.0, 72.2, 37.6, 37.5, 34.0, 32.0, 29.9, 29.73, 29.70, 29.5, 29.4, 29.3, 29.1, 25.8, 25.7, 24.8, 22.8, 14.3.
HRMS (ESI) calcd for $C_{18}H_{35}O_3$ $[M-H]^-$ 299.2586; found: 299.2588.

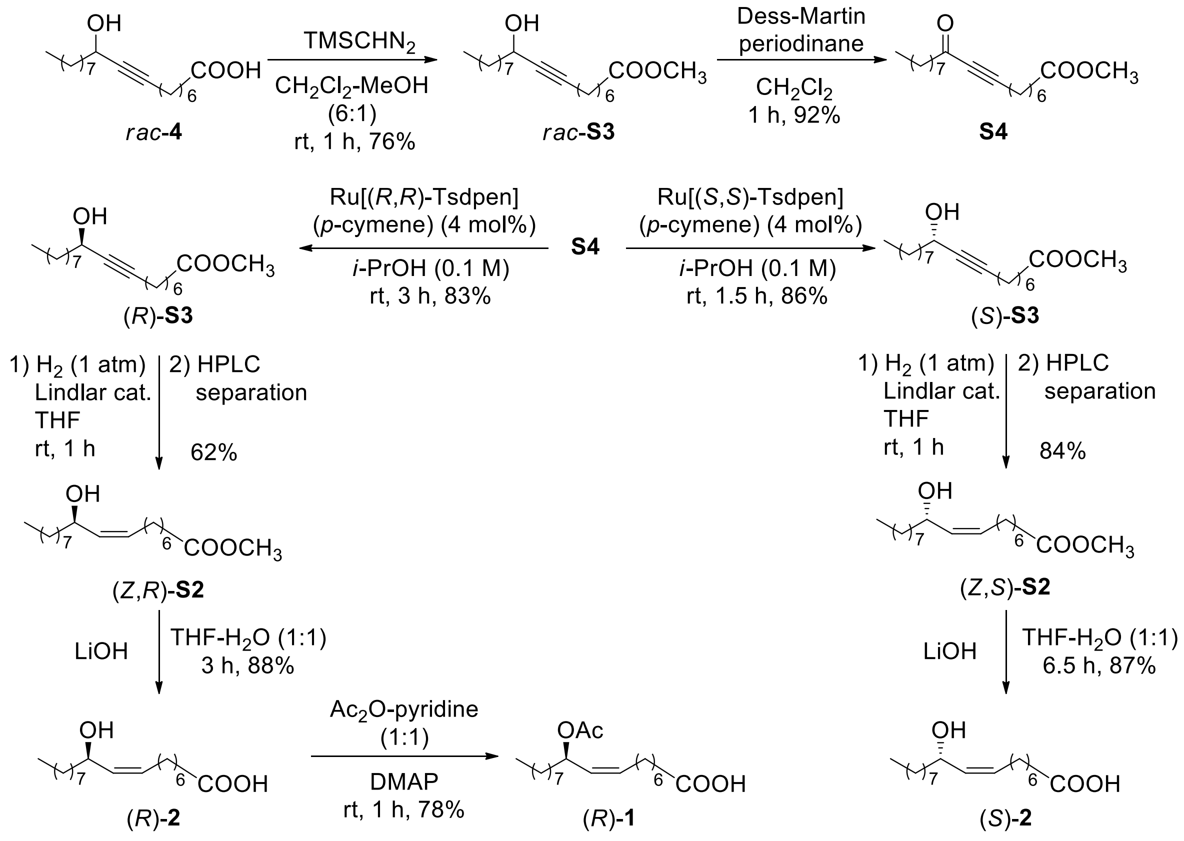

Scheme 2. Synthesis of optical pure (8*Z*)-10-hydroxyoctadec-8-enoic acid derivatives

methyl 10-hydroxyoctadec-8-ynoate (*rac*-**S3**)

Compound *rac*-**4** (43 mg, 0.145 mmol) was dissolved in a mixture of CH_2Cl_2 and MeOH (6:1, 700 μL) and treated with (trimethylsilyl)diazomethane (0.6 M in hexane, 800 μL, 0.479 mmol) under Ar atmosphere at room temperature. The mixture was allowed to react for 1 h at room temperature. After the solvent was evaporated, the residue was dissolved in H_2O and extracted twice with EtOAc. The combined organic layers were dried over Na_2SO_4 and concentrated *in vacuo*. The residue was purified by silica-gel flash column chromatography (hexane/EtOAc 10:1) to give *rac*-**S3** (34.4 mg, 76%) as a colorless oil.

^{1}H NMR ($CDCl_3$, 400 MHz): δ 4.34 (m, 1H), 3.67 (s, 3H), 2.31 (t, J = 7.4 Hz, 2H), 2.20 (td, J = 6.9 Hz, 1.8 Hz, 2H), 1.76 (d, J = 5.5 Hz, 1H), 1.73-1.21 (m, 22H), 0.88 (t, J = 6.9 Hz, 3H).

^{13}C NMR ($CDCl_3$, 100 MHz): δ 174.4, 85.3, 81.7, 62.8, 51.6, 38.3, 34.1, 32.0, 29.6, 29.42, 29.36, 28.7, 28.5, 25.3, 24.9, 22.8 (2C), 18.7, 14.2.

HRMS (ESI) calcd for $C_{19}H_{34}O_3Na$ $[M+Na]^+$ 333.2406; found: 333.2403.

methyl 10-oxooctadec-8-ynoate (S4)

To a solution of compound *rac*-**S3** (33 mg, 0.106 mmol) in CH_2Cl_2 (1 mL) was added Dess–Martin periodinane (135 mg, 0.319 mmol) at room temperature. After stirring for 1 h, the mixture was diluted with Et_2O, poured into a saturated aqueous solution of $Na_2S_2O_3$ at 0 °C. The resulting mixture was extracted thrice with Et_2O. The combined organic layers were washed with a saturated aqueous solution of $NaHCO_3$, dried over Na_2SO_4, and concentrated *in vacuo*. The residue was purified by silica-gel flash column chromatography (Hexane/EtOAc 40:1) to give **S4** (30.2 mg, 92%) as a colorless oil.

^{1}H NMR ($CDCl_3$, 400 MHz): δ 3.65 (s, 3H), 2.50 (t, *J* = 7.4 Hz, 2H), 2.34 (t, *J* = 6.9 Hz, 2H), 2.30 (t, *J* = 7.4 Hz, 2H), 1.67-1.53 (m, 6H), 1.45-1.19 (m, 14H), 0.86 (t, *J* = 6.9 Hz, 3H).

^{13}C NMR ($CDCl_3$, 100 MHz): *δ* 188.7, 174.2, 94.0, 81.1, 51.6, 45.7, 34.1, 31.9, 29.4, 29.2, 29.1, 28.62, 28.58, 27.6, 24.8, 24.3, 22.8, 19.0, 14.2.

HRMS (ESI) calcd for $C_{19}H_{32}O_3Na$ $[M+Na]^+$ 331.2249; found: 331.2249.

methyl (10*S*)-10-hydroxyoctadec-8-ynoate ((*S*)-**S3**)

Anhydrous isopropanol was deoxygenated by three freeze-thaw cycles. Compound **S4** (6.7 mg, 0.0217 mmol) was dissolved in the deoxygenated isopropanol (110 μL), to which was added the Noyori hydrogen transfer catalyst Ru[(*S*,*S*)-Tsdpen](*p*-cymene) (7.84 mM in isopropanol, 110 μL, 0.869 μmol, 4 mol%) at room temperature. The resulting solution was stirred at room temperature for 1.5 h, and then concentrated *in vacuo*. The residue was purified by silica-gel flash column chromatography (Hexane/EtOAc 20:1) to give (*S*)-**S3** (6.2 mg, 92%) as a colorless oil.

The ^{1}H and ^{13}C NMR spectra and HRMS data for (*S*)-**S3** were identical to those of the racemic alcohol *rac*-**S3**.

$[\alpha]^{24}_{D}$ = +0.98 (c = 0.35, $CHCl_3$).

methyl (10*R*)-10-hydroxyoctadec-8-ynoate ((*R*)-**S3**)

Compound (*R*)-**S3** was synthesized in a manner similar to the synthesis of compound (*S*)-**S3**, except being catalyzed by the Noyori hydrogen transfer catalyst Ru[(*R*,*R*)-Tsdpen](*p*-cymene).

The ^{1}H and ^{13}C NMR spectra and HRMS data for the (*R*)-**S3** were identical to those of the racemic alcohol *rac*-**S3**. The absolute configuration of the alcohol was determined by a modified Mösher's method (ref.: Ohtani, I.; Kusumi, T.; Kashman, Y.; Kakisawa, H. *J. Am. Chem. Soc.*, **1991**, *113*, 4092-4096.). The differences in the chemical shift values were obtained by subtracting the signal values for (*R*,*R*)-**S5** from those for the

(*R*,*S*)-**S5** ester ($\delta_\Delta = \delta_{(R,S)\text{-S5}} - \delta_{(R,R)\text{-S5}}$).

$[\alpha]^{25}_{D} = -1.28$ (c = 0.335, $CHCl_3$).

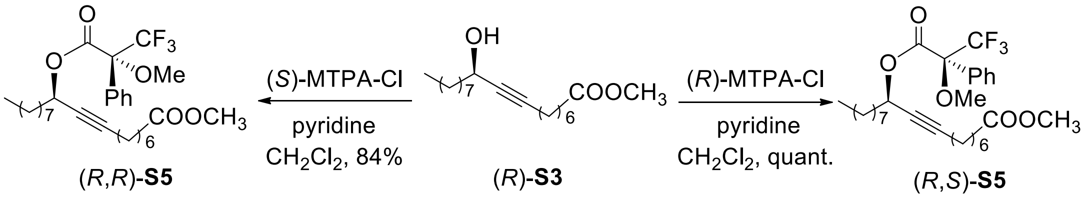

Difference in chemical shifts in (*R*,*S*)-**S5** and (*R*,*R*)-**S5**

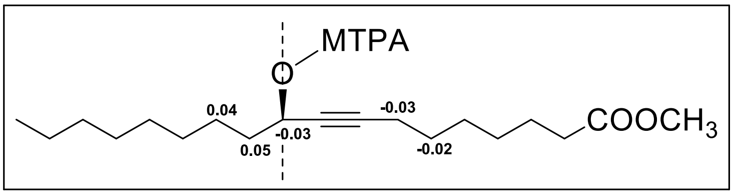

methyl (8*Z*,10*S*)-10-hydroxyoctadec-8-enoate ((*Z*,*S*)-**S2**)

Compound (*S*)-**S6** (4.5 mg, 0.0145 mmol) and Lindlar catalyst (Aldrich, no. 20573-7, 0.5 mg) were mixed in THF (800 μL). The resulting suspension was stirred under H_2 atmosphere at room temperature for 6.5 h. The catalyst was removed by filtration through a short Celite® pad, and the filtrate was concentrated *in vacuo* to give crude (*Z*,*S*)-**S2** contaminated with approx. 5% (*E*,*S*)-**S2**. Pure (*Z*,*S*)-**S2** (3.8 mg, 84%, 96% ee) was obtained by HPLC separation (PEGASIL silica SP100 (250 mm × 100 mm), hexane/EtOAc 6:1, 3 ml/min, 190 nm UV, retention time: 18 min).

$[\alpha]^{23}_{D} = -19.43$ (c = 0.09, $CHCl_3$)

The enantiomeric excess (ee) of the alcohol was determined by chiral HPLC analysis (Daicel Chiral AD-RH (250 mm × 46 mm), CH_3CN/MeOH 5:1 with 5% H_2O, 0.3 ml/min, 205 nm UV, retention time: 35.1 min).

The ^{1}H and ^{13}C NMR spectra and HRMS data for the (*Z*,*S*)-**S2** were identical to those of the racemic (*Z*)-**S2**.

methyl (8*Z*,10*R*)-10-hydroxyoctadec-8-enoate ((*Z*,*R*)-**S2**)

Compound (*Z*,*R*)-**S2** was synthesized in a manner similar to the synthesis of compound (*S*,*Z*)-**S2**.

$[\alpha]^{24}_{D} = +17.93$ (c = 0.1, $CHCl_3$)

The % ee of the alcohol was determined by chiral HPLC analysis (Daicel chiral AD-RH (250 mm × 46 mm), CH_3CN/MeOH 5:1:5% H_2O, 0.3 ml/min, 205 nm, retention time: 16.8 min).

The ^{1}H and ^{13}C NMR spectra and HRMS data for the (*Z*,*R*)-**S2** were identical to those

of the racemic (*Z*)-**S2**.

The absolute configuration of the alcohol was determined by a modified Mösher's method. The differences in the chemical shift values were obtained by subtracting the signal values for the (*Z*,*R*,*R*)-**S6** from those for the (*Z*,*R*,*S*)-**S6** ester ($\delta_\Delta = \delta_{(Z,R,S)\text{-}\mathbf{S6}} - \delta_{(Z,R,R)\text{-}\mathbf{S6}}$).

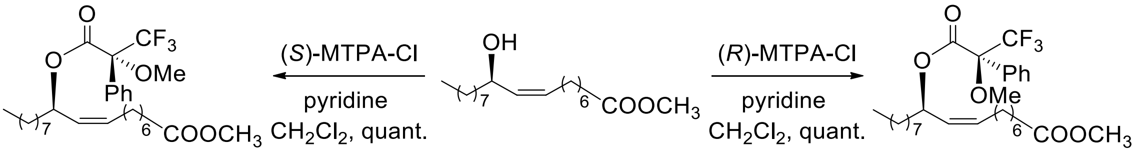

(*Z*,*R*,*R*)-**S6** (*Z*,*R*)-**S2** (*Z*,*R*,*S*)-**S6**

Difference in chemical shifts in (*Z*,*R*,*S*)-**S6** and (*Z*,*R*,*R*)-**S6**

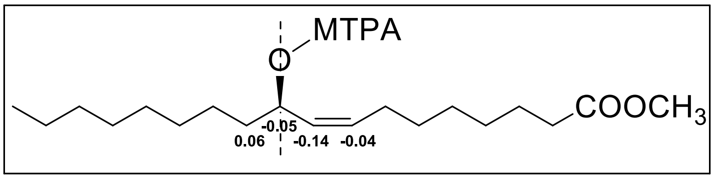

(8*Z*,10*S*)-10-hydroxyoctadec-8-enoic acid ((*S*)-**2**)

Compound (*S*)-**2** was synthesized from (Z,*S*)-**S2** in a manner similar to the synthesis of *rac*-**3**. The ^{1}H and ^{13}C NMR spectra and HRMS data for the (*S*)-**2** were identical to those of the *rac*-**2**.

$[\alpha]^{24}_{D} = -21.6$ (c = 0.055, $CHCl_3$)

(8*Z*,10*R*)-10-hydroxyoctadec-8-enoic acid ((*R*)-**2**)

Compound (*R*)-**2** was synthesized from (*R*,*Z*)-**S2** in a manner similar to the synthesis of *rac*-**3**. Approx. 2% of *E*-isomer of (*R*)-**2** was contaminated.

The ^{1}H and ^{13}C NMR spectra and HRMS data for the (*R*)-**2** were identical to those of the *rac*-**2**.

$[\alpha]^{24}_{D} = +20.83$ (c = 0.075, $CHCl_3$)

(8*Z*,10*R*)-10-acetoxyoctadec-8-enoic acid ((*R*)-**1**)

Compound (*R*)-**1** was synthesized from (*R*)-**2** in a manner similar to the synthesis of *rac*-**1**. The ^{1}H and ^{13}C NMR spectra and HRMS data for the (*R*)-**1** were identical to those of *rac*-**1**.

$[\alpha]^{24}_{D} = -1.67$ (c = 0.125, $CHCl_3$)

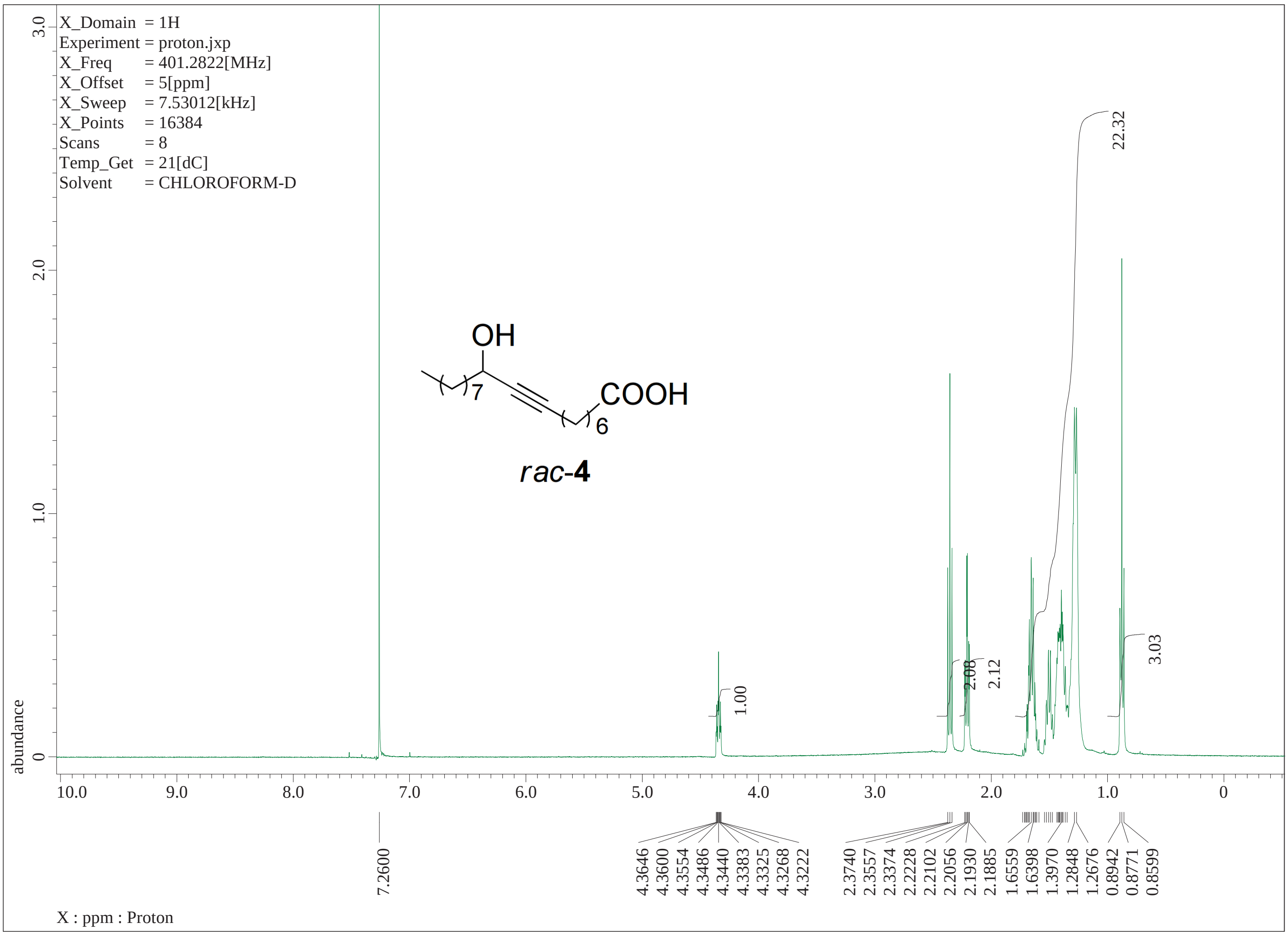
X_Domain = 1H
Experiment = proton.jxp
X_Freq = 401.2822[MHz]
X_Offset = 5[ppm]
X_Sweep = 7.53012[kHz]
X_Points = 16384
Scans = 8
Temp_Get = 21[dC]
Solvent = CHLOROFORM-D
OH
COOH
rac-4
22.32
1.00
2.08
2.12
3.03
7.2600
4.3646 4.3600 4.3554 4.3486 4.3440 4.3383 4.3325 4.3268 4.3222
2.3740 2.3557 2.3374 2.2228 2.2102 2.2056 2.1930 2.1885
1.6559 1.6398 1.3970 1.2848 1.2676
0.8942 0.8771 0.8599
abundance
X : ppm : Proton

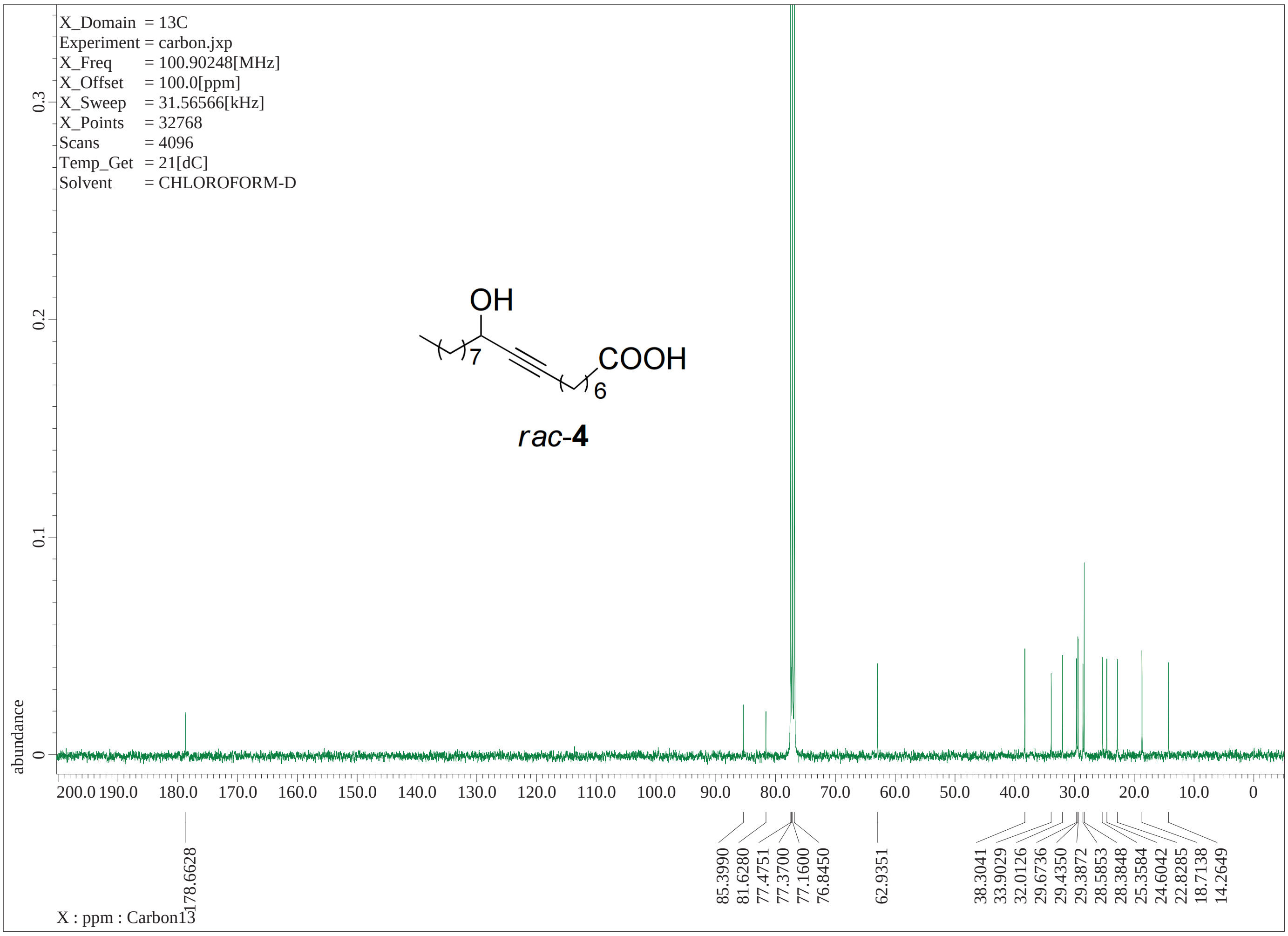
X_Domain = 13C
Experiment = carbon.jxp
X_Freq = 100.90248[MHz]
X_Offset = 100.0[ppm]
X_Sweep = 31.56566[kHz]
X_Points = 32768
Scans = 4096
Temp_Get = 21[dC]
Solvent = CHLOROFORM-D
OH
COOH
rac-4
178.6628
85.3990 81.6280 77.4751 77.3700 77.1600 76.8450
62.9351
38.3041 33.9029 32.0126 29.6736 29.4350 29.3872 28.5853 28.3848 25.3584 24.6042 22.8285 18.7138 14.2649
abundance
X : ppm : Carbon13

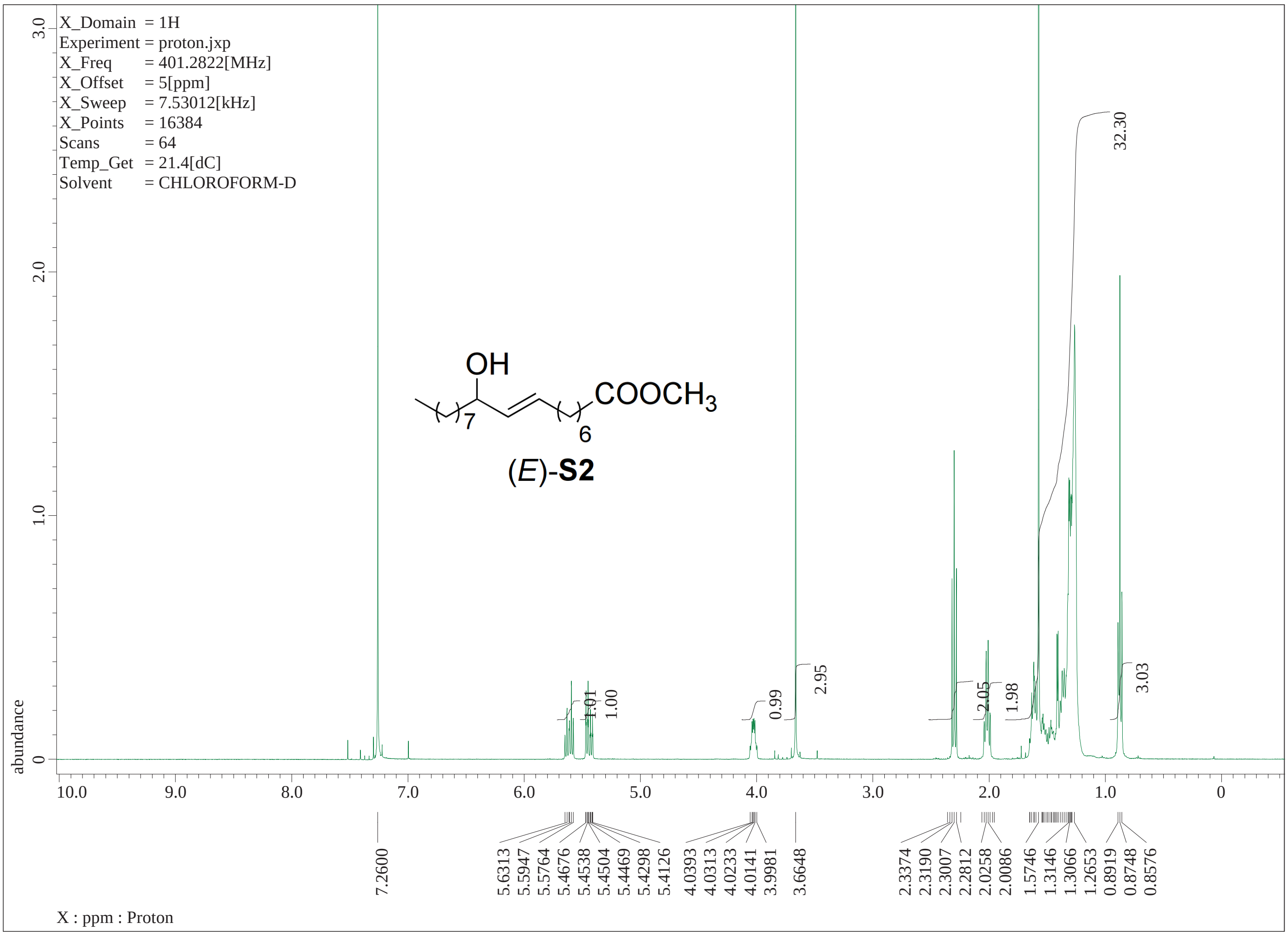
X_Domain = 1H
Experiment = proton.jxp
X_Freq = 401.2822[MHz]
X_Offset = 5[ppm]
X_Sweep = 7.53012[kHz]
X_Points = 16384
Scans = 64
Temp_Get = 21.4[dC]
Solvent = CHLOROFORM-D
OH
COOCH3
7
6
(E)-S2
abundance
X : ppm : Proton
32.30
2.95
0.99
1.01
1.00
2.05
1.98
3.03
7.2600
5.6313
5.5947
5.5764
5.4676
5.4538
5.4504
5.4469
5.4298
5.4126
4.0393
4.0313
4.0233
4.0141
3.9981
3.6648
2.3374
2.3190
2.3007
2.2812
2.0258
2.0086
1.5746
1.3146
1.3066
1.2653
0.8919
0.8748
0.8576

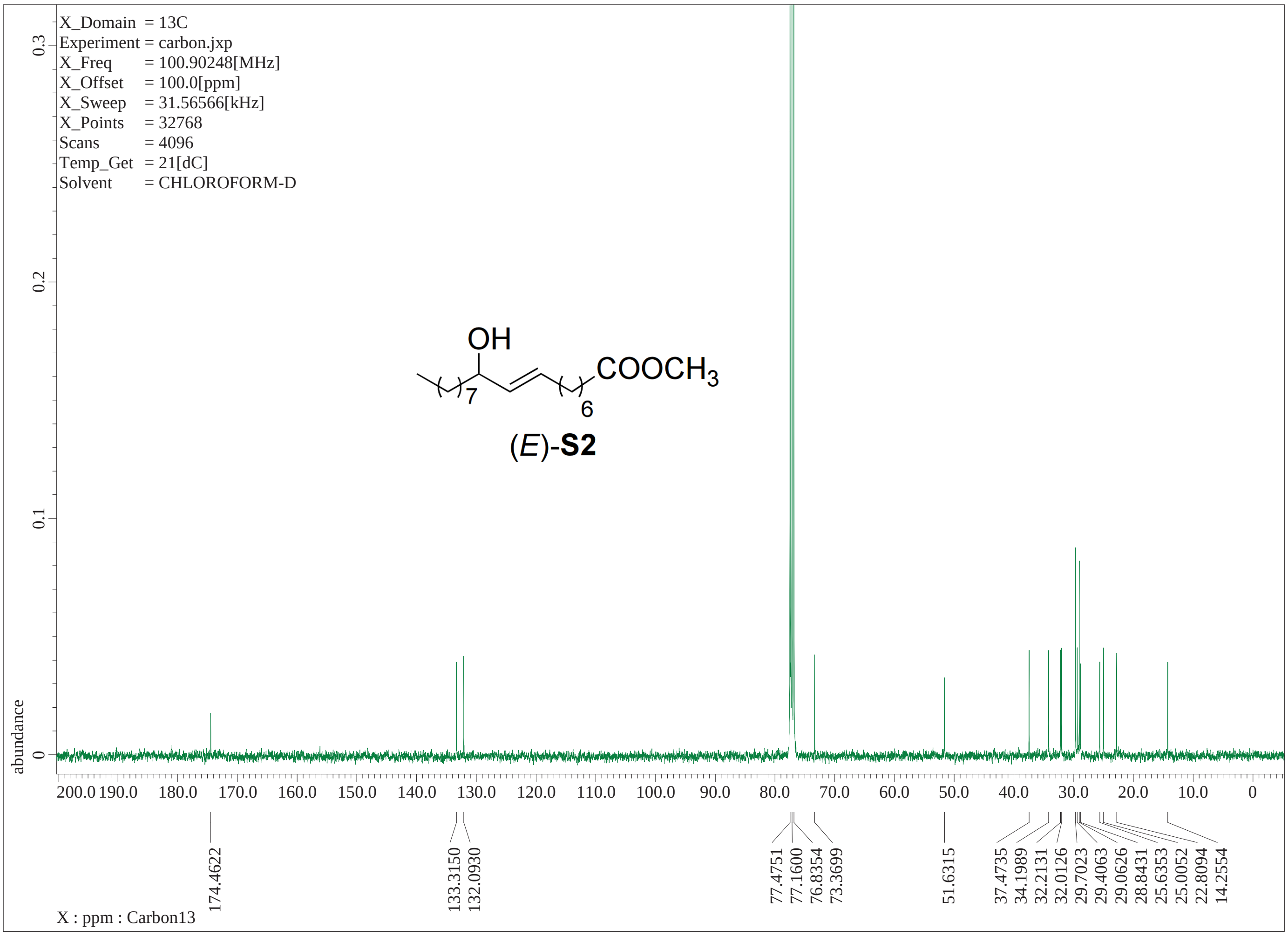
X_Domain = 13C
Experiment = carbon.jxp
X_Freq = 100.90248[MHz]
X_Offset = 100.0[ppm]
X_Sweep = 31.56566[kHz]
X_Points = 32768
Scans = 4096
Temp_Get = 21[dC]
Solvent = CHLOROFORM-D
OH
COOCH3
7
6
(E)-S2
abundance
X : ppm : Carbon13
174.4622
133.3150
132.0930
77.4751
77.1600
76.8354
73.3699
51.6315
37.4735
34.1989
32.2131
32.0126
29.7023
29.4063
29.0626
28.8431
25.6353
25.0052
22.8094
14.2554

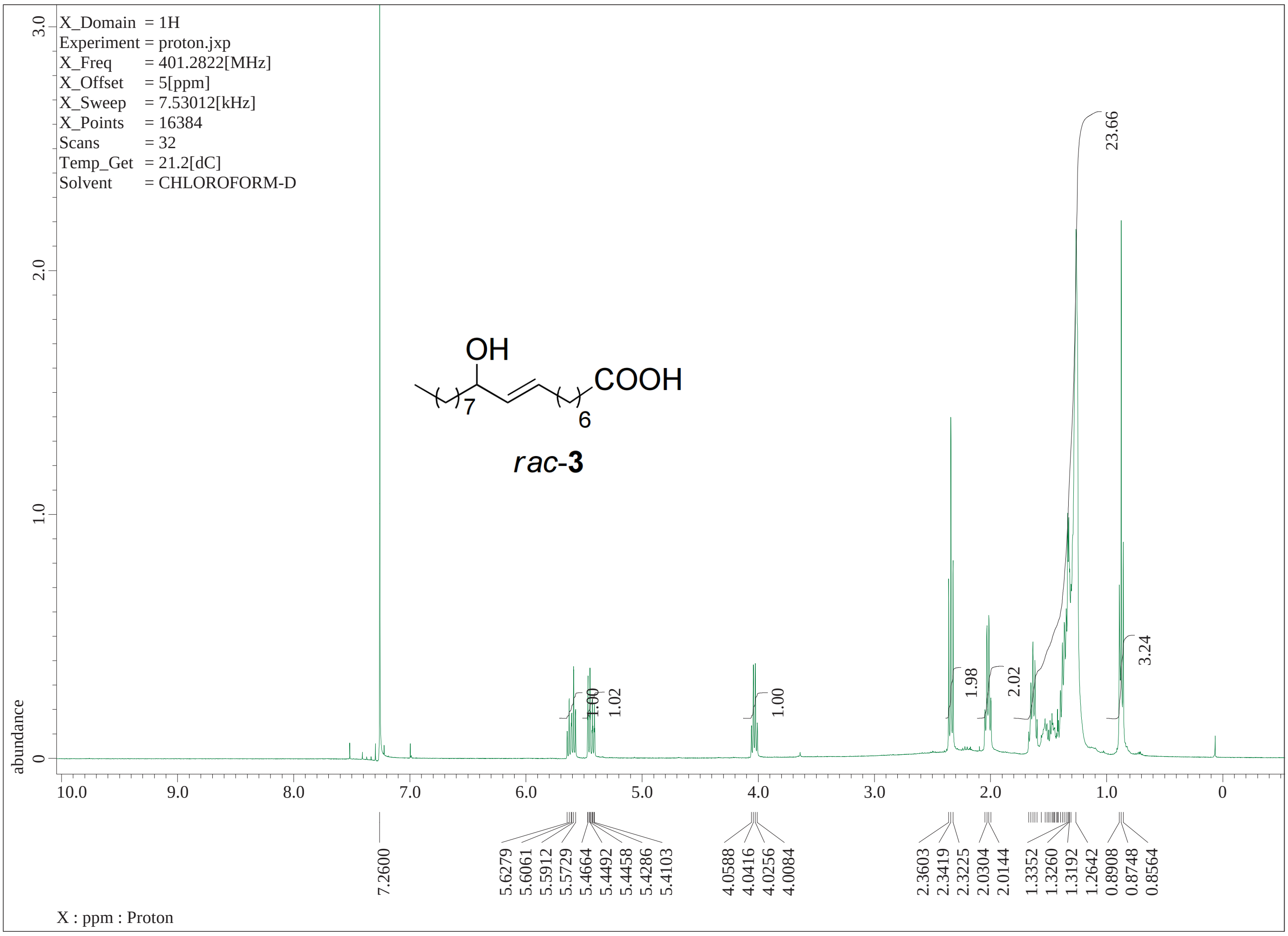
X_Domain = 1H
Experiment = proton.jxp
X_Freq = 401.2822[MHz]
X_Offset = 5[ppm]
X_Sweep = 7.53012[kHz]
X_Points = 16384
Scans = 32
Temp_Get = 21.2[dC]
Solvent = CHLOROFORM-D
OH
COOH
7
6
rac-3
abundance
23.66
3.24
1.98
2.02
1.00
1.02
1.00
7.2600
5.6279
5.6061
5.5912
5.5729
5.4664
5.4492
5.4458
5.4286
5.4103
4.0588
4.0416
4.0256
4.0084
2.3603
2.3419
2.3225
2.0304
2.0144
1.3352
1.3260
1.3192
1.2642
0.8908
0.8748
0.8564
X : ppm : Proton

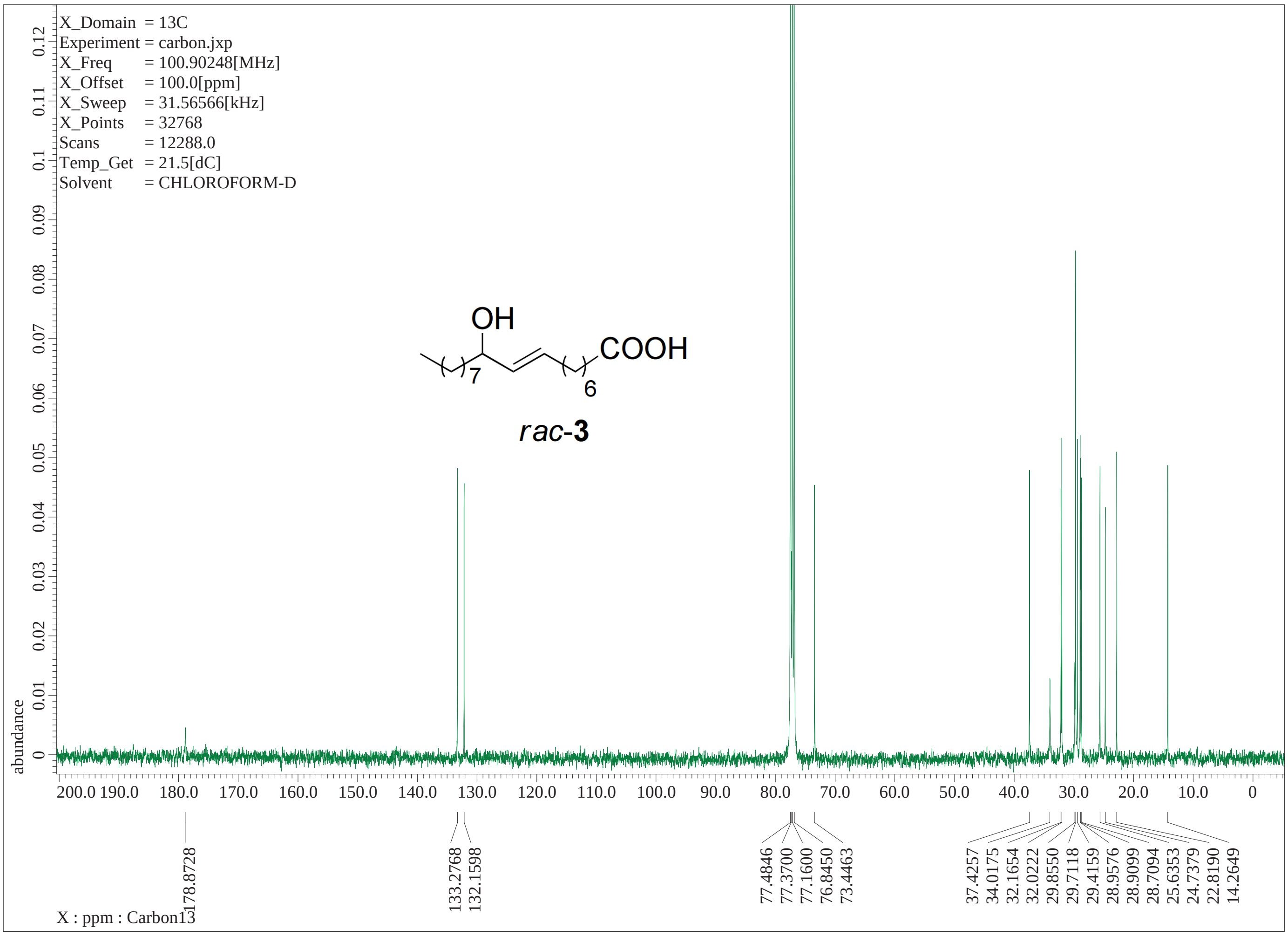
X_Domain = 13C
Experiment = carbon.jxp
X_Freq = 100.90248[MHz]
X_Offset = 100.0[ppm]
X_Sweep = 31.56566[kHz]
X_Points = 32768
Scans = 12288.0
Temp_Get = 21.5[dC]
Solvent = CHLOROFORM-D
OH
COOH
7
6
rac-3
abundance
178.8728
133.2768
132.1598
77.4846
77.3700
77.1600
76.8450
73.4463
37.4257
34.0175
32.1654
32.0222
29.8550
29.7118
29.4159
28.9576
28.9099
28.7094
25.6353
24.7379
22.8190
14.2649
X : ppm : Carbon13

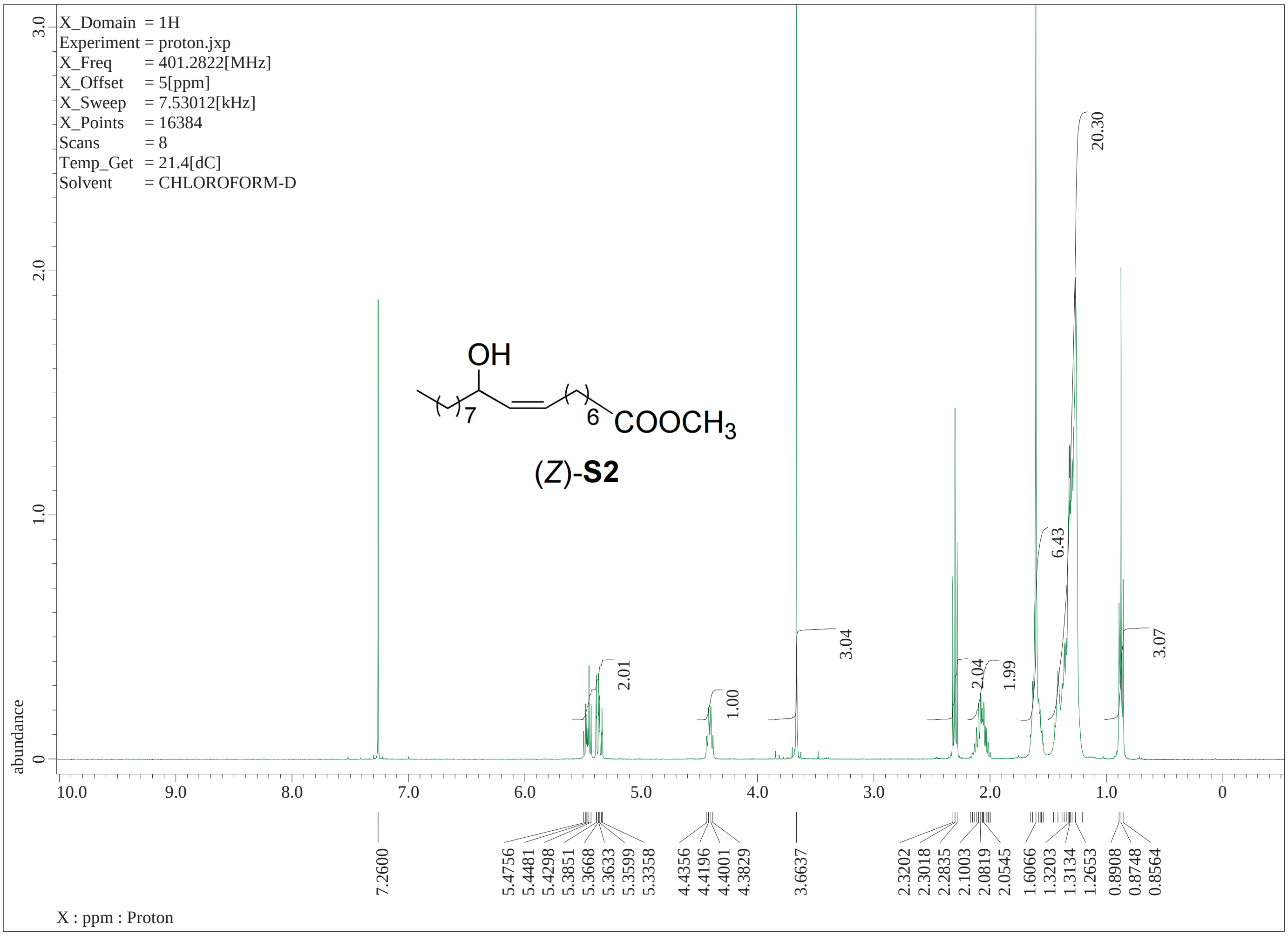
X_Domain = 1H
Experiment = proton.jxp
X_Freq = 401.2822[MHz]
X_Offset = 5[ppm]
X_Sweep = 7.53012[kHz]
X_Points = 16384
Scans = 8
Temp_Get = 21.4[dC]
Solvent = CHLOROFORM-D
OH
7
6
$COOCH_3$
(*Z*)-**S2**
2.01
1.00
3.04
2.04
1.99
6.43
20.30
3.07
abundance
7.2600
5.4756 5.4481 5.4298 5.3851 5.3668 5.3633 5.3599 5.3358
4.4356 4.4196 4.4001 4.3829
3.6637
2.3202 2.3018 2.2835 2.1003 2.0819 2.0545
1.6066 1.3203 1.3134 1.2653
0.8908 0.8748 0.8564
X : ppm : Proton

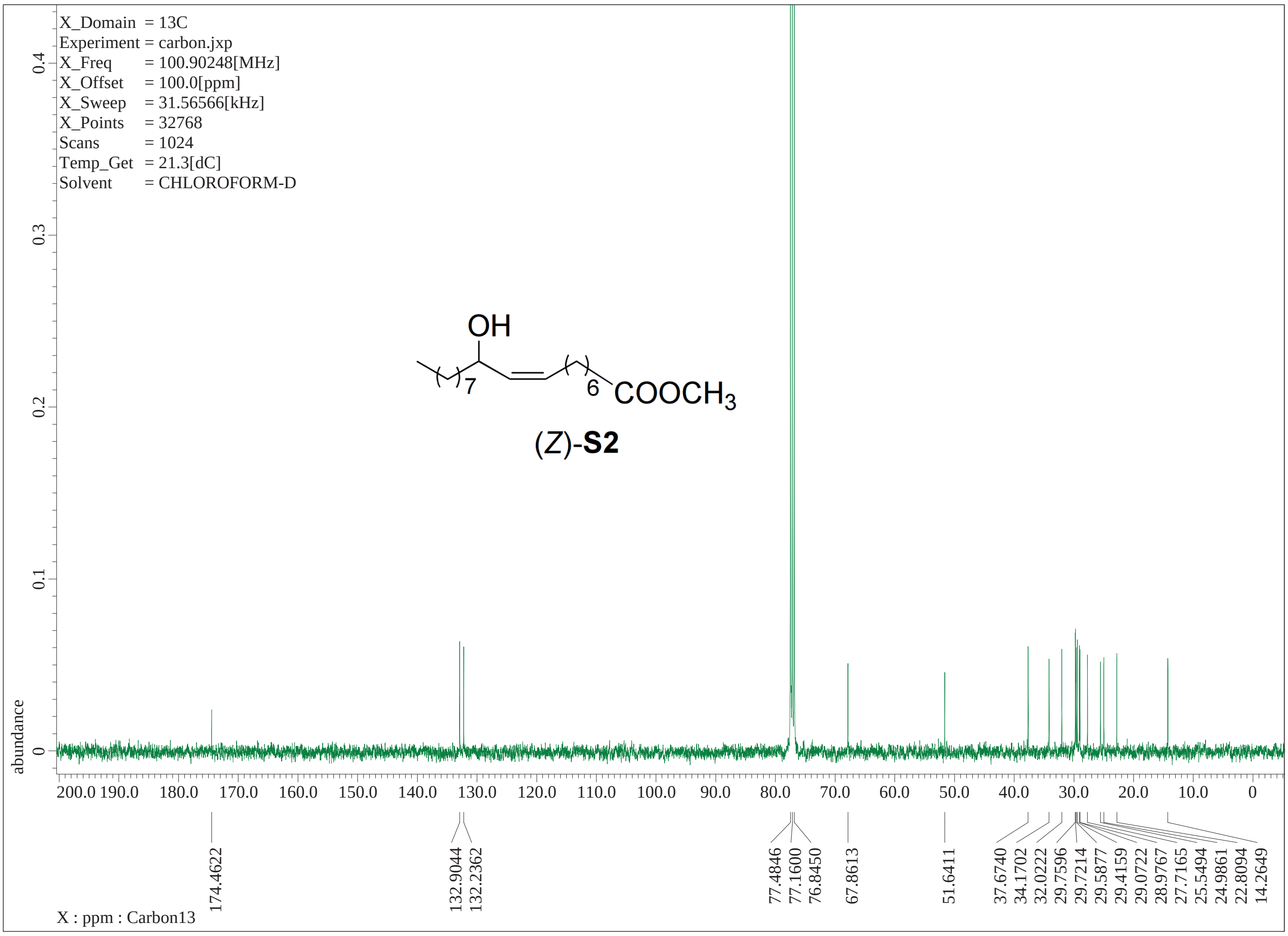
X_Domain = 13C
Experiment = carbon.jxp
X_Freq = 100.90248[MHz]
X_Offset = 100.0[ppm]
X_Sweep = 31.56566[kHz]
X_Points = 32768
Scans = 1024
Temp_Get = 21.3[dC]
Solvent = CHLOROFORM-D
OH
7
6
$COOCH_3$
(*Z*)-**S2**
abundance
174.4622
132.9044 132.2362
77.4846 77.1600 76.8450
67.8613
51.6411
37.6740 34.1702 32.0222 29.7596 29.7214 29.5877 29.4159 29.0722 28.9767 27.7165 25.5494 24.9861 22.8094 14.2649
X : ppm : Carbon13

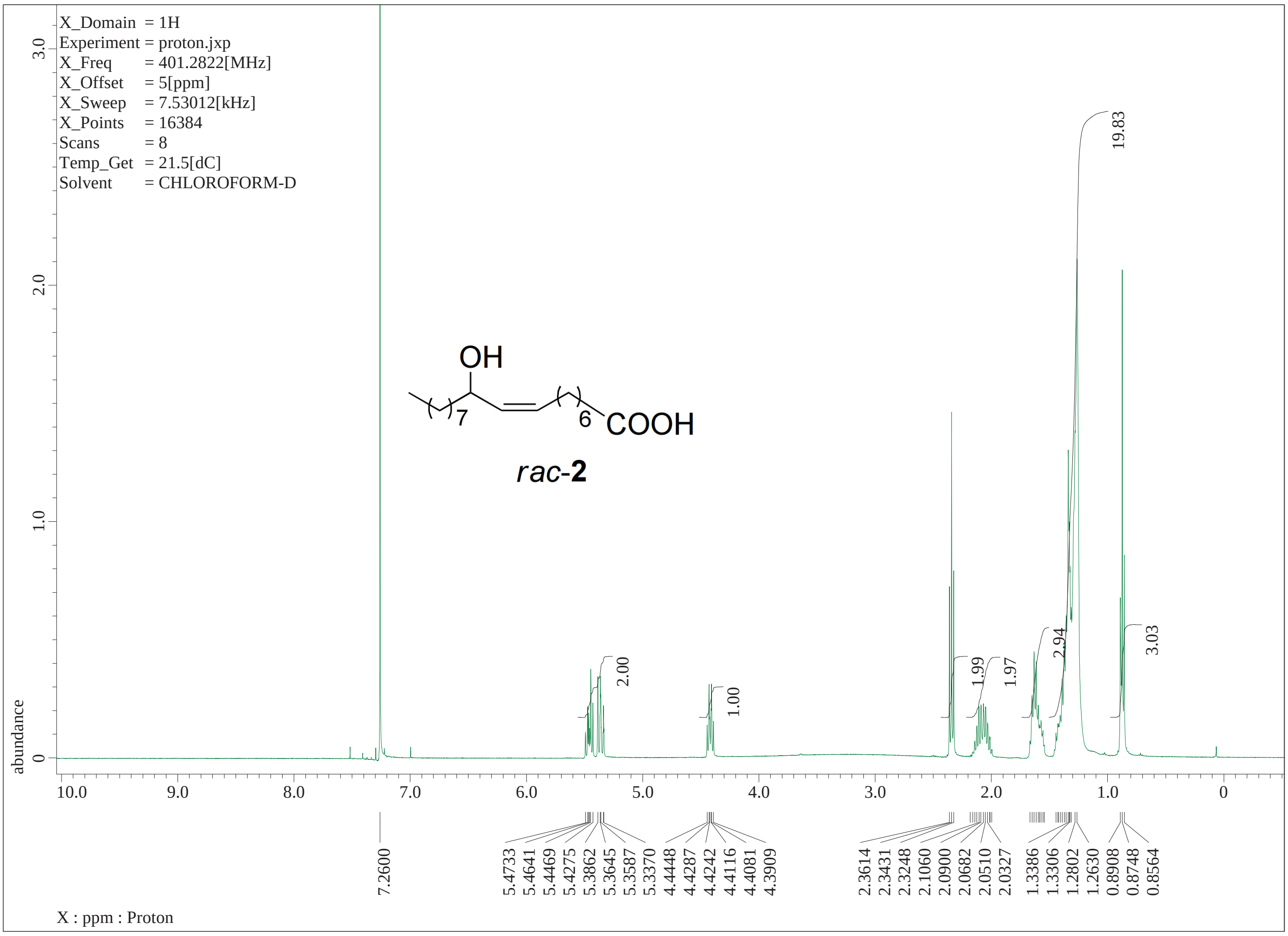
X_Domain = 1H
Experiment = proton.jxp
X_Freq = 401.2822[MHz]
X_Offset = 5[ppm]
X_Sweep = 7.53012[kHz]
X_Points = 16384
Scans = 8
Temp_Get = 21.5[dC]
Solvent = CHLOROFORM-D
OH
7
6
COOH
rac-2
abundance
X : ppm : Proton
2.00
1.00
1.99
1.97
2.94
19.83
3.03
7.2600
5.4733
5.4641
5.4469
5.4275
5.3862
5.3645
5.3587
5.3370
4.4448
4.4287
4.4242
4.4116
4.4081
4.3909
2.3614
2.3431
2.3248
2.1060
2.0900
2.0682
2.0510
2.0327
1.3386
1.3306
1.2802
1.2630
0.8908
0.8748
0.8564

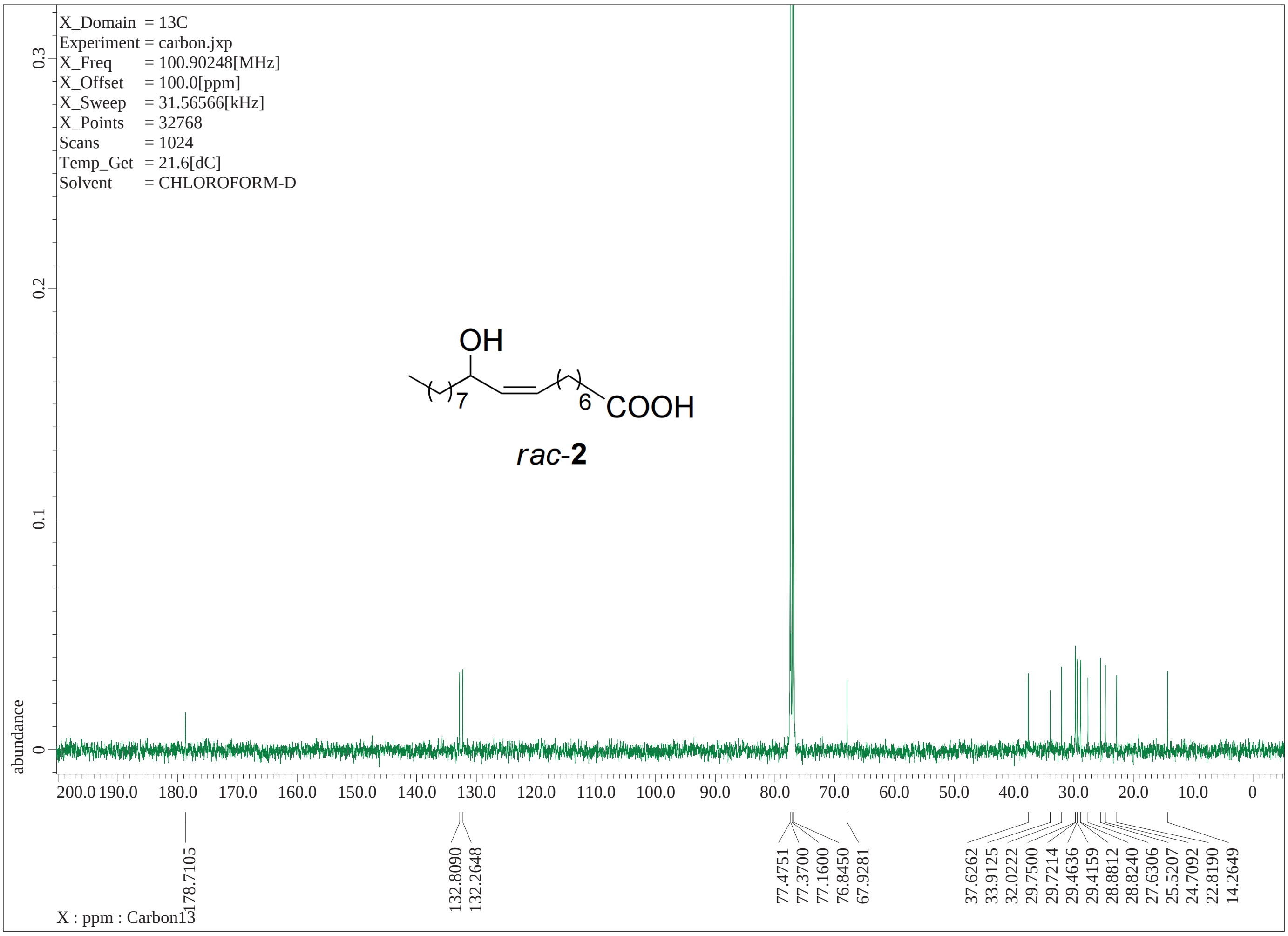
X_Domain = 13C
Experiment = carbon.jxp
X_Freq = 100.90248[MHz]
X_Offset = 100.0[ppm]
X_Sweep = 31.56566[kHz]
X_Points = 32768
Scans = 1024
Temp_Get = 21.6[dC]
Solvent = CHLOROFORM-D
OH
7
6
COOH
rac-2
abundance
X : ppm : Carbon13
178.7105
132.8090
132.2648
77.4751
77.3700
77.1600
76.8450
67.9281
37.6262
33.9125
32.0222
29.7500
29.7214
29.4636
29.4159
28.8812
28.8240
27.6306
25.5207
24.7092
22.8190
14.2649

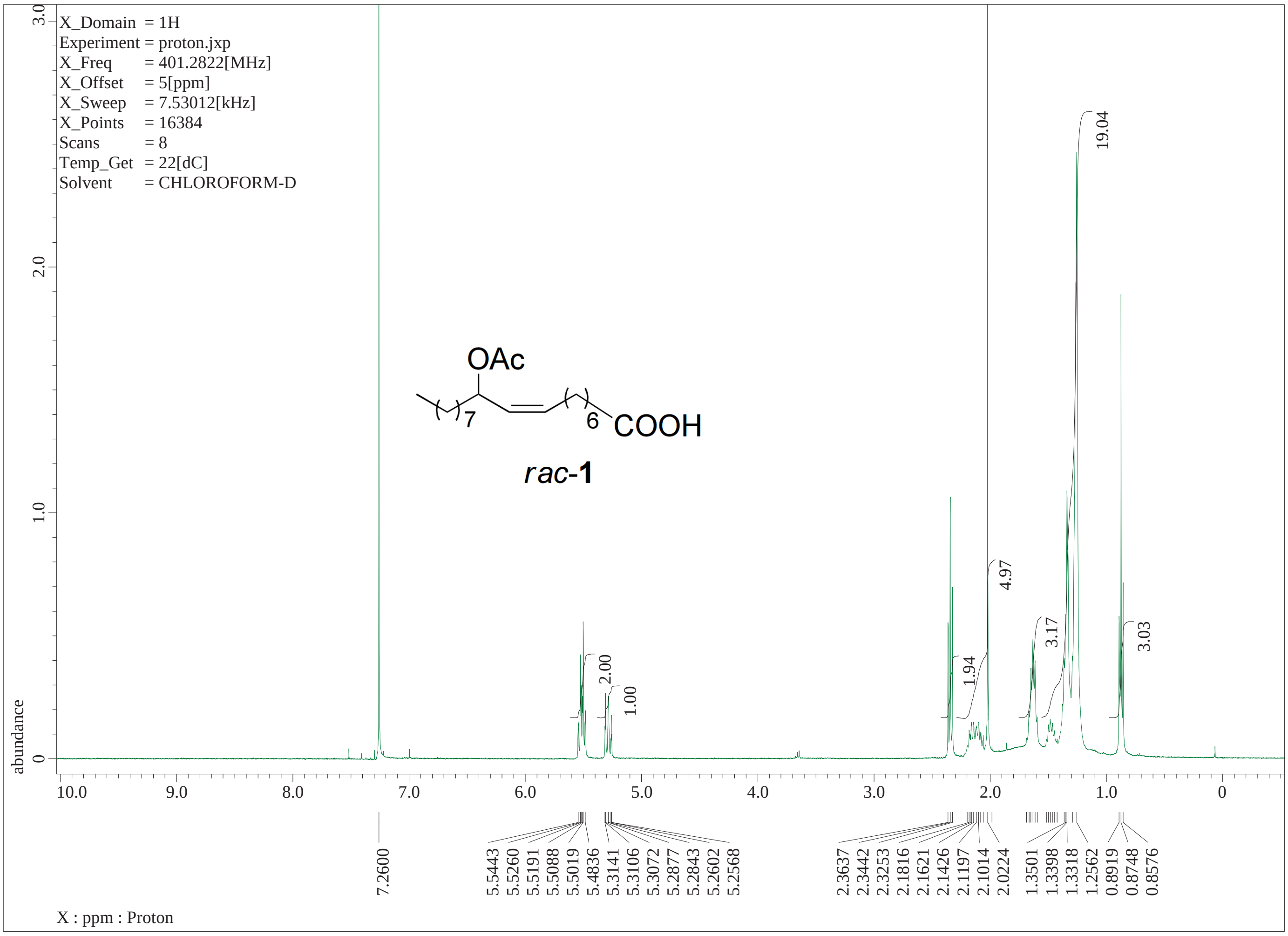
X_Domain = 1H
Experiment = proton.jxp
X_Freq = 401.2822[MHz]
X_Offset = 5[ppm]
X_Sweep = 7.53012[kHz]
X_Points = 16384
Scans = 8
Temp_Get = 22[dC]
Solvent = CHLOROFORM-D
OAc
7
6
COOH
rac-**1**
19.04
4.97
3.17
3.03
2.00
1.00
1.94
abundance
X : ppm : Proton
7.2600
5.5443
5.5260
5.5191
5.5088
5.5019
5.4836
5.3141
5.3106
5.3072
5.2877
5.2843
5.2602
5.2568
2.3637
2.3442
2.3253
2.1816
2.1621
2.1426
2.1197
2.1014
2.0224
1.3501
1.3398
1.3318
1.2562
0.8919
0.8748
0.8576

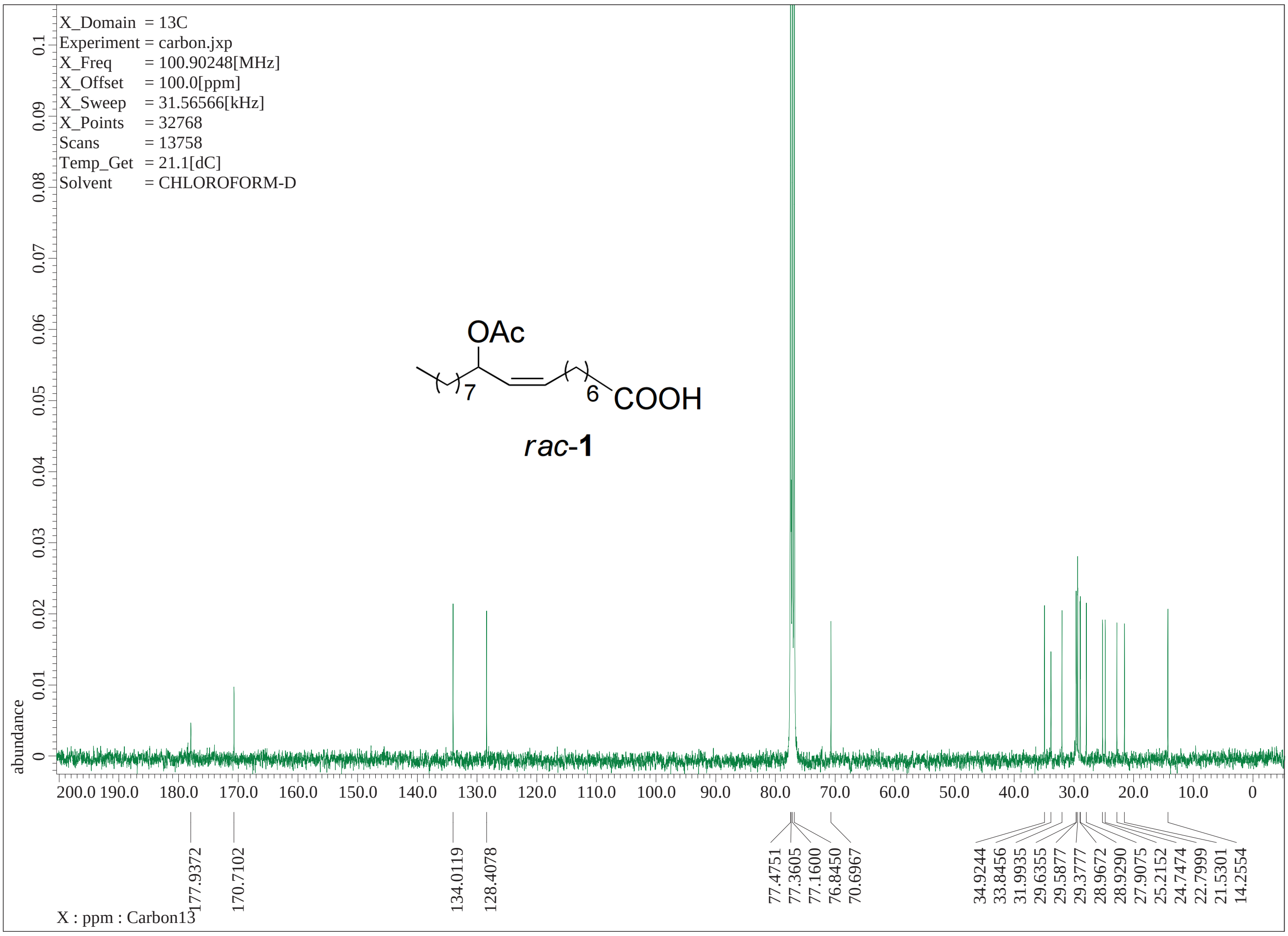
X_Domain = 13C
Experiment = carbon.jxp
X_Freq = 100.90248[MHz]
X_Offset = 100.0[ppm]
X_Sweep = 31.56566[kHz]
X_Points = 32768
Scans = 13758
Temp_Get = 21.1[dC]
Solvent = CHLOROFORM-D
OAc
7
6
COOH
rac-**1**
abundance
X : ppm : Carbon13
177.9372
170.7102
134.0119
128.4078
77.4751
77.3605
77.1600
76.8450
70.6967
34.9244
33.8456
31.9935
29.6355
29.5877
29.3777
28.9672
28.9290
27.9075
25.2152
24.7474
22.7999
21.5301
14.2554

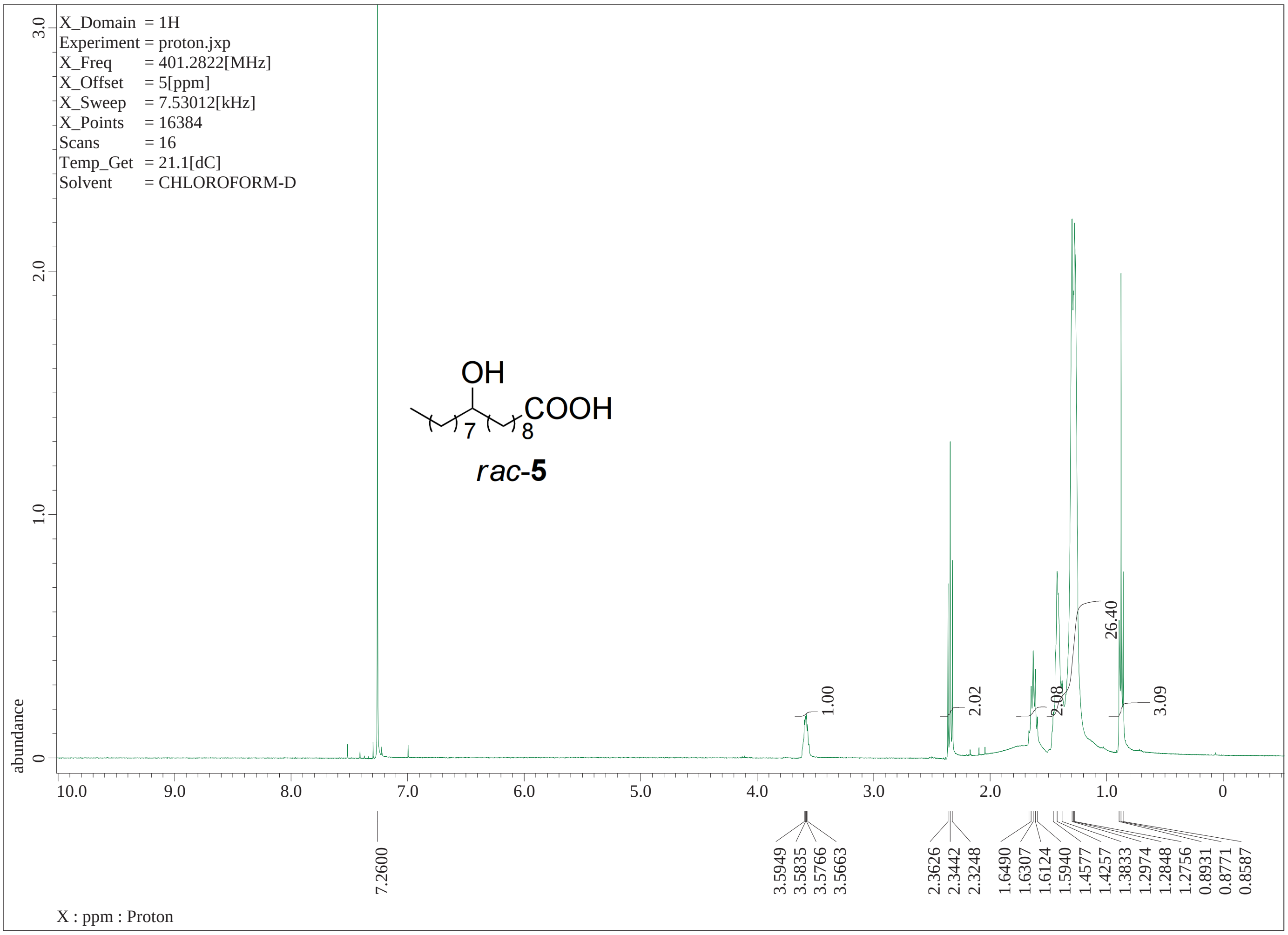
X_Domain = 1H
Experiment = proton.jxp
X_Freq = 401.2822[MHz]
X_Offset = 5[ppm]
X_Sweep = 7.53012[kHz]
X_Points = 16384
Scans = 16
Temp_Get = 21.1[dC]
Solvent = CHLOROFORM-D
OH
COOH
7
8
rac-5
abundance
1.00
2.02
2.08
26.40
3.09
7.2600
3.5949
3.5835
3.5766
3.5663
2.3626
2.3442
2.3248
1.6490
1.6307
1.6124
1.5940
1.4577
1.4257
1.3833
1.2974
1.2848
1.2756
0.8931
0.8771
0.8587
X : ppm : Proton

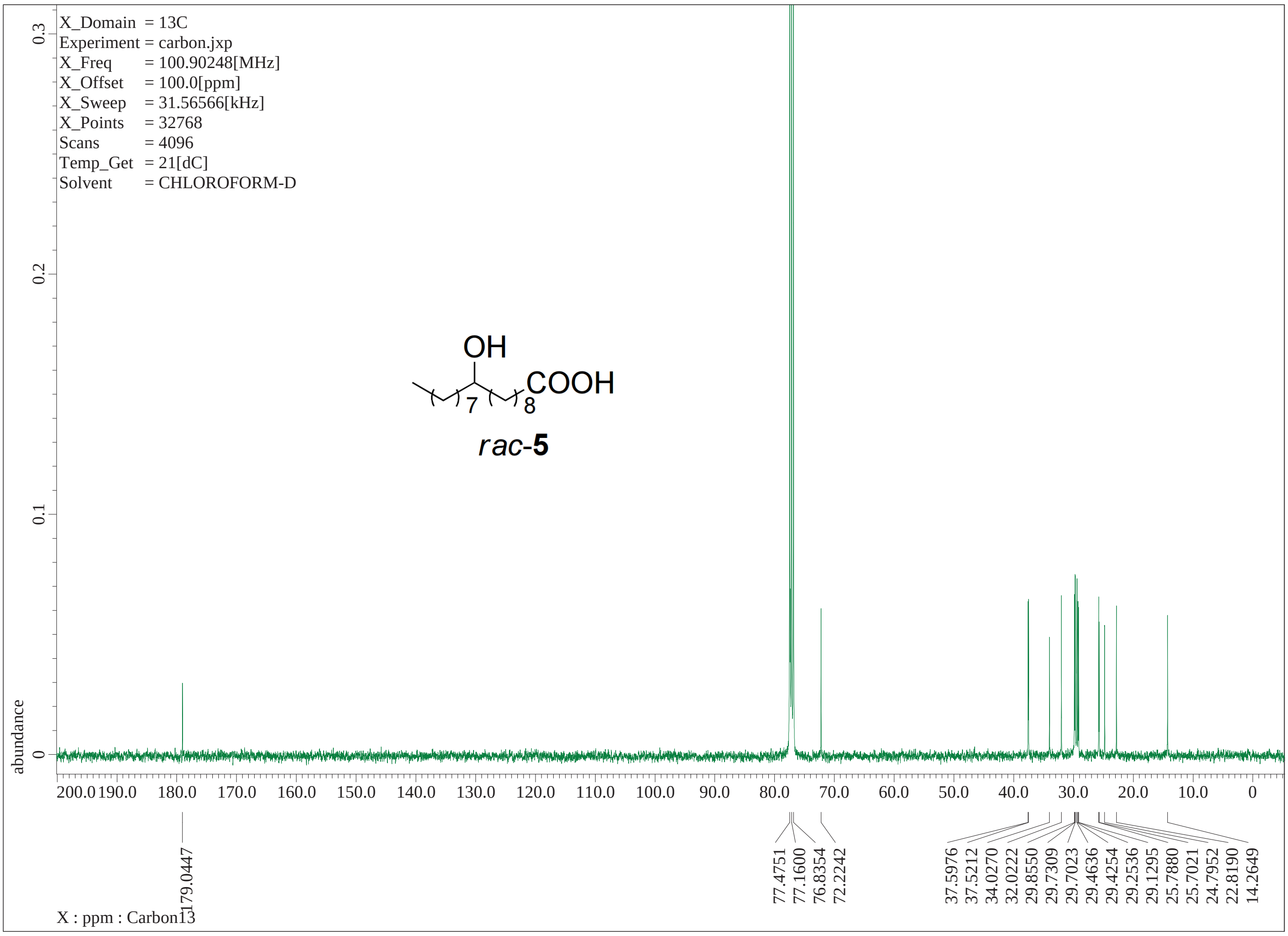
X_Domain = 13C
Experiment = carbon.jxp
X_Freq = 100.90248[MHz]
X_Offset = 100.0[ppm]
X_Sweep = 31.56566[kHz]
X_Points = 32768
Scans = 4096
Temp_Get = 21[dC]
Solvent = CHLOROFORM-D
OH
COOH
7
8
rac-5
abundance
179.0447
77.4751
77.1600
76.8354
72.2242
37.5976
37.5212
34.0270
32.0222
29.8550
29.7309
29.7023
29.4636
29.4254
29.2536
29.1295
25.7880
25.7021
24.7952
22.8190
14.2649
X : ppm : Carbon13

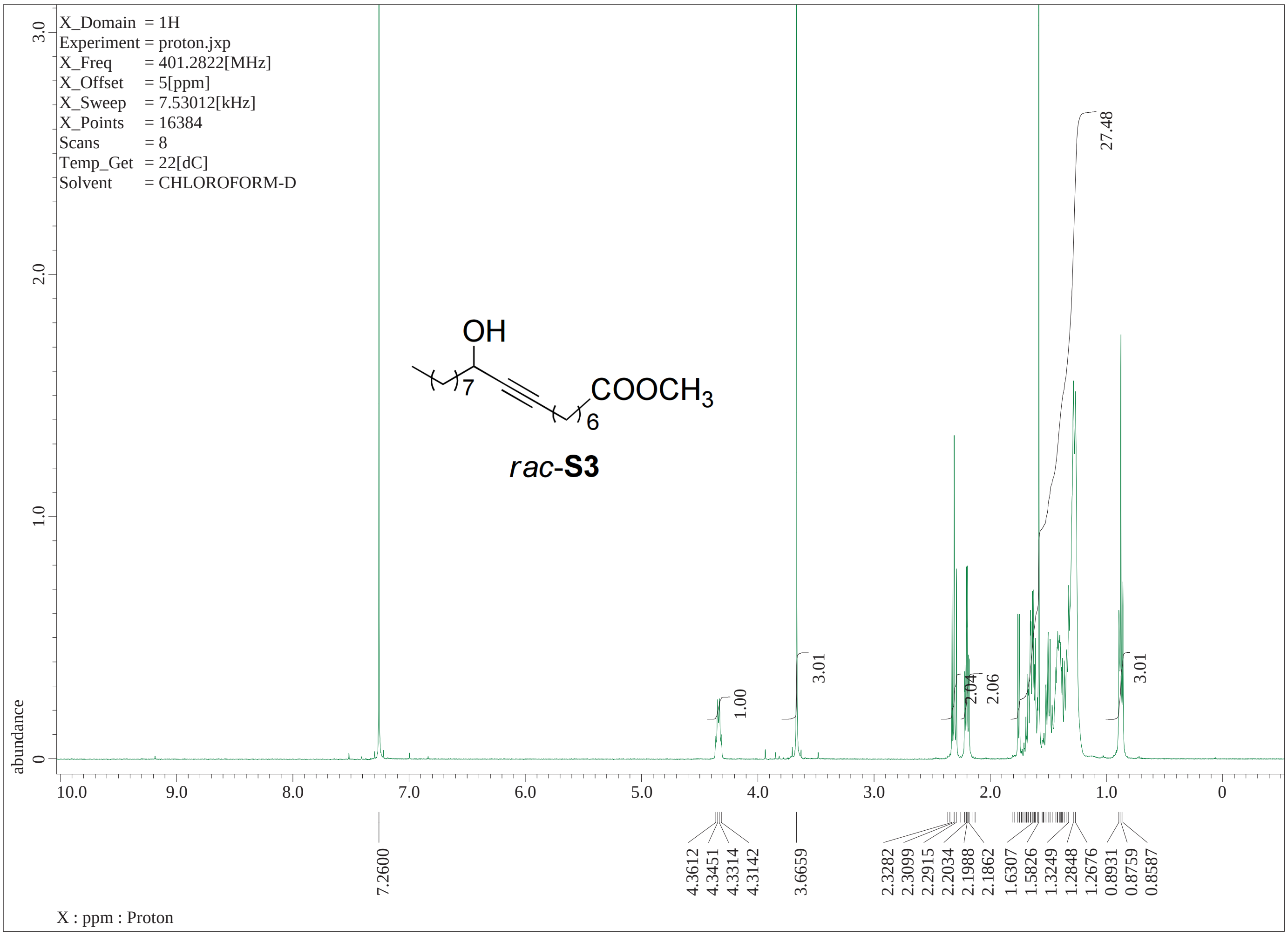
X_Domain = 1H
Experiment = proton.jxp
X_Freq = 401.2822[MHz]
X_Offset = 5[ppm]
X_Sweep = 7.53012[kHz]
X_Points = 16384
Scans = 8
Temp_Get = 22[dC]
Solvent = CHLOROFORM-D
OH
7
COOCH3
6
rac-S3
27.48
1.00
3.01
2.04
2.06
3.01
abundance
7.2600
4.3612
4.3451
4.3314
4.3142
3.6659
2.3282
2.3099
2.2915
2.2034
2.1988
2.1862
1.6307
1.5826
1.3249
1.2848
1.2676
0.8931
0.8759
0.8587
X : ppm : Proton

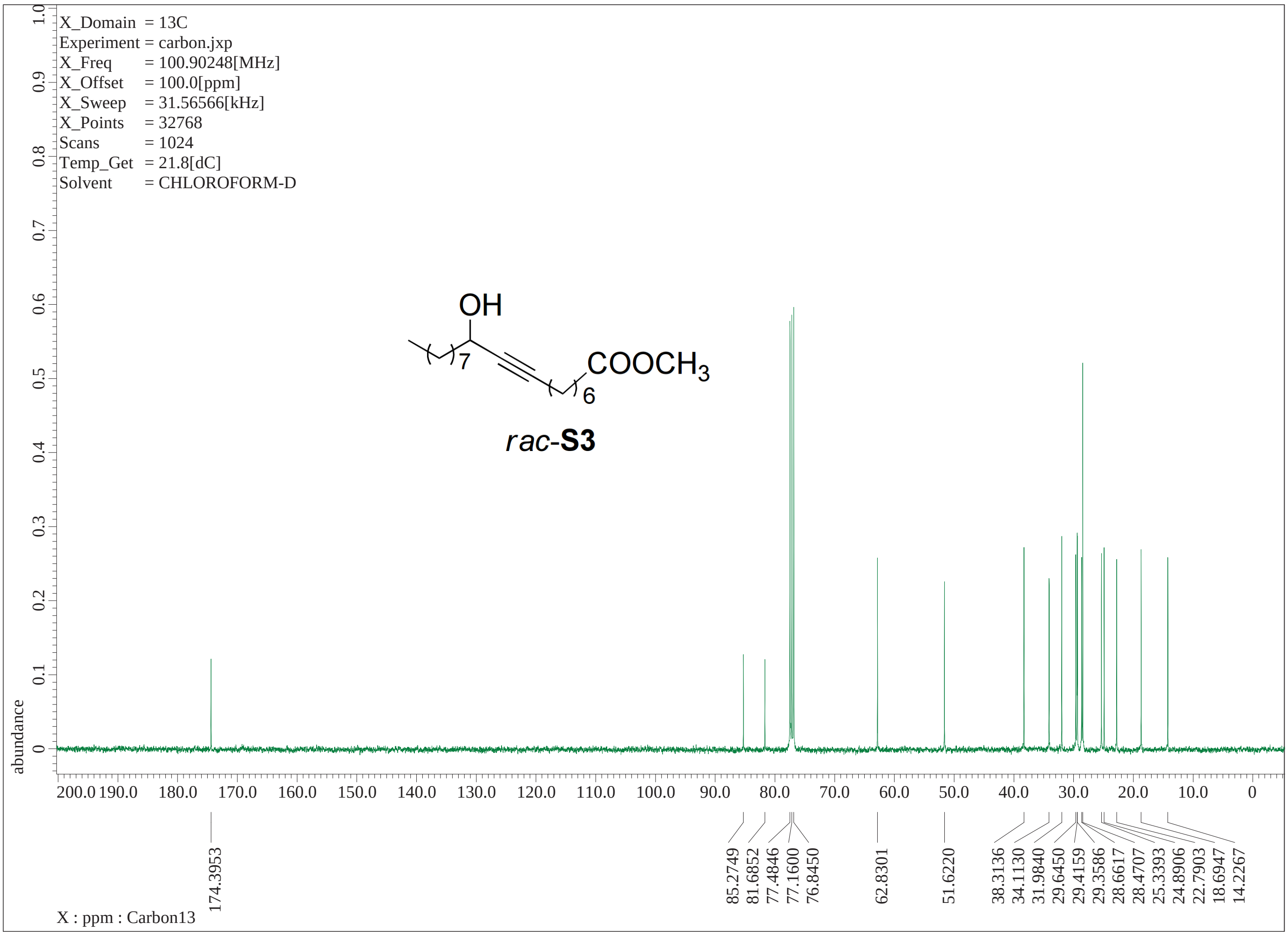
X_Domain = 13C
Experiment = carbon.jxp
X_Freq = 100.90248[MHz]
X_Offset = 100.0[ppm]
X_Sweep = 31.56566[kHz]
X_Points = 32768
Scans = 1024
Temp_Get = 21.8[dC]
Solvent = CHLOROFORM-D
OH
7
COOCH3
6
rac-S3
abundance
174.3953
85.2749
81.6852
77.4846
77.1600
76.8450
62.8301
51.6220
38.3136
34.1130
31.9840
29.6450
29.4159
29.3586
28.6617
28.4707
25.3393
24.8906
22.7903
18.6947
14.2267
X : ppm : Carbon13

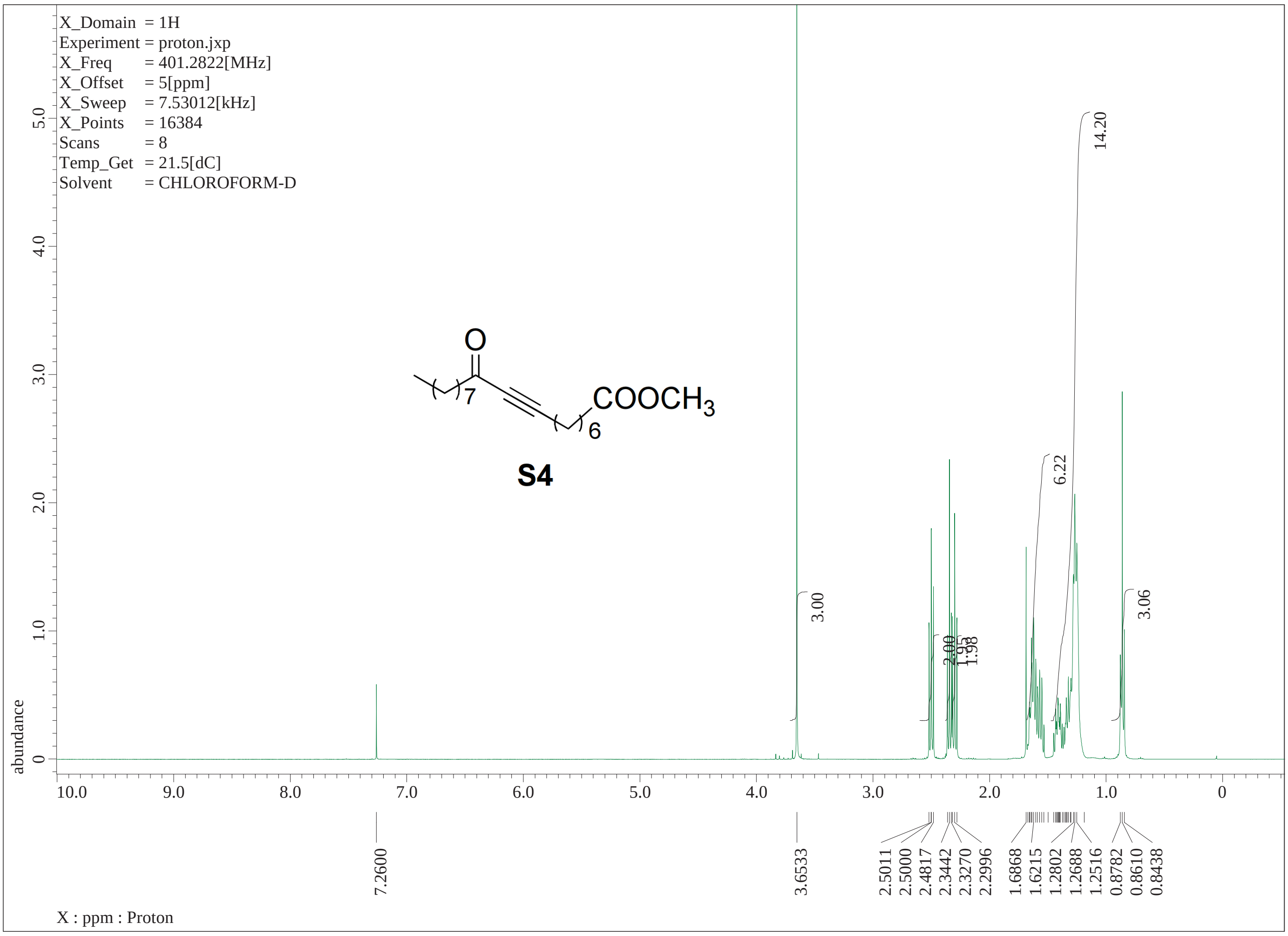
X_Domain = 1H
Experiment = proton.jxp
X_Freq = 401.2822[MHz]
X_Offset = 5[ppm]
X_Sweep = 7.53012[kHz]
X_Points = 16384
Scans = 8
Temp_Get = 21.5[dC]
Solvent = CHLOROFORM-D
O
7
COOCH3
6
S4
3.00
2.00
1.95
1.98
6.22
14.20
3.06
abundance
7.2600
3.6533
2.5011
2.5000
2.4817
2.3442
2.3270
2.2996
1.6868
1.6215
1.2802
1.2688
1.2516
0.8782
0.8610
0.8438
X : ppm : Proton

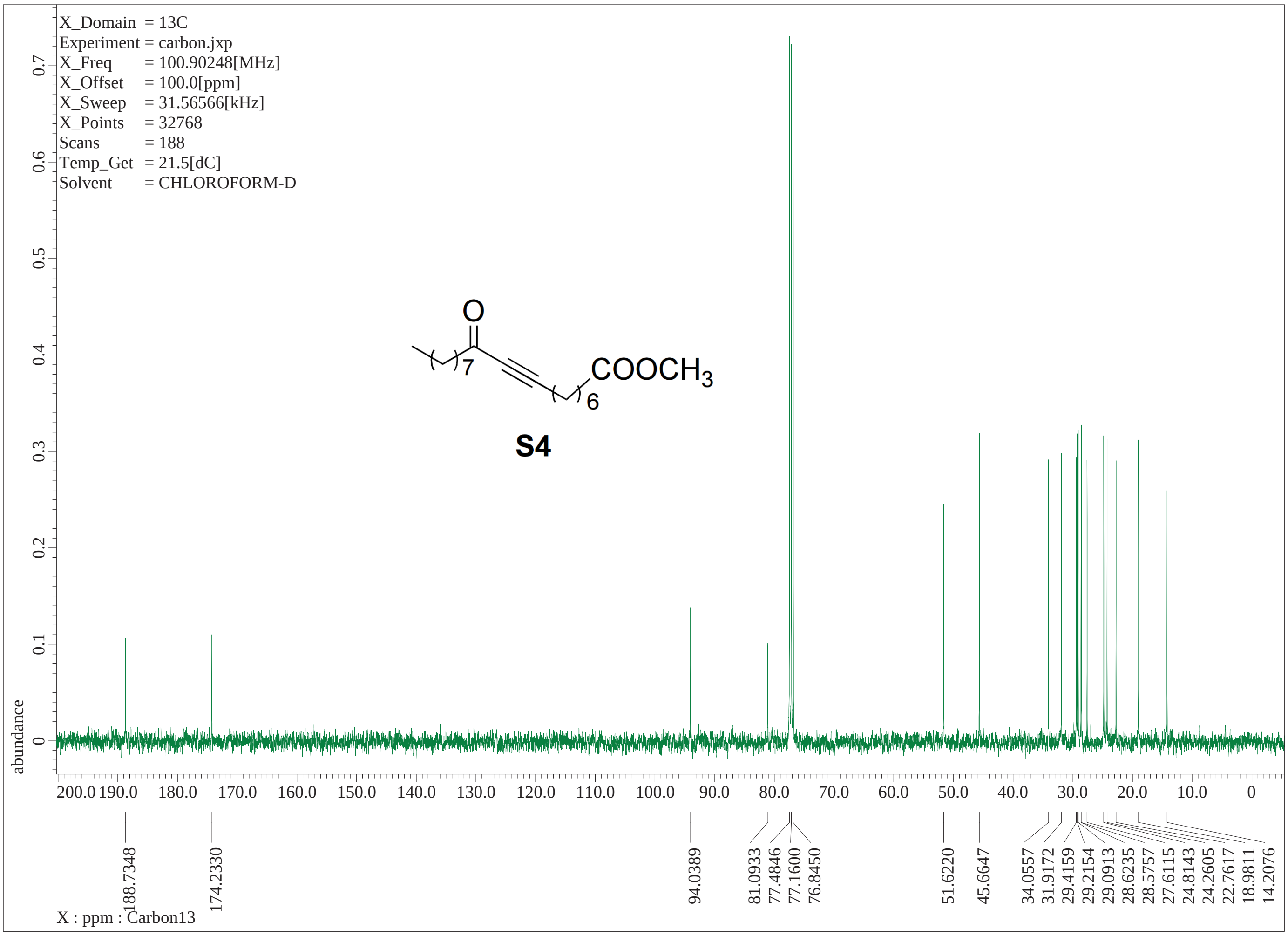
X_Domain = 13C
Experiment = carbon.jxp
X_Freq = 100.90248[MHz]
X_Offset = 100.0[ppm]
X_Sweep = 31.56566[kHz]
X_Points = 32768
Scans = 188
Temp_Get = 21.5[dC]
Solvent = CHLOROFORM-D
O
7
COOCH3
6
S4
abundance
188.7348
174.2330
94.0389
81.0933
77.4846
77.1600
76.8450
51.6220
45.6647
34.0557
31.9172
29.4159
29.2154
29.0913
28.6235
28.5757
27.6115
24.8143
24.2605
22.7617
18.9811
14.2076
X : ppm : Carbon13

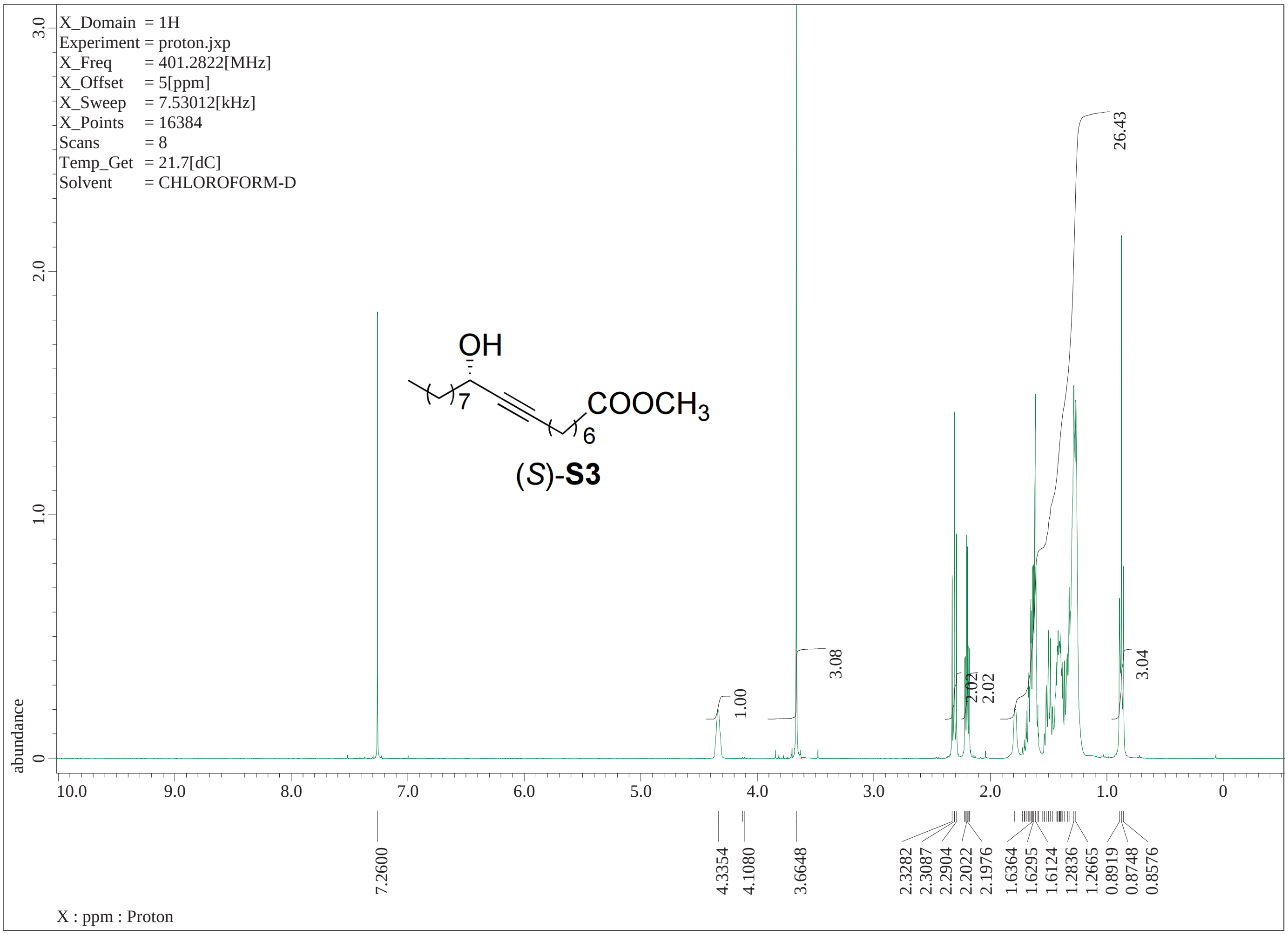
X_Domain = 1H
Experiment = proton.jxp
X_Freq = 401.2822[MHz]
X_Offset = 5[ppm]
X_Sweep = 7.53012[kHz]
X_Points = 16384
Scans = 8
Temp_Get = 21.7[dC]
Solvent = CHLOROFORM-D
OH
7
COOCH3
6
(S)-S3
26.43
1.00
3.08
2.02
2.02
3.04
abundance
7.2600
4.3354
4.1080
3.6648
2.3282
2.3087
2.2904
2.2022
2.1976
1.6364
1.6295
1.6124
1.2836
1.2665
0.8919
0.8748
0.8576
X : ppm : Proton

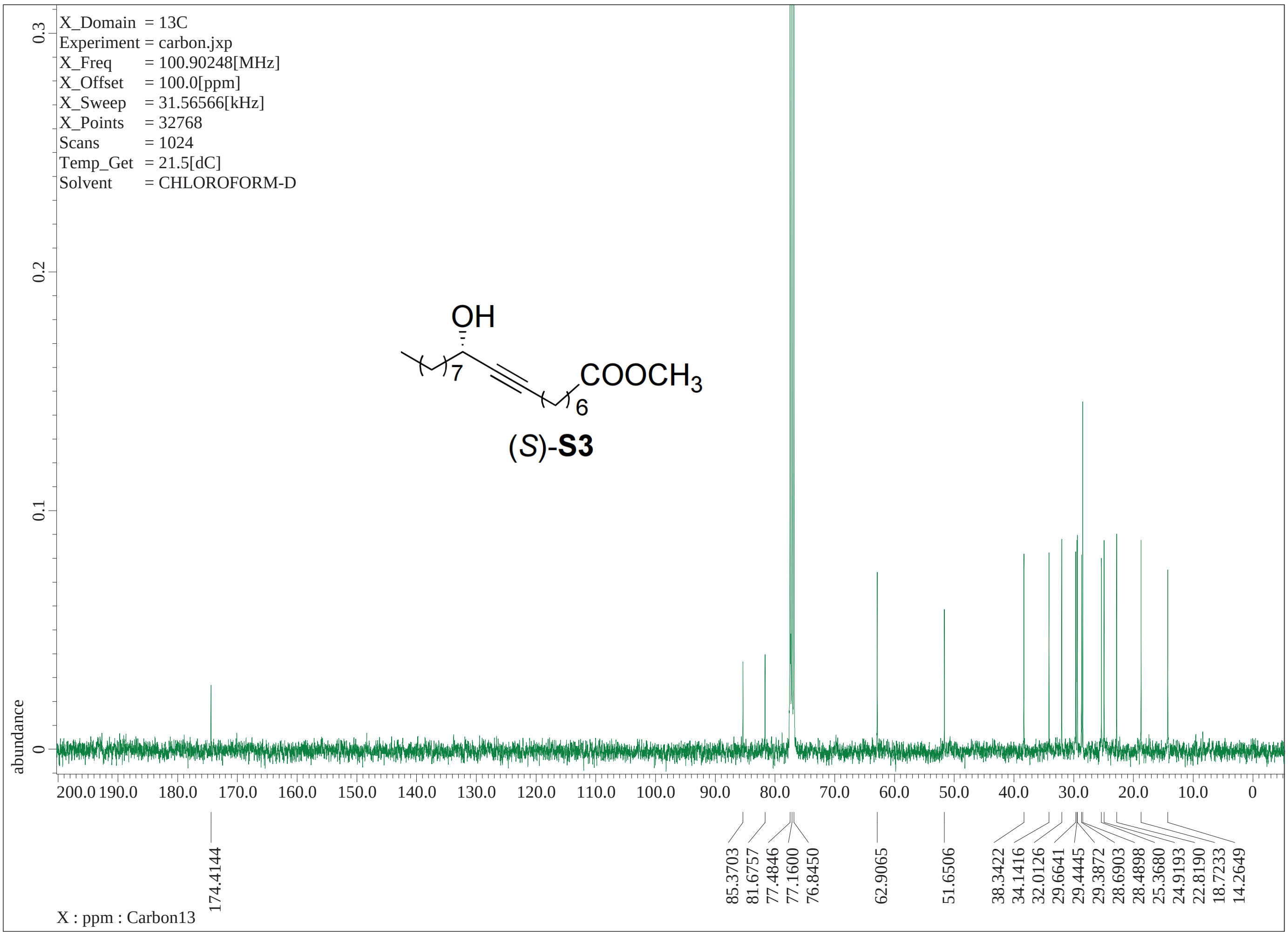
X_Domain = 13C
Experiment = carbon.jxp
X_Freq = 100.90248[MHz]
X_Offset = 100.0[ppm]
X_Sweep = 31.56566[kHz]
X_Points = 32768
Scans = 1024
Temp_Get = 21.5[dC]
Solvent = CHLOROFORM-D
OH
7
COOCH3
6
(S)-S3
abundance
174.4144
85.3703
81.6757
77.4846
77.1600
76.8450
62.9065
51.6506
38.3422
34.1416
32.0126
29.6641
29.4445
29.3872
28.6903
28.4898
25.3680
24.9193
22.8190
18.7233
14.2649
X : ppm : Carbon13

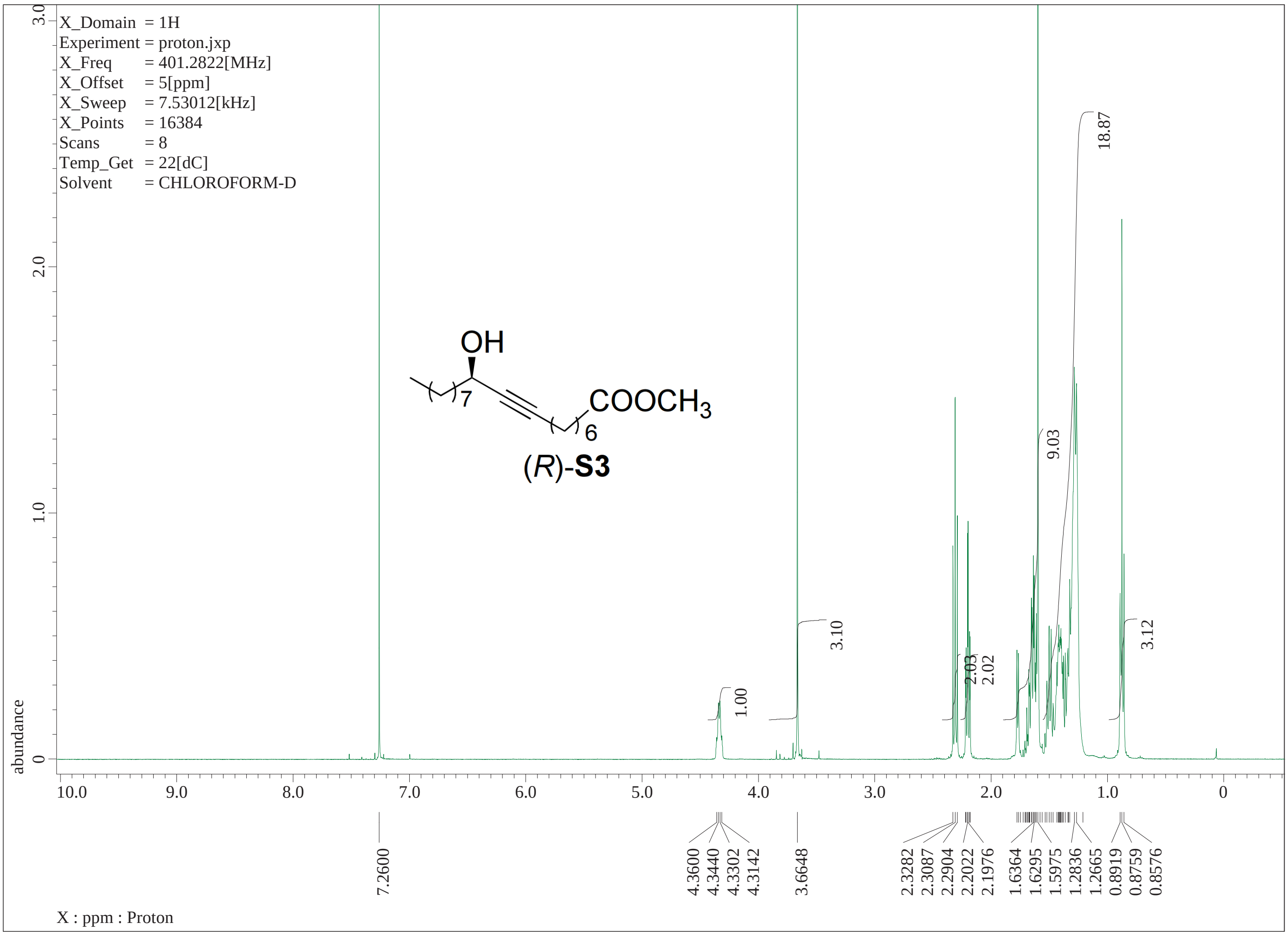
X_Domain = 1H
Experiment = proton.jxp
X_Freq = 401.2822[MHz]
X_Offset = 5[ppm]
X_Sweep = 7.53012[kHz]
X_Points = 16384
Scans = 8
Temp_Get = 22[dC]
Solvent = CHLOROFORM-D
OH
COOCH3
7
6
(R)-S3
abundance
X : ppm : Proton
7.2600
4.3600 4.3440 4.3302 4.3142
3.6648
2.3282 2.3087 2.2904 2.2022 2.1976
1.6364 1.6295 1.5975 1.2836 1.2665
0.8919 0.8759 0.8576
1.00
3.10
2.03
2.02
9.03
18.87
3.12

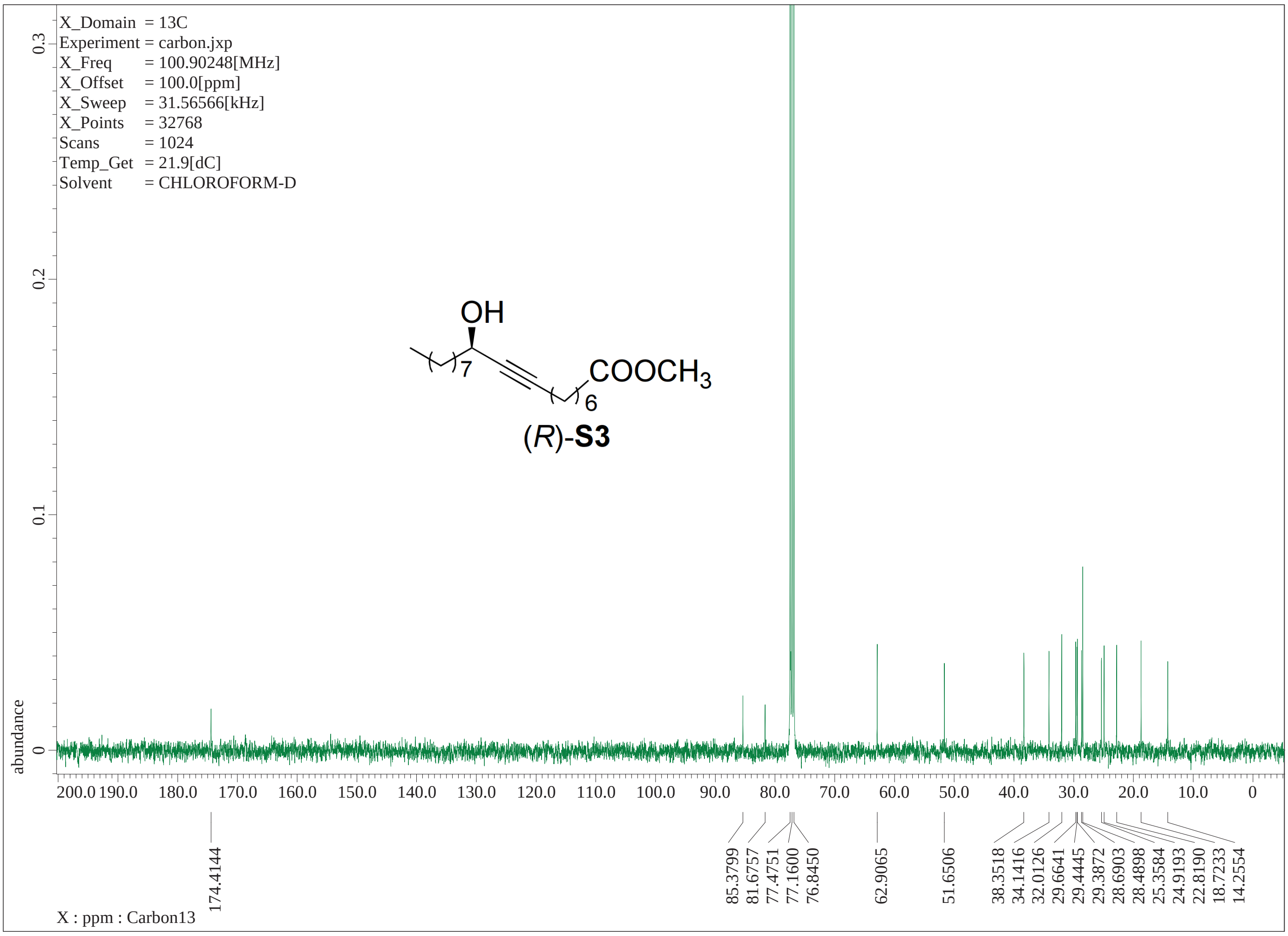
X_Domain = 13C
Experiment = carbon.jxp
X_Freq = 100.90248[MHz]
X_Offset = 100.0[ppm]
X_Sweep = 31.56566[kHz]
X_Points = 32768
Scans = 1024
Temp_Get = 21.9[dC]
Solvent = CHLOROFORM-D
OH
COOCH3
7
6
(R)-S3
abundance
X : ppm : Carbon13
174.4144
85.3799 81.6757 77.4751 77.1600 76.8450
62.9065
51.6506
38.3518 34.1416 32.0126 29.6641 29.4445 29.3872 28.6903 28.4898 25.3584 24.9193 22.8190 18.7233 14.2554

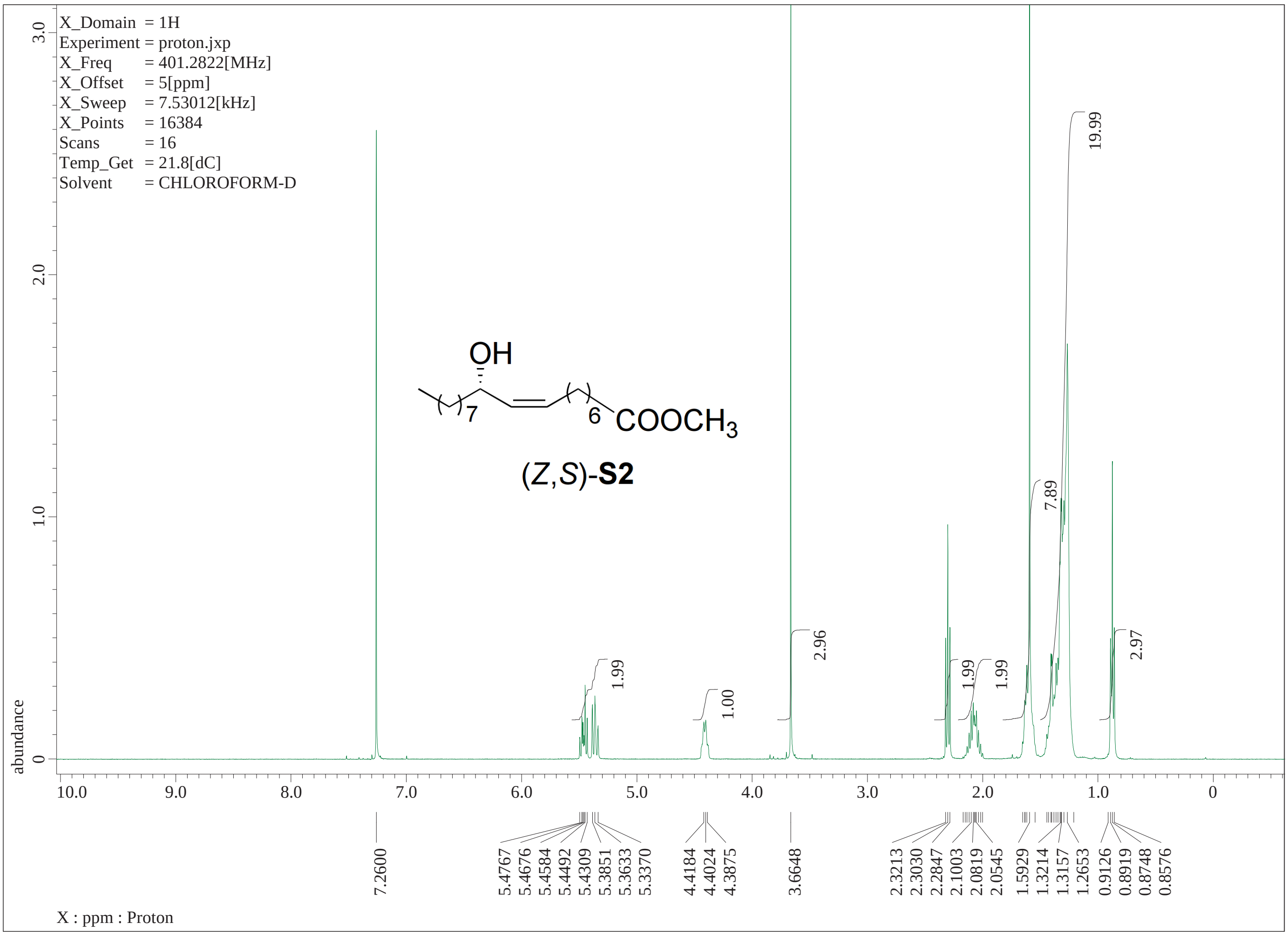
X_Domain = 1H
Experiment = proton.jxp
X_Freq = 401.2822[MHz]
X_Offset = 5[ppm]
X_Sweep = 7.53012[kHz]
X_Points = 16384
Scans = 16
Temp_Get = 21.8[dC]
Solvent = CHLOROFORM-D
OH
7
6
COOCH3
(Z,S)-S2
1.99
1.00
2.96
1.99
1.99
7.89
19.99
2.97
abundance
3.0
2.0
1.0
0
10.0
9.0
8.0
7.0
6.0
5.0
4.0
3.0
2.0
1.0
0
7.2600
5.4767
5.4676
5.4584
5.4492
5.4309
5.3851
5.3633
5.3370
4.4184
4.4024
4.3875
3.6648
2.3213
2.3030
2.2847
2.1003
2.0819
2.0545
1.5929
1.3214
1.3157
1.2653
0.9126
0.8919
0.8748
0.8576
X : ppm : Proton

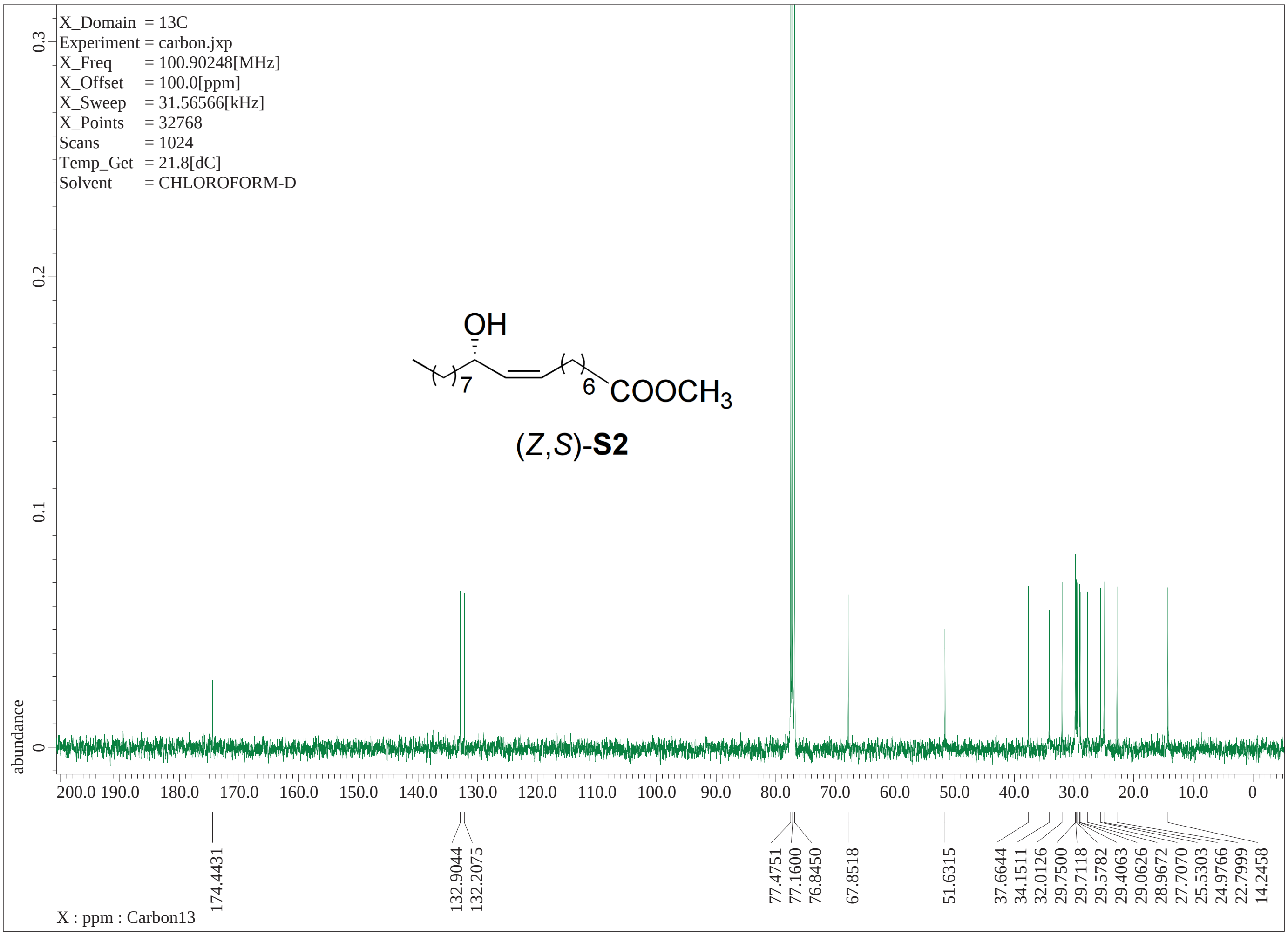
X_Domain = 13C
Experiment = carbon.jxp
X_Freq = 100.90248[MHz]
X_Offset = 100.0[ppm]
X_Sweep = 31.56566[kHz]
X_Points = 32768
Scans = 1024
Temp_Get = 21.8[dC]
Solvent = CHLOROFORM-D
OH
7
6
COOCH3
(Z,S)-S2
abundance
0.3
0.2
0.1
0
200.0
190.0
180.0
170.0
160.0
150.0
140.0
130.0
120.0
110.0
100.0
90.0
80.0
70.0
60.0
50.0
40.0
30.0
20.0
10.0
0
174.4431
132.9044
132.2075
77.4751
77.1600
76.8450
67.8518
51.6315
37.6644
34.1511
32.0126
29.7500
29.7118
29.5782
29.4063
29.0626
28.9672
27.7070
25.5303
24.9766
22.7999
14.2458
X : ppm : Carbon13

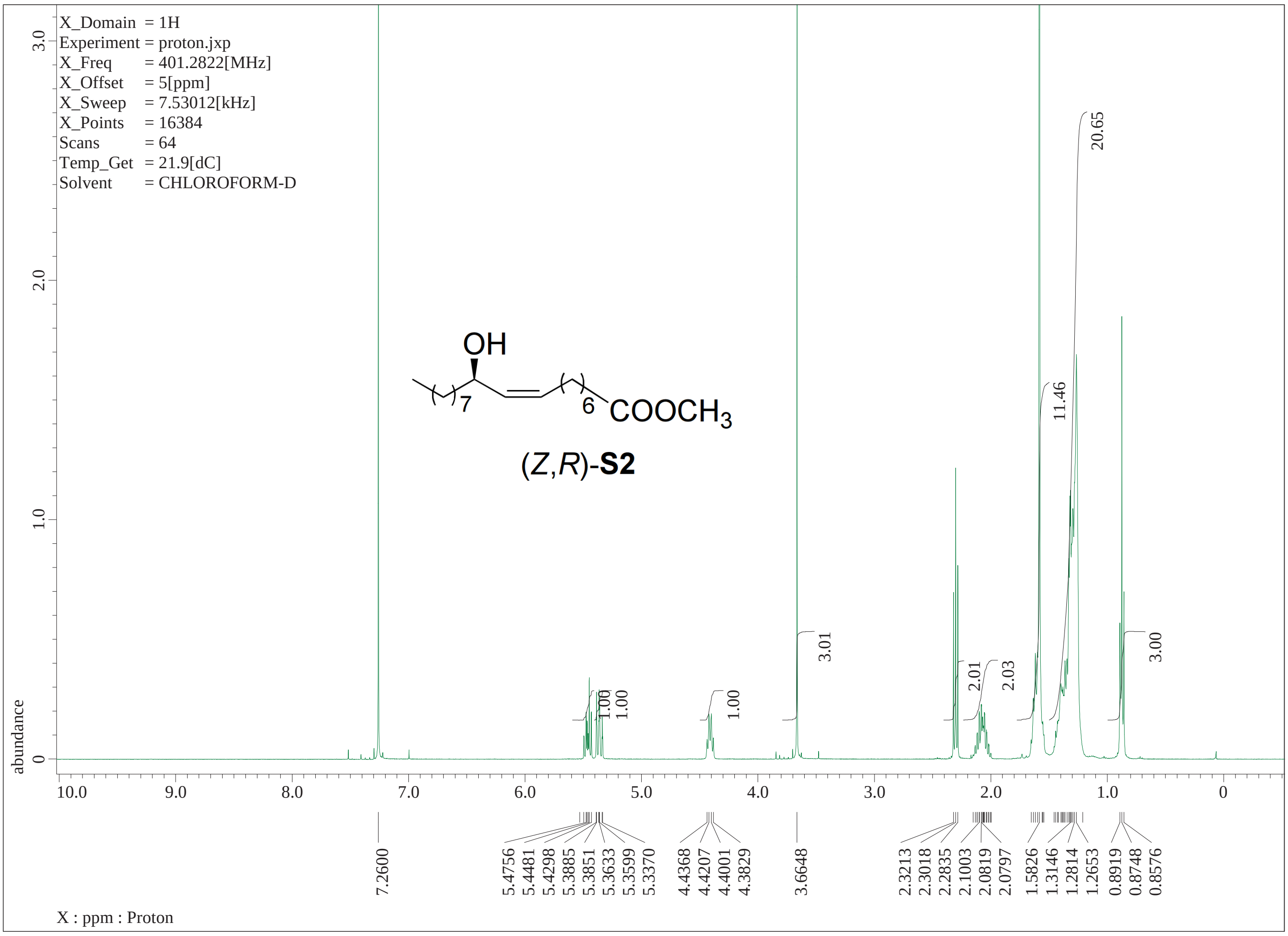
X_Domain = 1H
Experiment = proton.jxp
X_Freq = 401.2822[MHz]
X_Offset = 5[ppm]
X_Sweep = 7.53012[kHz]
X_Points = 16384
Scans = 64
Temp_Get = 21.9[dC]
Solvent = CHLOROFORM-D
OH
7
6
COOCH3
(Z,R)-S2
abundance
1.00
1.00
1.00
3.01
2.01
2.03
11.46
20.65
3.00
7.2600
5.4756
5.4481
5.4298
5.3885
5.3851
5.3633
5.3599
5.3370
4.4368
4.4207
4.4001
4.3829
3.6648
2.3213
2.3018
2.2835
2.1003
2.0819
2.0797
1.5826
1.3146
1.2814
1.2653
0.8919
0.8748
0.8576
X : ppm : Proton

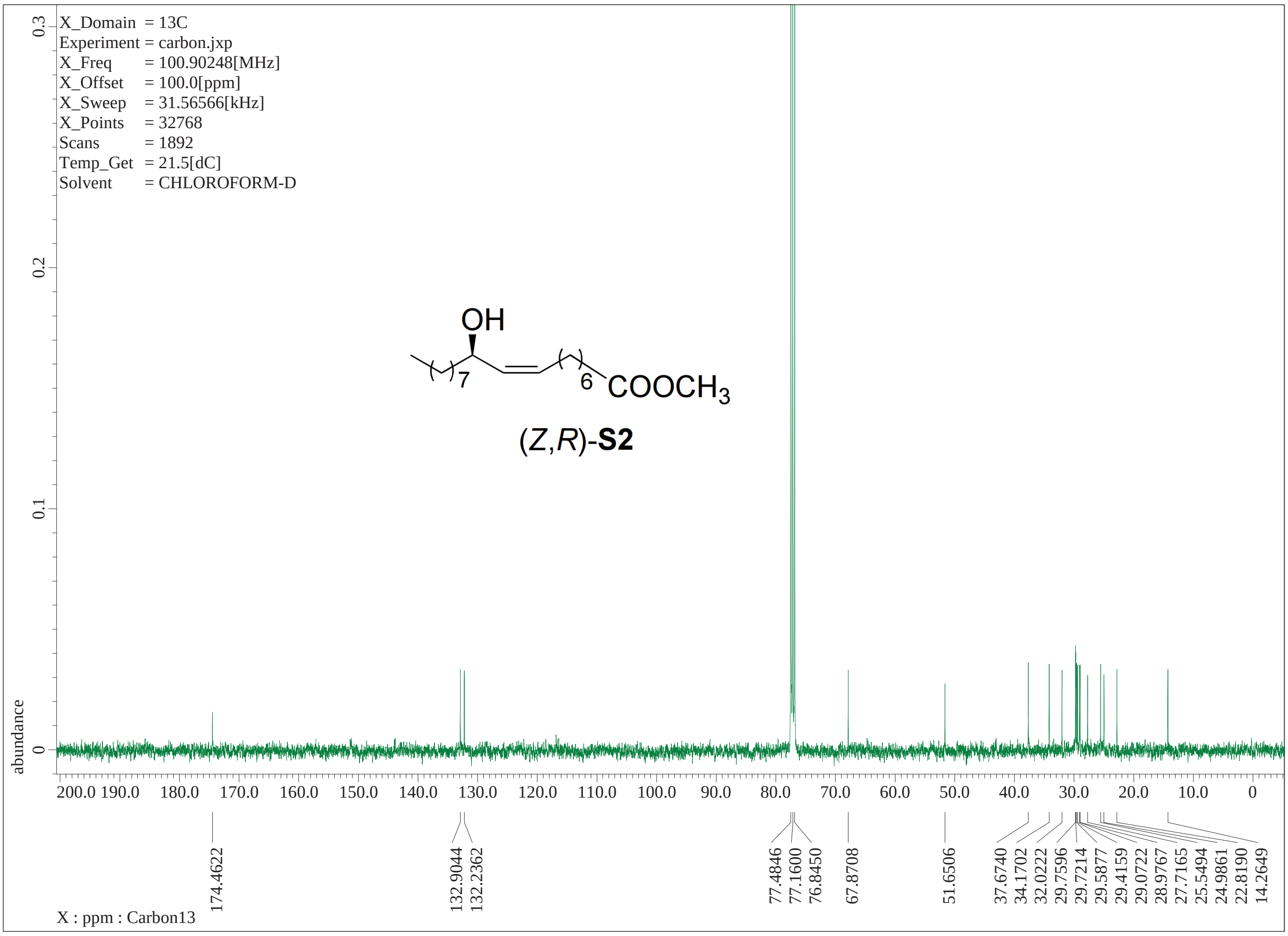
X_Domain = 13C
Experiment = carbon.jxp
X_Freq = 100.90248[MHz]
X_Offset = 100.0[ppm]
X_Sweep = 31.56566[kHz]
X_Points = 32768
Scans = 1892
Temp_Get = 21.5[dC]
Solvent = CHLOROFORM-D
OH
7
6
COOCH3
(Z,R)-S2
abundance
174.4622
132.9044
132.2362
77.4846
77.1600
76.8450
67.8708
51.6506
37.6740
34.1702
32.0222
29.7596
29.7214
29.5877
29.4159
29.0722
28.9767
27.7165
25.5494
24.9861
22.8190
14.2649
X : ppm : Carbon13

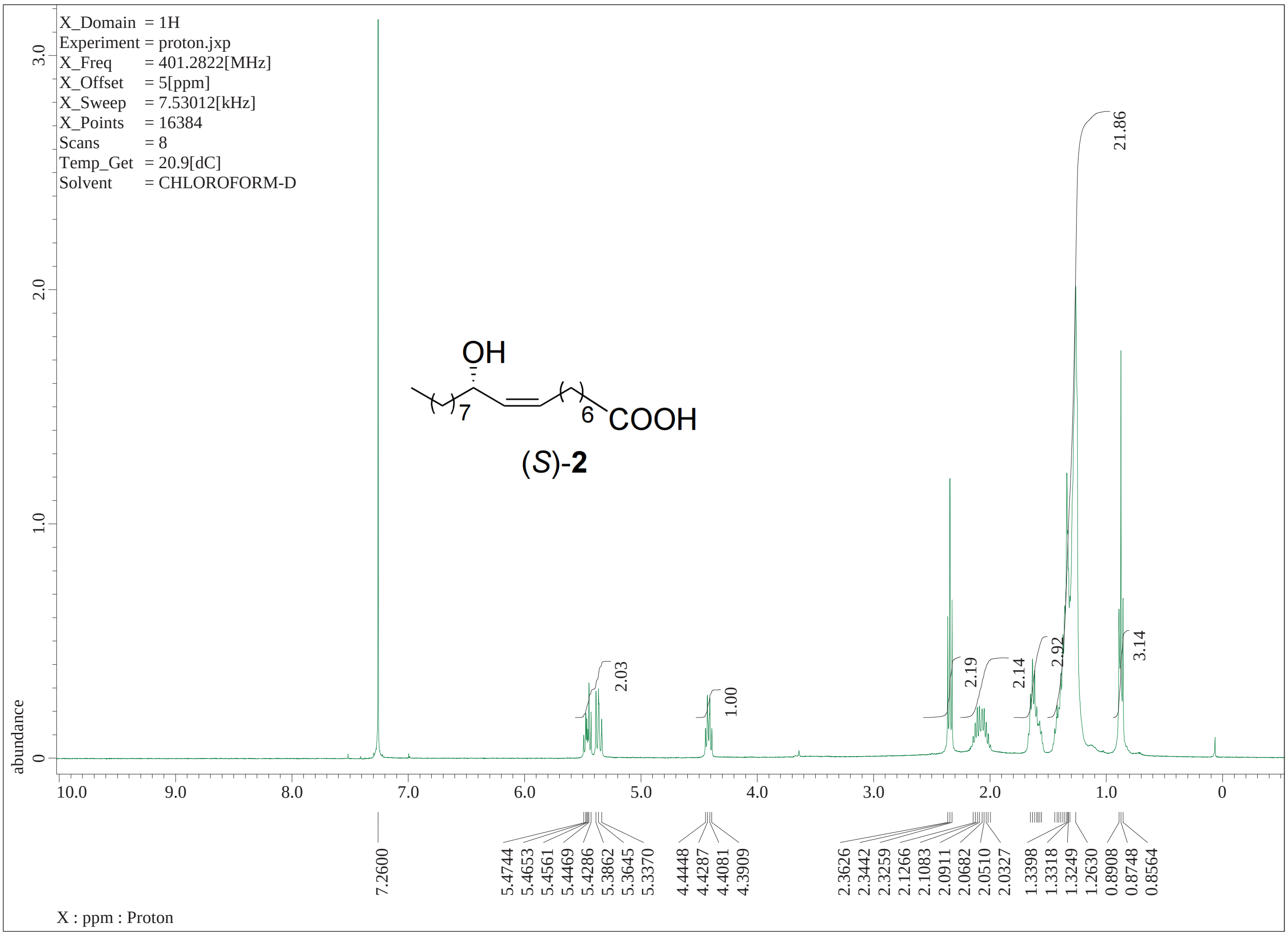
X_Domain = 1H
Experiment = proton.jxp
X_Freq = 401.2822[MHz]
X_Offset = 5[ppm]
X_Sweep = 7.53012[kHz]
X_Points = 16384
Scans = 8
Temp_Get = 20.9[dC]
Solvent = CHLOROFORM-D
OH
7
6
COOH
(S)-2
2.03
1.00
2.19
2.14
2.92
21.86
3.14
abundance
X : ppm : Proton
7.2600
5.4744 5.4653 5.4561 5.4469 5.4286 5.3862 5.3645 5.3370
4.4448 4.4287 4.4081 4.3909
2.3626 2.3442 2.3259 2.1266 2.1083 2.0911 2.0682 2.0510 2.0327
1.3398 1.3318 1.3249 1.2630 0.8908 0.8748 0.8564

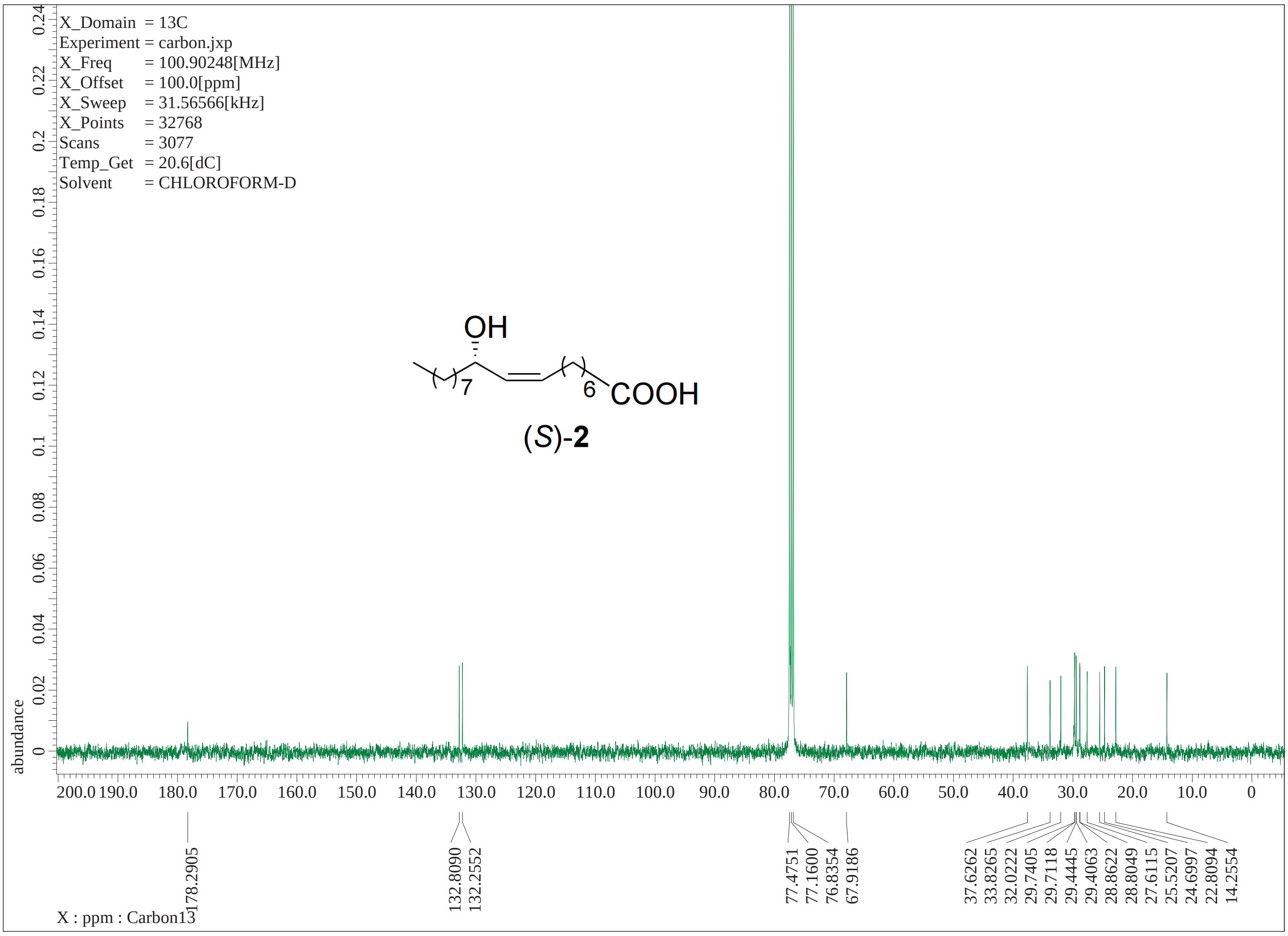
X_Domain = 13C
Experiment = carbon.jxp
X_Freq = 100.90248[MHz]
X_Offset = 100.0[ppm]
X_Sweep = 31.56566[kHz]
X_Points = 32768
Scans = 3077
Temp_Get = 20.6[dC]
Solvent = CHLOROFORM-D
OH
7
6
COOH
(S)-2
abundance
X : ppm : Carbon13
178.2905
132.8090 132.2552
77.4751 77.1600 76.8354 67.9186
37.6262 33.8265 32.0222 29.7405 29.7118 29.4445 29.4063 28.8622 28.8049 27.6115 25.5207 24.6997 22.8094 14.2554

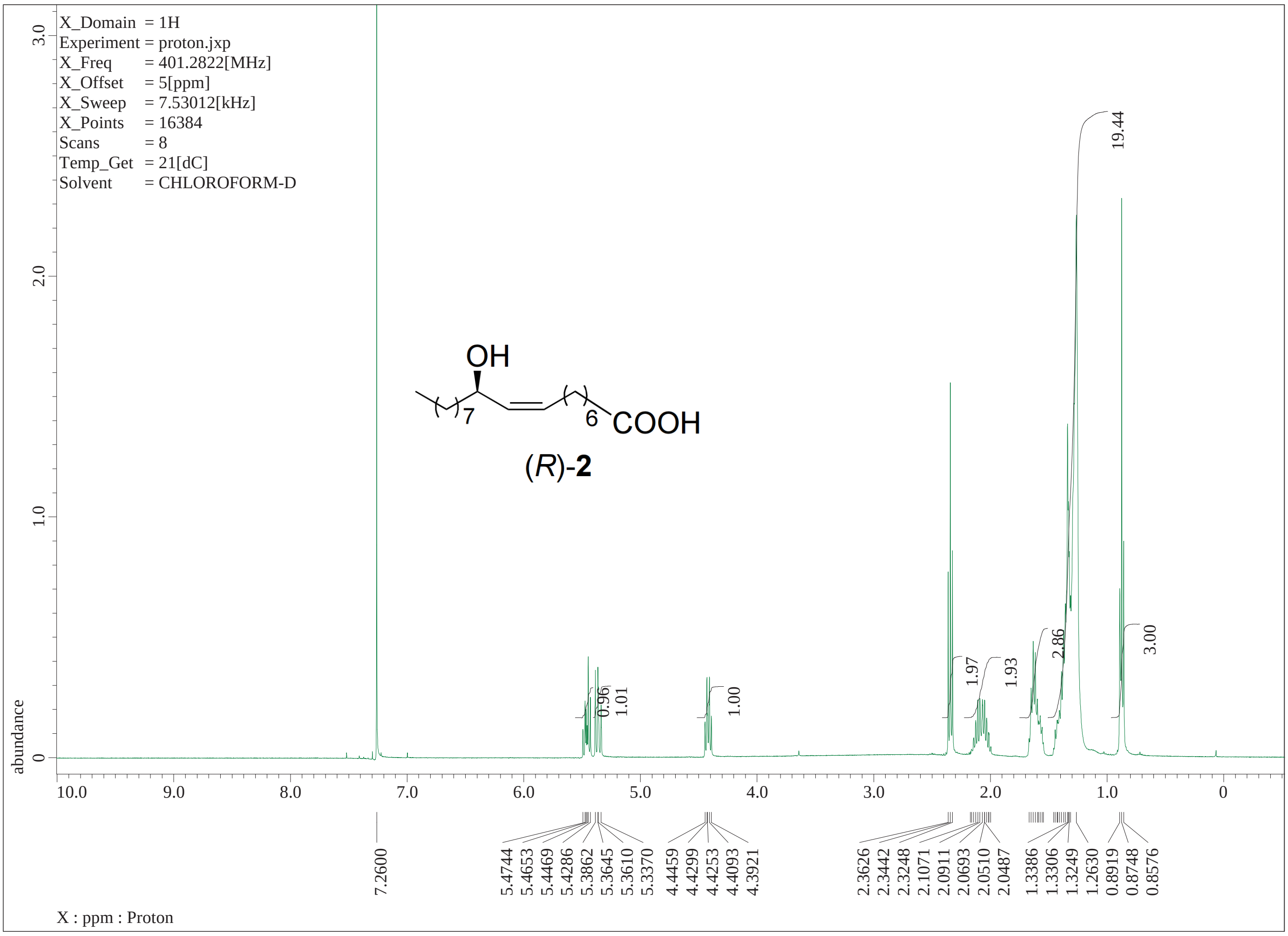

X_Domain = 1H
Experiment = proton.jxp
X_Freq = 401.2822[MHz]
X_Offset = 5[ppm]
X_Sweep = 7.53012[kHz]
X_Points = 16384
Scans = 8
Temp_Get = 21[dC]
Solvent = CHLOROFORM-D
OH
7
6
COOH
(R)-2
19.44
3.00
2.86
1.97
1.93
0.96
1.01
1.00
abundance
7.2600
5.4744
5.4653
5.4469
5.4286
5.3862
5.3645
5.3610
5.3370
4.4459
4.4299
4.4253
4.4093
4.3921
2.3626
2.3442
2.3248
2.1071
2.0911
2.0693
2.0510
2.0487
1.3386
1.3306
1.3249
1.2630
0.8919
0.8748
0.8576
X : ppm : Proton

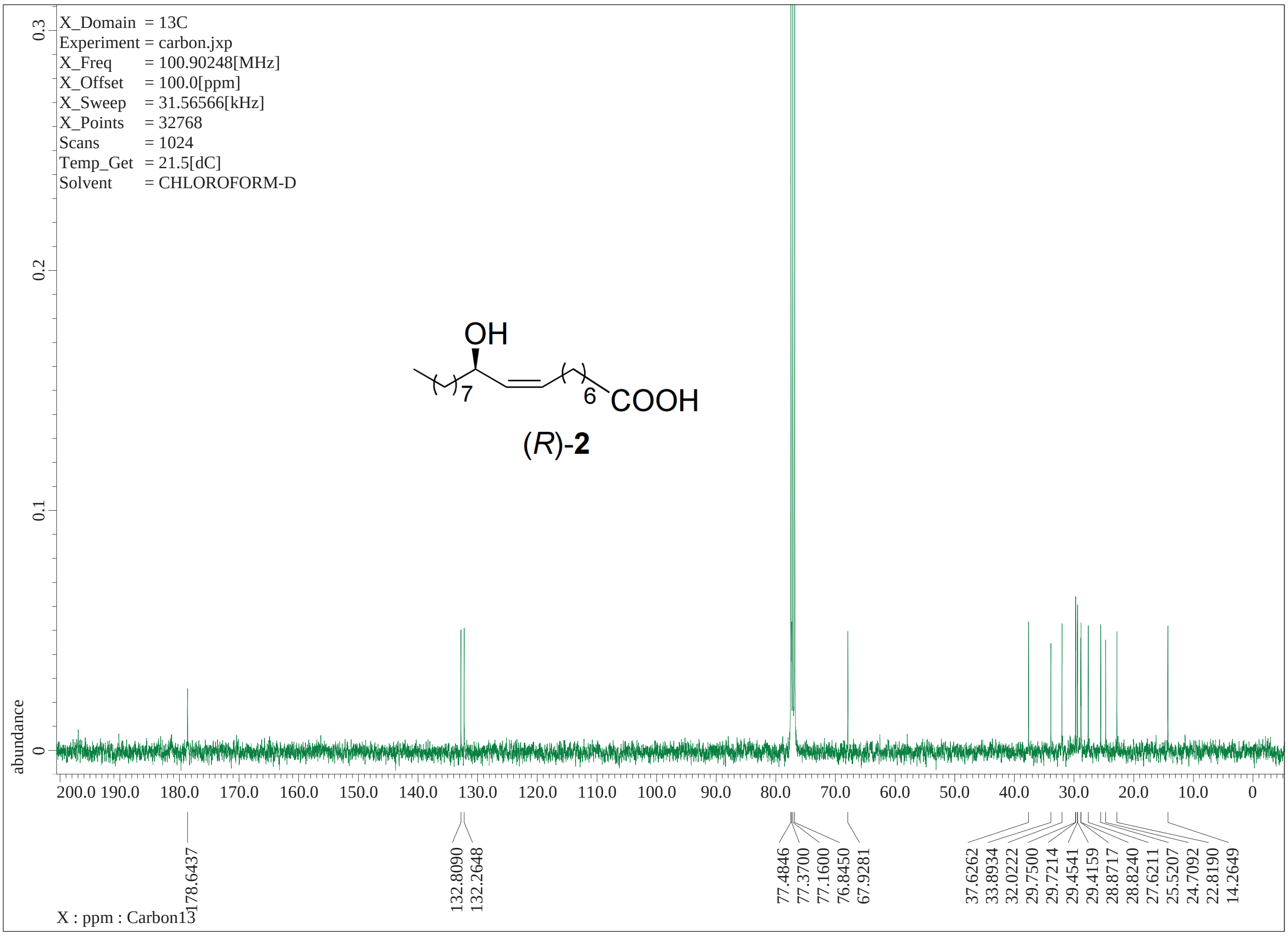

X_Domain = 13C
Experiment = carbon.jxp
X_Freq = 100.90248[MHz]
X_Offset = 100.0[ppm]
X_Sweep = 31.56566[kHz]
X_Points = 32768
Scans = 1024
Temp_Get = 21.5[dC]
Solvent = CHLOROFORM-D
OH
7
6
COOH
(R)-2
abundance
178.6437
132.8090
132.2648
77.4846
77.3700
77.1600
76.8450
67.9281
37.6262
33.8934
32.0222
29.7500
29.7214
29.4541
29.4159
28.8717
28.8240
27.6211
25.5207
24.7092
22.8190
14.2649
X : ppm : Carbon13

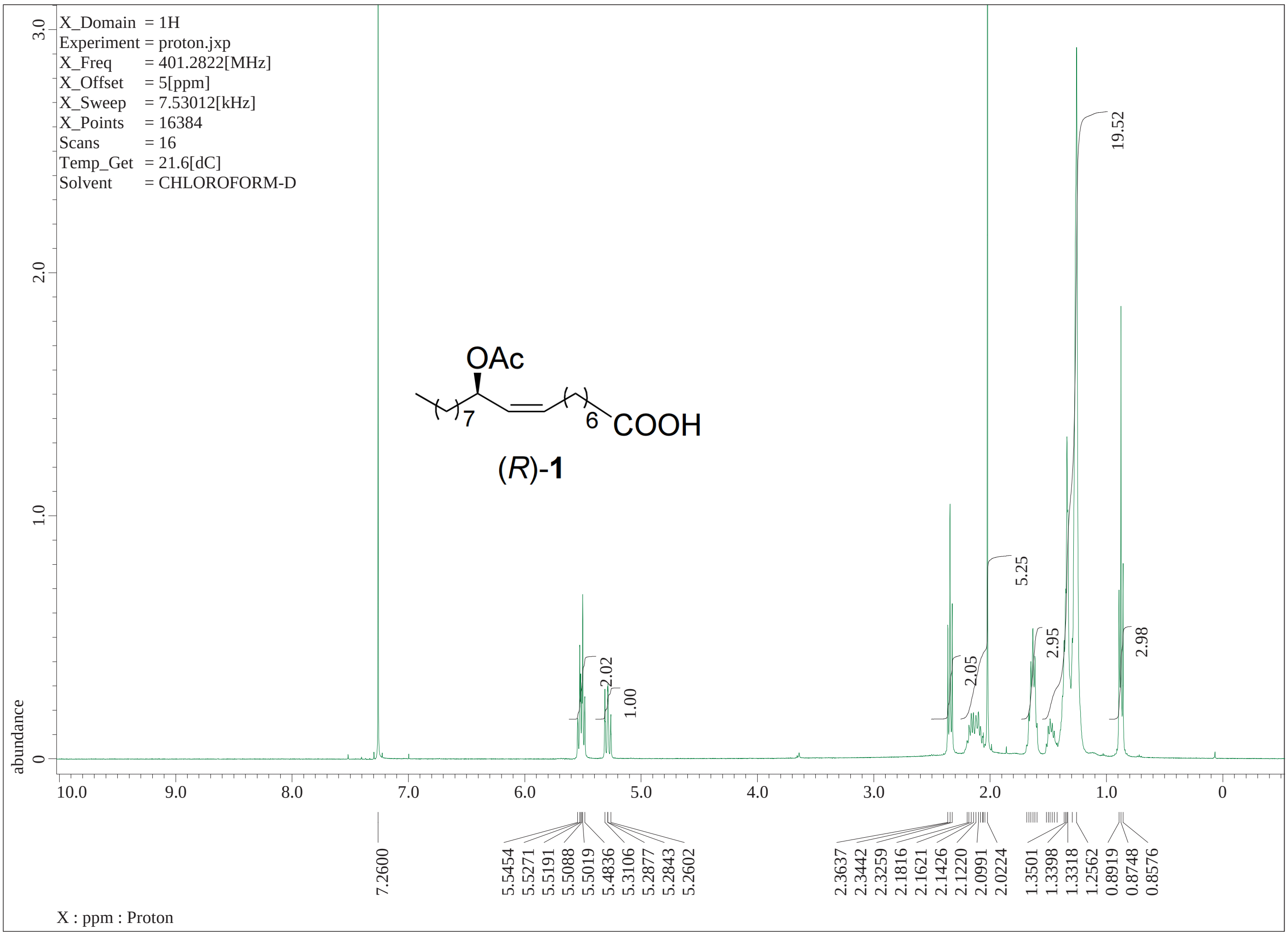
X_Domain = 1H
Experiment = proton.jxp
X_Freq = 401.2822[MHz]
X_Offset = 5[ppm]
X_Sweep = 7.53012[kHz]
X_Points = 16384
Scans = 16
Temp_Get = 21.6[dC]
Solvent = CHLOROFORM-D
OAc
7
6
COOH
(R)-1
abundance
X : ppm : Proton
7.2600
5.5454 5.5271 5.5191 5.5088 5.5019 5.4836 5.3106 5.2877 5.2843 5.2602
2.3637 2.3442 2.3259 2.1816 2.1621 2.1426 2.1220 2.0991 2.0224
1.3501 1.3398 1.3318 1.2562 0.8919 0.8748 0.8576
2.02 1.00 2.05 5.25 2.95 19.52 2.98

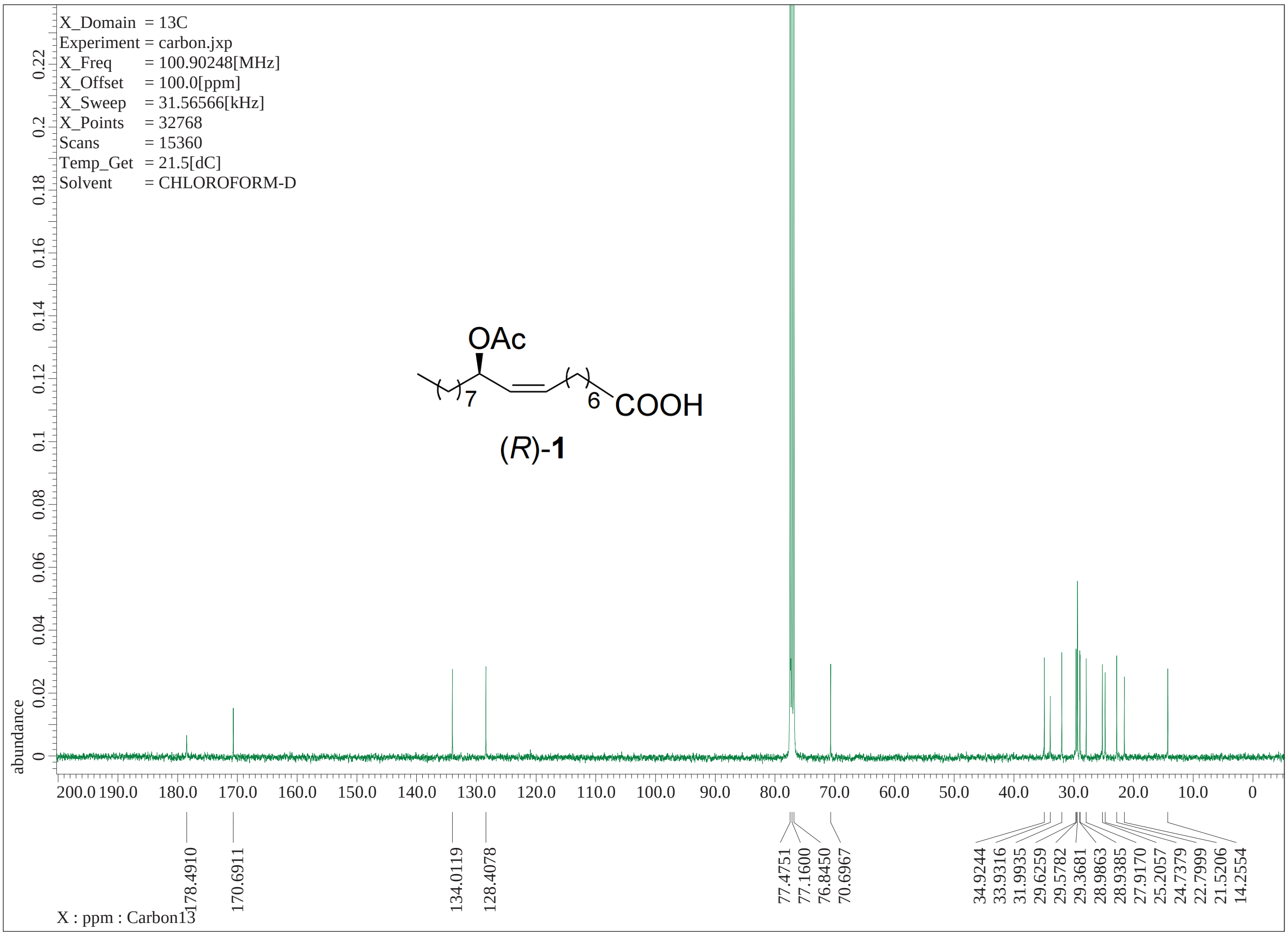
X_Domain = 13C
Experiment = carbon.jxp
X_Freq = 100.90248[MHz]
X_Offset = 100.0[ppm]
X_Sweep = 31.56566[kHz]
X_Points = 32768
Scans = 15360
Temp_Get = 21.5[dC]
Solvent = CHLOROFORM-D
OAc
7
6
COOH
(R)-1
abundance
X : ppm : Carbon13
178.4910 170.6911 134.0119 128.4078
77.4751 77.1600 76.8450 70.6967
34.9244 33.9316 31.9935 29.6259 29.5782 29.3681 28.9863 28.9385 27.9170 25.2057 24.7379 22.7999 21.5206 14.2554
